# Association of Convalescent Plasma Treatment With Clinical Status in Patients Hospitalized With COVID-19: A Meta-analysis

**DOI:** 10.1001/jamanetworkopen.2021.47331

**Published:** 2022-01-25

**Authors:** Andrea B. Troxel, Eva Petkova, Keith Goldfeld, Mengling Liu, Thaddeus Tarpey, Yinxiang Wu, Danni Wu, Anup Agarwal, Cristina Avendaño-Solá, Emma Bainbridge, Katherine J. Bar, Timothy Devos, Rafael F. Duarte, Arvind Gharbharan, Priscilla Y. Hsue, Gunjan Kumar, Annie F. Luetkemeyer, Geert Meyfroidt, André M. Nicola, Aparna Mukherjee, Mila B. Ortigoza, Liise-anne Pirofski, Bart J. A. Rijnders, Casper Rokx, Arantxa Sancho-Lopez, Pamela Shaw, Pablo Tebas, Hyun-Ah Yoon, Corita Grudzen, Judith Hochman, Elliott M. Antman

**Affiliations:** 1 Department of Population Health, NYU Grossman School of Medicine, New York, New York; 2 Department of Child and Adolescent Psychiatry, NYU Grossman School of Medicine, New York, New York; 3 The Nathan S. Kline Institute for Psychiatric Research, Orangeburg, New York; 4 Department of Environmental Health, NYU Grossman School of Medicine, New York, New York; 5 Department of Biostatistics, University of Washington School of Public Health, Seattle; 6 Indian Council of Medical Research, New Delhi, Delhi, India; 7 Hospital Universitario Puerta de Hierro Majadahonda, Madrid, Spain; 8 Zuckerberg San Francisco General, University of California San Francisco, San Francisco; 9 Department of Medicine, University of Pennsylvania Perelman School of Medicine, Philadelphia; 10 Department of Hematology, University Hospitals Leuven and Department of Microbiology and Immunology, Laboratory of Molecular Immunology (Rega Institute), KU Leuven, Leuven, Belgium; 11 Section of Infectious Diseases, Department of Internal Medicine, Erasmus University Medical Center, Rotterdam, the Netherlands; 12 Department of Intensive Care Medicine, University Hospitals Leuven, Leuven, Belgium; 13 Hospital Universitário de Brasília, University of Brasília, Brasília, Brazil; 14 Department of Medicine, NYU Grossman School of Medicine, New York, New York; 15 Department of Microbiology, NYU Grossman School of Medicine, New York, New York; 16 Department of Medicine, Albert Einstein College of Medicine, Bronx, New York; 17 Department of Microbiology and Immunology, Albert Einstein College of Medicine, Bronx, New York; 18 Biostatistics Unit, Kaiser Permanente Washington Health Research Institute, Seattle; 19 Department of Emergency Medicine, NYU Grossman School of Medicine, New York, New York; 20 Department of Medicine, NYU Grossman School of Medicine, New York, New York; 21 Brigham and Women’s Hospital, Harvard Medical School, Boston, Massachusetts

## Abstract

**Importance:**

COVID-19 convalescent plasma (CCP) is a potentially beneficial treatment for COVID-19 that requires rigorous testing.

**Objective:**

To compile individual patient data from randomized clinical trials of CCP and to monitor the data until completion or until accumulated evidence enables reliable conclusions regarding the clinical outcomes associated with CCP.

**Data Sources:**

From May to August 2020, a systematic search was performed for trials of CCP in the literature, clinical trial registry sites, and medRxiv. Domain experts at local, national, and international organizations were consulted regularly.

**Study Selection:**

Eligible trials enrolled hospitalized patients with confirmed COVID-19, not receiving mechanical ventilation, and randomized them to CCP or control. The administered CCP was required to have measurable antibodies assessed locally.

**Data Extraction and Synthesis:**

A minimal data set was submitted regularly via a secure portal, analyzed using a prespecified bayesian statistical plan, and reviewed frequently by a collective data and safety monitoring board.

**Main Outcomes and Measures:**

Prespecified coprimary end points—the World Health Organization (WHO) 11-point ordinal scale analyzed using a proportional odds model and a binary indicator of WHO score of 7 or higher capturing the most severe outcomes including mechanical ventilation through death and analyzed using a logistic model—were assessed clinically at 14 days after randomization.

**Results:**

Eight international trials collectively enrolled 2369 participants (1138 randomized to control and 1231 randomized to CCP). A total of 2341 participants (median [IQR] age, 60 [50-72] years; 845 women [35.7%]) had primary outcome data as of April 2021. The median (IQR) of the ordinal WHO scale was 3 (3-6); the cumulative OR was 0.94 (95% credible interval [CrI], 0.74-1.19; posterior probability of OR <1 of 71%). A total of 352 patients (15%) had WHO score greater than or equal to 7; the OR was 0.94 (95% CrI, 0.69-1.30; posterior probability of OR <1 of 65%). Adjusted for baseline covariates, the ORs for mortality were 0.88 at day 14 (95% CrI, 0.61-1.26; posterior probability of OR <1 of 77%) and 0.85 at day 28 (95% CrI, 0.62-1.18; posterior probability of OR <1 of 84%). Heterogeneity of treatment effect sizes was observed across an array of baseline characteristics.

**Conclusions and Relevance:**

This meta-analysis found no association of CCP with better clinical outcomes for the typical patient. These findings suggest that real-time individual patient data pooling and meta-analysis during a pandemic are feasible, offering a model for future research and providing a rich data resource.

## Key Points

**Question:**

What is the pooled evidence from high-quality randomized clinical trials regarding the safety and potential benefit of convalescent plasma to treat hospitalized patients with COVID-19?

**Findings:**

In this meta-analysis of 8 randomized clinical trials enrolling 2341 participants, individual patient data were monitored in real time and analyzed using a robust bayesian framework and advanced statistical modeling. No association of convalescent plasma with clinical outcomes was found.

**Meaning:**

These findings suggest that real-time individual patient data pooling and meta-analysis during a pandemic are feasible, offering a model for future research and providing a rich data resource.

## Teaser

This meta-analysis examines individual patient data from 8 randomized clinical trials of COVID-19 convalescent plasma to determine its safety and efficacy in treating hospitalized patients with COVID-19.

## Introduction

The COVID-19 pandemic has created a humanitarian crisis.^1,2^ Identifying safe and effective therapies is challenging given the shifting outbreak locations, disparate efforts to conduct randomized clinical trials (RCTs), and open-label emergency use of treatments.^3,4^ Several approaches to hastening progress have been proposed,^5^ including launching trials in hot spots, instituting platform designs,^6^ and synthesizing data from multiple RCTs. Meta-analyses typically pool data from completed RCTs^7,8^; another approach involves pooling data from trials in various stages, some completed and others continuing enrollment.^9^ Because the complexity of the pandemic might be associated with the outcomes of potential therapies, it is essential to analyze individual patient data (IPD) rather than trial summaries.^10^ We implemented a practical approach to nearly real-time pooling of IPD from completed and ongoing RCTs^11,12,13,14,15,16,17,18^ of COVID-19 convalescent plasma (CCP) and report here the results of the COMPILE (COntinuous Monitoring of Pooled International Trials of ConvaLEscent Plasma for COVID-19 Hospitalized Patients) study.^4^

Potential therapies for COVID-19 may not offer similar benefit across populations. Monoclonal antibody therapies^19,20^ are promising for outpatients, remdesivir shortens recovery time in hospitalized patients,^21^ and dexamethasone reduces mortality in hospitalized patients requiring supplemental oxygen.^22^ The COMPILE study focused on hospitalized patients with documented COVID-19 not requiring mechanical ventilation^23^; passive immunization with CCP is most likely to be effective in patients before progression to advanced stages,^2,23,24,25,26,27^ and timing of therapy may be associated with viral load and the hyperimmune response.^23,25,28^

We pooled deidentified IPD from RCTs^11,12,13,14,15,16,17,18^ collaborating in the COMPILE study to provide evidence with a high degree of certainty regarding the benefit (or harm) and safety of CCP in hospitalized patients with COVID-19.^4^ Our objective was to regularly update and frequently monitor the accumulating data until trial completion or until sufficient evidence enabled reliable and convincing conclusions regarding CCP in the target population. A minimal data set of deidentified IPD from each participating RCT was submitted regularly via secure file transfer protocol, analyzed using a prespecified bayesian statistical plan, and reviewed frequently by a collective Data and Safety Monitoring Board (cDSMB). We prioritized the dual goals of providing sufficient information to regulatory authorities to formulate policies on the use of CCP in patients with COVID-19 and providing the clinical community with evidence to target CCP use to those most likely to benefit.

## Methods

### Real-Time IPD Meta-analysis

The NYU institutional review board determined that this meta-analysis was exempt because the data were deidentified. This report follows the Preferred Reporting Items for Systematic Reviews and Meta-analyses (PRISMA) IPD reporting guideline.^29^ From May to August 2020, we systematically searched for trials of CCP for COVID-19 in the literature (and their references), clinical trial registry sites (ClinicalTrials.gov,^30^ Chinese Clinical Trial Registry,^31^ and EU Clinical Trials Register^32^), and medRxiv^33^; search terms included *plasma*, *convalescent plasma*, *survivor’s plasma*, *blood plasma*, *passive immunity*, *clinical trials*, *COVID-19*, and *SARS-CoV-2*. We also consulted regularly from May to December 2020 with local, national, and international domain experts. The trials were required to enroll hospitalized patients with a confirmed COVID-19 diagnosis via polymerase chain reaction or antigen test, not receiving mechanical ventilation, and randomized to receive CCP or control; all participants provided written informed consent. The administered CCP was required to have measurable antibodies determined locally with a qualitative or quantitative assay. Investigators from qualifying RCTs were invited to join COMPILE; those who agreed provided data for this report. Completed, early terminated, or ongoing RCTs could be added in a rolling fashion.

### Operations

The COMPILE Steering Committee, comprising principal investigators of the qualifying RCTs, met regularly to review progress. The cDSMB, comprising the chairs and unblinded statisticians of each RCT-specific DSMB, met at least monthly to review ongoing analyses prepared by a team of unblinded statisticians at NYU. A secure data transfer and file sharing system was accessible by constituent RCT members. Committee rosters, governance documents, and additional details are available in eAppendix 1, eAppendix 2, eAppendix 3, and eAppendix 4 in the Supplement.

### Outcomes

The COMPILE protocol prespecified coprimary end points, both based on the World Health Organization (WHO) 11-point clinical scale^34^ (eFigure 1 in the Supplement) measured by clinical staff at 14 ± 1 days after randomization (hereafter, day 14): the full 11-point WHO ordinal score (analyzed using a proportional odds model) and a binary indicator defined as a WHO score of 7 to 10 vs less than 7 (analyzed using a logistic model), where a higher score indicates a worse clinical outcome. The former was chosen for maximum information use and the latter for easier interpretability; details of the statistical models are provided later in this section. The secondary outcomes were the 11-point WHO score and the binary indicator (WHO score ≥7) measured at 28 ± 2 days after randomization (hereafter, day 28). Patients discharged from the hospital before day 14 were contacted to ascertain WHO score at days 14 and 28. Tertiary outcomes were mortality (WHO score, 10) at days 14 and 28 and time to death and discharge. Safety outcomes included transfusion-related acute lung injury, transfusion-associated circulatory overload, possible transfusion-related acute lung injury or transfusion-associated circulatory overload undifferentiated from COVID-19 disease, and venous or arterial thrombotic events.

### Statistical Analysis

COMPILE conducted a bayesian meta-analysis of IPD and used bayesian monitoring based on estimation of parameters with credible intervals (CrIs) rather than on frequentist hypothesis testing; in this paradigm, type I error control is less relevant. We focused on posterior probabilities of odds ratio (OR) estimates of a certain direction and size.^35,36,37^ The statistical analysis plan was supported by extensive simulations to understand the impact of prior distributions and other modeling choices and to approximate the conventional frequentist operating characteristics that could be expected with application of our stopping rules.^38^ There was no predetermined sample size; trials that were still ongoing during the project continued to accrue participants. Analyses were performed using R statistical software version 4.1.1 (R Project for Statistical Computing)^39^ and Stan statistical software version 2.28 (Stan Development Team).^40^

#### Primary Analyses

The primary outcomes were analyzed with bayesian models, using a cumulative proportional odds model for the WHO 11-point scale and a logistic regression model for the binary WHO status of scores of 7 to 10 vs less than 7. We adjusted for a parsimonious set of covariates (age, sex, WHO status at baseline, duration of symptoms before randomization, and calendar quarter of enrollment) and incorporated study-specific random effects and indicator variables to address the 3 different control conditions: standard of care, nonconvalescent plasma, or saline solution.^38^ The models included study-specific and control-specific CCP parameters, and an overall parameter for CCP compared with any control (eAppendix 1 in the Supplement). The overarching modeling philosophy was to use skeptical priors for outcome measures and less skeptical priors for safety measures, use minimally informative priors for parameters that are not associated with decision-making but require estimation, and be flexible regarding nuisance parameters to ensure stable model fitting.^38^ We used the posterior distributions of the model parameters to generate estimates of the pooled ORs for CCP compared with control, their associated 95% CrIs, and posterior probabilities of conditions of interest (eg, probability of OR <1). eAppendix 1 in the Supplement provides a brief explanation of bayesian inference.

#### Secondary and Tertiary Analyses

Additional analyses of the primary outcomes used similar models, but adjusted for an expanded set of covariates (eTable 1 in the Supplement). Similar analyses were used for secondary outcomes measured at day 28 and for tertiary outcomes of mortality at days 14 and 28. Unadjusted tertiary analyses used Kaplan-Meier estimates of mortality, comparing treatment groups with a stratified log-rank test, and estimated competing-risk adjusted cumulative incidence of time to discharge,^41^ comparing treatment groups with the Gray test^42^; we applied a 2-sided type I error rate of .05 to each.

In subgroup analyses, we assessed the association of CCP with outcomes within prespecified subgroups, based on age, sex, baseline WHO score, and duration of symptoms before randomization, using the aforementioned bayesian models. In sensitivity analysis, we investigated the sensitivity of inferences to different approaches for missing outcomes (WHO scores at day 14 and 28) and to the hypothetical scenario that another large CCP RCT became available (eAppendix 1 in the Supplement). The Cochrane Risk of Bias assessment was completed.^43^

The bayesian monitoring plan defined straightforward, actionable rules for efficacy, harm, and safety, incorporating information accrued across all studies; details are provided in eAppendix 2, eAppendix 3, and eAppendix 4 in the Supplement. A treatment benefit index is a combination of pretreatment characteristics that identifies participants who are likely to benefit and the degree of benefit from a specific treatment. The COMPILE protocol and statistical analysis plan prespecified identification of a treatment benefit index for CCP treatment; results are reported in Park et al.^44^

## Results

### Participating Trials

Database lock for this report occurred on April 19, 2021, at which time all participating trials had either completed or terminated enrollment and outcome data were deemed as complete as possible. Table 1 provides the characteristics of the 8 participating RCTs from Asia, Europe, North America, and South America; 2 were double-blinded and 6 were open label; 3 were single-site and 5 were multisite.^11,12,13,14,15,16,17,18^ The control conditions were standard of care (6 RCTs), nonconvalescent plasma (1 RCT), or saline solution (1 RCT). Six RCTs enrolled participants with WHO score at baseline of 4 to 6; 2 RCTs included only participants with a score of 5 to 6 at baseline. eFigure 2 in the Supplement provides a ring diagram indicating the compilation of participants across RCTs. eAppendix 4 in the Supplement provides details about each trial.

**Table 1.** RCTs Participating in COMPILE

Control condition and RCT	CCP units, No.	Patients, No. (N = 2369)	
		Control (n = 1138)	CCP (n = 1231)
Saline, Ortigoza et al^11^ (n = 941)	1	473	468
Nonconvalescent plasma, Hsue et al^16^ (n = 34)	1	18	16
Standard of care (n = 1394)			
Bar et al^12^ (n = 80)	2	39	41
Avendaño-Solá et al^13^ (n = 350)	1	171	179
Devos et al^14^ (n = 477)	4	163	314
Nicola^17^ (n = 34)	1	15	19
Agarwal et al^15^ (n = 381)	2	224	157
Rijnders^18^ (n = 72)	1	35	37

Abbreviations: CCP, COVID-19 convalescent plasma; RCT, randomized clinical trial.

### Participants

Altogether, 2369 participants met trial eligibility; 1138 were randomized to control and 1231 to CCP. Table 2 describes the baseline characteristics of pooled participants by treatment group; baseline characteristics by RCT are provided in eTable 2 in the Supplement. Among the 2369 participants, the median (IQR) age was 60 (50-72) years, and 845 (35.7%) were women. More than half of participants were randomized 4 to 10 days from onset of symptoms. There were 452 patients (19.1%) with a baseline WHO score of 4, 1501 patients (63.4%) with a baseline WHO score of 5, and 416 patients (17.6%) with a baseline WHO score of 6. The median (IQR) of the ordinal WHO scale was 3 (3-6). Common preexisting conditions included diabetes (795 patients [33.6%]), cardiovascular disease (1008 patients [42.5%]), and pulmonary disease (280 patients [11.8%]).

**Table 2.** Baseline Characteristics Pooled Across All Randomized Clinical Trials

Baseline characteristics	Participants, No. (%)		
	Control (n = 1138)	CCP (n = 1231)	Overall (N = 2369)
Age, median (IQR), y	60 (50-72)	61 (50-71)	60 (50-72)
Sex			
Female	408 (35.9)	437 (35.5)	845 (35.7)
Male	730 (64.1)	794 (64.5)	1524 (64.3)
Baseline World Health Organization severity score			
4 (hospitalized, no O_2_)	235 (20.7)	217 (17.6)	452 (19.1)
5 (hospitalized, O_2_ by mask or nasal)	701 (61.6)	800 (65.0)	1501 (63.4)
6 (hospitalized, O_2_ by noninvasive ventilation)	202 (17.8)	214 (17.4)	416 (17.6)
Blood group			
O	518 (45.5)	568 (46.1)	1086 (45.8)
A	374 (32.9)	420 (34.1)	794 (33.5)
B	195 (17.1)	181 (14.7)	376 (15.9)
AB	38 (3.3)	54 (4.4)	92 (3.9)
NA	13 (1.1)	8 (0.6)	21 (0.9)
Time since symptoms onset, d			
0-3	142 (12.5)	148 (12.0)	290 (12.2)
4-6	394 (34.6)	441 (35.8)	835 (35.2)
7-10	402 (35.3)	431 (35.0)	833 (35.2)
11-14	136 (12.0)	125 (10.2)	261 (11.0)
>14	58 (5.1)	74 (6.0)	132 (5.6)
NA	6 (0.5)	12 (1.0)	18 (0.8)
Time since COVID-19 diagnosis at randomization, median (IQR), d	2 (1-3)	1 (1-3)	2 (1-3)
Medical history			
Diabetes			
No	770 (67.7)	804 (65.3)	1574 (66.4)
Yes	368 (32.3)	427 (34.7)	795 (33.6)
NA	0 (0.0)	0 (0.0)	0 (0.0)
Pulmonary disease			
No	998 (87.7)	1082 (87.9)	2080 (87.8)
Yes	136 (12.0)	144 (11.7)	280 (11.8)
NA	4 (0.4)	5 (0.4)	9 (0.4)
Cardiovascular disease			
No	660 (58.0)	694 (56.4)	1354 (57.2)
Yes	474 (41.7)	534 (43.4)	1008 (42.5)
NA	4 (0.4)	3 (0.2)	7 (0.3)
Enrollment quarter			
April-June 2020	344 (30.2)	297 (24.1)	641 (27.1)
July-September 2020	215 (18.9)	242 (19.7)	457 (19.3)
October-December 2020	405 (35.6)	504 (40.9)	909 (38.4)
January-March 2021	174 (15.3)	188 (15.3)	362 (15.3)

Abbreviations: CCP, COVID-19 convalescent plasma; NA, not available.

### Primary Outcomes

**Figure 1.**
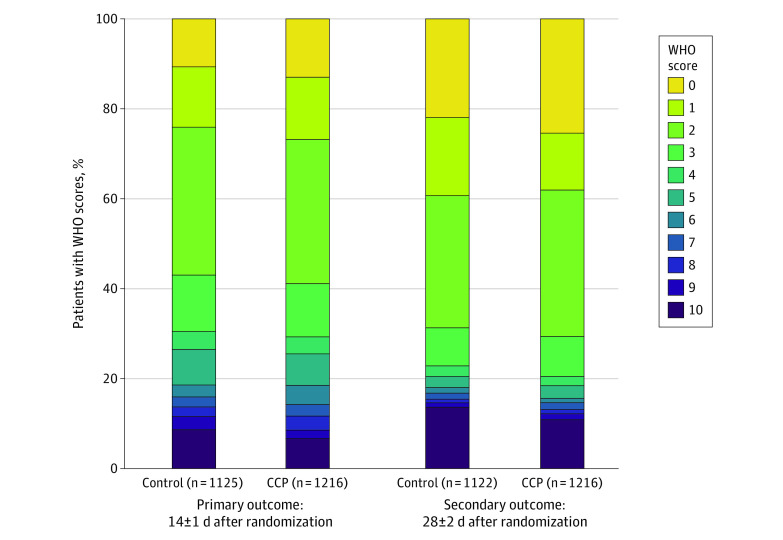
Proportion of Participants at Different Clinical Stages of COVID-19 Measured on the World Health Organization (WHO) 11-Point Scale at Days 14 and 28 by Treatment Group CCP indicates COVID-19 convalescent plasma.

**Figure 2.**
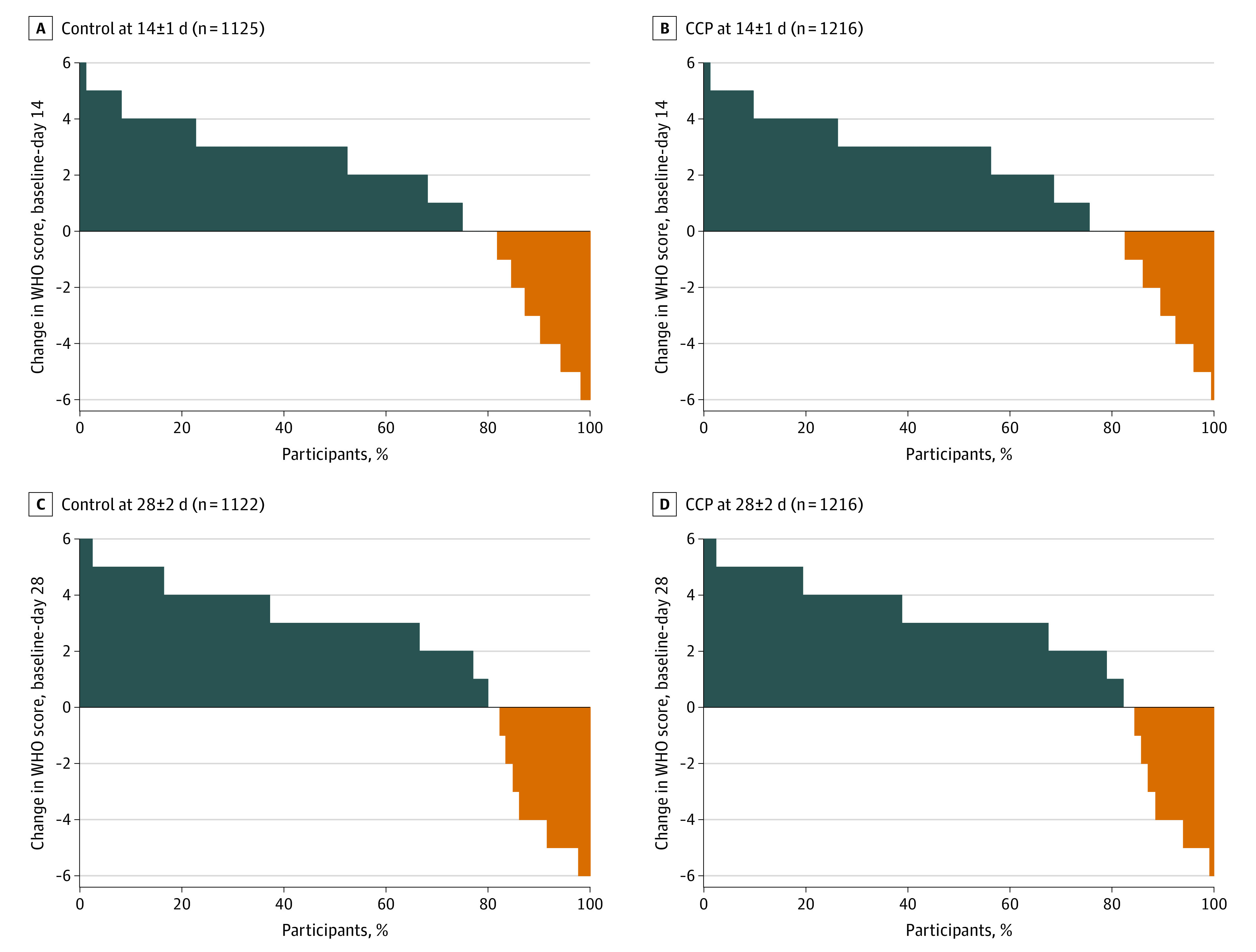
Change in World Health Organization (WHO) Scores by Treatment Positive change scores indicate improvement from baseline and are shown in blue; negative change scores indicate worsening and are shown in orange. The abscissa shows the percentage of patients with different changes in scores. Larger blue areas and smaller orange areas in the COVID-19 convalescent plasma (CCP) group compared with control are indicative of CCP association with better outcomes.

Among 2341 patients whose primary outcome was obtained, 38 patients were discharged from the hospital before day 14 and could not be contacted; we imputed their outcomes using their WHO score at discharge. Seventeen patients had missing data on parsimonious covariates, and 32 more were excluded from secondary analyses for missing expanded covariates. eFigure 3 in the Supplement provides the CONSORT diagram. Figure 1 shows the distributions of the WHO scores at day 14; 253 participants (15.0%) had 1 WHO score of 7 to 10, including 179 participants (15.8%) in the control group and 173 participants (14.2%) in the CCP group. Figure 2 presents the distribution of the change in scores (baseline to day 14) by treatment.

**Figure 3.**
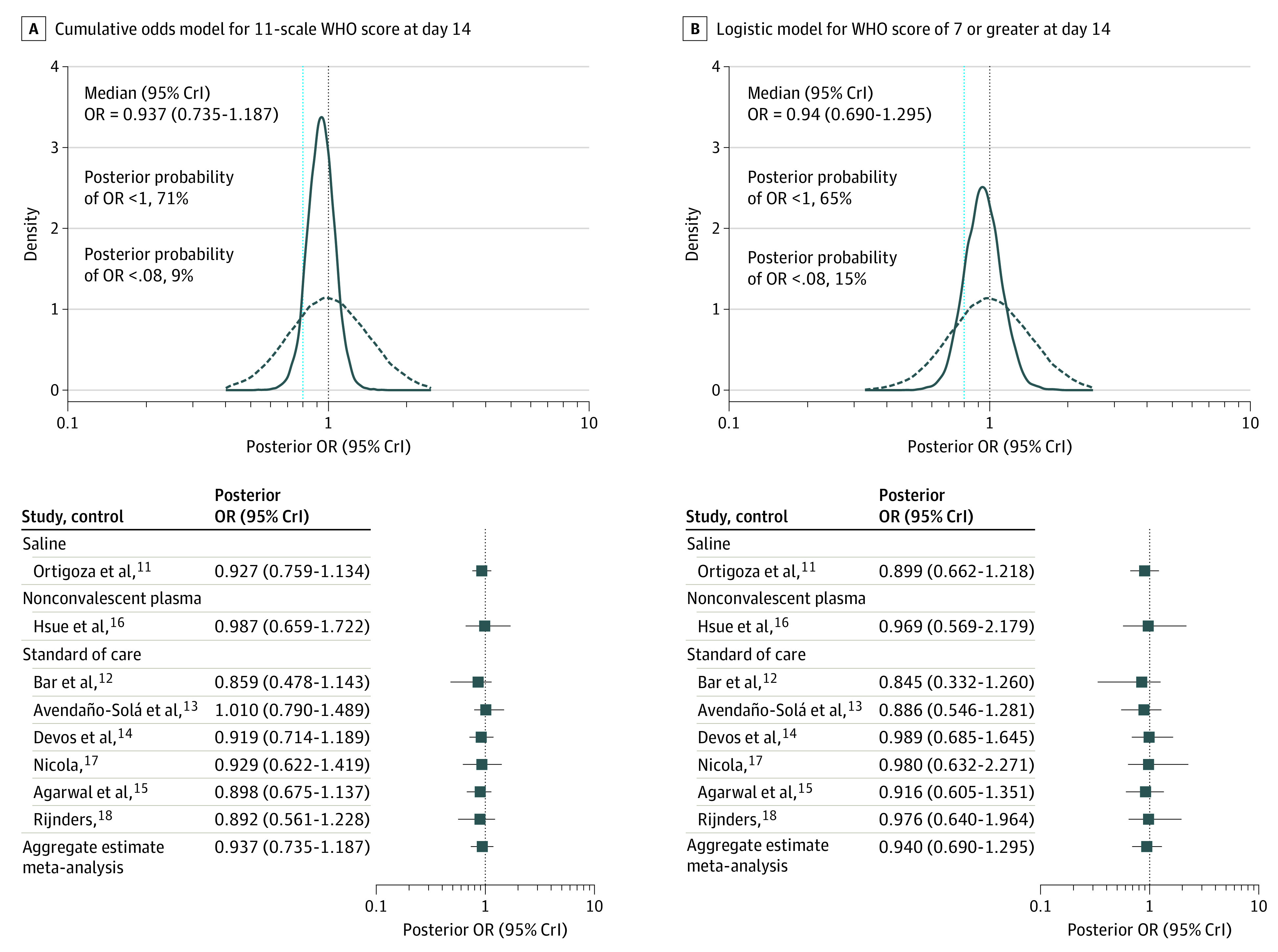
Posterior Distribution of the Odds Ratios (ORs) From the Cumulative Odds Model for World Health Organization (WHO) Scores and the Binary Outcome of WHO Score 7 or Higher at Day 14 With Adjustment for the Parsimonious Covariate Set The curves with dashed lines indicate the prior distributions. The stopping rules were probability greater than 0.95 of an OR less than 1 and probability greater than 0.5 of an OR less than 0.8 for both outcomes; these are indicated with vertical dashed lines, respectively. Meta-analysis forest plots show bayesian estimates of the median ORs with their 95% credible intervals (CrIs) for individual randomized clinical trials^11,12,13,14,15,16,17,18^ and for the pooled OR.

The models for the 2 primary outcomes at day 14, adjusted for the parsimonious covariate set, indicated that the posterior median of the cumulative OR was 0.94 (95% CrI, 0.74-1.19), with posterior probability for OR less than 1 of 71%; the posterior median of the binary OR for WHO score of 7 or higher was 0.94 (95% CrI, 0.69-1.30), with posterior probability for OR less than 1 of 65%. Figure 3 shows the posterior distribution plots for the ORs of both primary outcomes at day 14; RCT-specific OR estimates indicate consistency. The prespecified stopping rules were not met.

The main effect estimates of the parsimonious covariates in the models for the cumulative and logistic ORs are shown in eFigure 4 in the Supplement. The largest effect sizes were observed for WHO score at baseline, age, and quarter of enrollment.

### Secondary and Tertiary Outcomes

Modeling the primary outcomes at day 14 adjusted for the expanded set of covariates showed similar results (eFigure 5 in the Supplement). The CONSORT diagram (eFigure 3 in the Supplement) shows the number of patients for the analysis of the secondary outcomes at day 28. Figure 1 gives the distributions of the WHO scores by treatment. At day 28, 188 participants (16.7%) in the control group and 178 (14.7%) participants in the CCP group had a WHO score of 7 to 10. Figure 2 presents a waterfall plot of the change from baseline by treatment.

The models for the WHO score and the indicator for WHO score of 7 or higher at day 28, adjusted for the expanded covariate set (eFigure 6 in the Supplement), indicated the median of the cumulative OR for the ordinal WHO was 0.94 (95% CrI, 0.74-1.19), with posterior probability of OR less than 1 of 72%, and the median of the OR for WHO score of 7 or higher was 0.91 (95% CrI, 0.67-1.24), with posterior probability for OR less than 1 of 74%. eFigure 6 in the Supplement shows the posterior distribution plots and the respective ORs (with 95% CrIs) overall and by RCT.

eFigure 7 in the Supplement gives Kaplan-Meier curves for time to death and cumulative incidence curves for time to discharge. The unadjusted mortality through day 14 was 8.6% in the control group and 6.7% in the CCP group; by day 28, the mortality rates were 13.6% and 10.9%, respectively (stratified log-rank test χ^2^ = 2.8; *P* = .09). The estimated mean postdischarge days through day 28 were 16.7 in the control group and 17.5 in the CCP group for a between-group difference of 0.84 day (95% CI, 0.22-1.62 days; Gray test χ^2^ = 3.92; *P* = .048).

The bayesian models for all-cause mortality at days 14 and 28, with expanded adjustment for covariates, indicated that at day 14, the median OR was 0.88 (95% CrI, 0.61-1.26) with posterior probability of OR less than 1 of 77%, and at day 28, the median OR was 0.85 (95% CrI, 0.62-1.18) with probability of OR less than 1 of 84%. eFigure 8 in the Supplement shows the posterior distributions of the mortality ORs overall and by RCT.

The estimated RCT-specific ORs shown in Figure 3 and in eFigure 5 and eFigure 6 in the Supplement indicated that CCP effect sizes were consistent. The main effect sizes of most covariates (age and baseline WHO score) were also consistent across outcomes and timing of assessment, whereas the effect size of quarter of enrollment exhibited some variability (eFigure 9, eFigure 10, and eFigure 11in the Supplement).

### Heterogeneity of Treatment Effect Sizes by Patient Characteristics

Results from exploratory analyses based on the models for the primary and secondary outcomes are shown in eFigure 12, eFigure 13, eFigure 14, eFigure 15, eFigure 16, and eFigure 17 in the Supplement. They showed substantial heterogeneity of treatment effect sizes and suggested that CCP was more than minimally associated with benefit in some subgroups, including those with baseline WHO score of 4, blood type A, and preexisting diabetes, cardiovascular, and pulmonary disease. The effect sizes were similar across age groups (≤50, 50-65, and >60 years) and did not vary consistently with duration of symptoms before treatment. eTable 3 and eTable 4 in the Supplement show the distributions of the ORs for the ordinal WHO scores at day 14 and day 28, respectively, in subgroups defined by baseline covariates. eTable 5 in the Supplement gives a summary of all modeling results.

### Sensitivity Analyses

The prespecified sensitivity analyses (eAppendix 1 in the Supplement) were directionally and substantively consistent with all results described here; results are in eTable 6, eTable 7, eTable 8, and eFigure 18 in the Supplement. We did not observe variation in treatment effect sizes by type of control condition.

## Discussion

This prospective IPD meta-analysis of international RCTs of CCP for hospitalized, noncritically ill patients with COVID-19 provides insights about CCP therapy. We found that CCP was associated with neither benefit nor harm consistently across RCTs. The estimated treatment effect size varied depending on the outcome, timing of its assessment, and stage of the pandemic. We observed heterogeneity of the treatment effect size, with evidence for more than minimal CCP association with clinical outcomes for some patient subgroups (eg, WHO score of 4 at baseline, preexisting diabetes and/or cardiovascular disease, and blood type A).

When the RCTs were launching in 2020, there was uncertainty about metrics for judging the efficacy and safety of CCP, including outcomes and timing of assessment. Our findings of the association of CCP with outcomes and the heterogeneity of the treatment effect size are robust: they were directionally consistent, across both the 8 RCTs and an array of prespecified end points, and our sensitivity analyses supported the findings. One RCT^45^ has suggested potential benefit of CCP in elderly outpatients within the first 72 hours of disease onset. Evidence supporting monoclonal antibody–based therapy is now available, but only for outpatients shortly after disease onset.^46,47^ Therefore, the lack of a clear effect of CCP even in patients with recent symptom onset is somewhat surprising and suggests that the window of opportunity for antibody-based therapy may be narrow and associated more with stage of illness than with precise timing.

CCP is a resource requiring individuals to donate plasma and infrastructure to obtain, process, and vet donated units for safety and the presence of SARS-CoV-2 antibody. The clinical and medical community urgently needed information on its safety and potential benefit. Although pooling IPD is not novel, it is typically undertaken only with completed and published RCTs. The COMPILE program was designed to accelerate the evaluation of CCP and grappled with the challenges of pooling IPD from different populations, a variety of health care systems, and 3 different control treatments in the context of evolving treatment strategies and emerging variants of SARS-CoV-2. The RCTs spanned the pandemic from April 2020 through March 2021. A majority of the RCTs were conducted over portions of this period, with only 1 spanning the entire interval. COMPILE addressed pandemic trends by adjusting for enrollment quarter in all analyses. Compared with the first quarter (April to June 2020), better outcomes were observed in later quarters.

These considerations necessitated a flexible monitoring system without statistical penalties for frequent inspections of data. COMPILE provides a practical solution that can offer critical information to regulatory authorities and the clinical community and overcomes the inherent difficulties of rapidly initiating large trials with multiple enrolling sites. COMPILE’s approach was helped by the ability to observe outcomes quickly, 2 and 4 weeks, rather than months or years. Another key strength was the timely development and widespread adoption of the COVID-19 clinical status scale.^34^ Our methods for COMPILE can be used beyond pandemic circumstances and are ideal for settings where a clinical response is rapidly available, as with many infectious diseases.

COMPILE differs from conventional approaches. Conventional meta-analyses pool data from trials after completion, providing a summary of evidence but having no ability to guide the trials while they are ongoing. A recent development was real-time meta-analysis of trial-level summary information,^10,48^ but those efforts did not incorporate IPD. COMPILE used a novel, powerful, model-based analysis that uniquely synthesized the comprehensive information provided by IPD from each RCT to enhance generalizability and provide a perspective on the therapeutic potential of CCP. This overcomes some of the limitations of large pragmatic trials, which may enroll a substantial number of participants with less diverse characteristics, limiting external validity.

 The COMPILE program assembled high-quality data from 8 RCTs of CCP in hospitalized patients with COVID-19 not requiring mechanical ventilation, analyzed with a robust bayesian approach. As with other COVID-19 therapies, CCP was not associated with benefit for the typical patient. There was heterogeneity of effect sizes with respect to baseline WHO score, blood type, history of diabetes, history of cardiovascular disease, and quarter of enrollment. Those observations, combined with the increased recent interest in the potential of precision medicine,^49,50^ led to the development of a treatment benefit index.^44^

### Limitations

This study has limitations that should be addressed. The evolving treatment of COVID-19, in combination with the emergence of SARS-CoV-2 variants, may have decreased the study’s overall power to assess CCP. Our model-based approach necessitates careful assessment of modeling choices, particularly the prior distributions. We conducted comprehensive simulations to assess sensitivity to modeling assumptions and extensive sensitivity analyses of the RCT data and found a high degree of robustness in our conclusions. Not all collaborating RCTs systematically collected concomitant medications at randomization, preventing evaluation of their impact. The RCTs that evaluated patients’ own SARS-CoV-2 antibodies before treatment used different measures, precluding exploration of this potentially important feature. Assessment of CCP antibody titers was also variable.^51^ The choice of stopping rules was also potentially influential. We chose rules with a strong basis in clinical decision-making, and the cDSMB agreed on them in advance. The bayesian monitoring framework may be less familiar to many researchers and may seem at odds with more traditional frequentist group-sequential monitoring approaches, in which control of type I error is critical. Of note, decisions in a bayesian framework are made not through hypothesis tests, but through characterization of uncertainty in terms of posterior probability, providing an easily interpretable, clinically relevant summary of the accruing information.

## Conclusions

Although we found no association between CCP and clinical outcomes, the study itself is notable for its differences from a traditional meta-analysis. COMPILE provided comprehensive results through an international collaboration, sparked by the urgency of the COVID-19 pandemic; the methods, however, apply broadly outside of crisis circumstances. The COMPILE project required technical infrastructure, advanced statistical modeling techniques, and the will to join forces. The data set will be available as a rich resource to support future work.

## Notes

Supplement. **eFigure 1.** WHO 11-Point COVID-19 Clinical Status Scale **eTable 1.** Prespecified Analyses **eFigure 2.** Ring Diagram of Patients in COMPILE **eTable 2.** RCT-Specific Baseline Characteristics **eFigure 3.** CONSORT Diagram **eFigure 4.** Covariate Effects for Primary Outcomes at Day 14 (Ordinal WHO Scores and Binary WHO ≥7), Parsimonious **eFigure 5.** Primary Outcome Results at Day 14 With Expanded Covariate Adjustment **eFigure 6.** Secondary Outcome Results at Day 28 With Expanded Covariate Adjustment **eFigure 7.** Mortality and Time to Discharge **eFigure 8.** Posterior Distributions for Mortality at Day 14 and Day 28 **eFigure 9.** Covariate Effects for Primary Outcomes at Day 14, Expanded **eFigure 10.** Covariate Effects for Secondary Outcomes at Day 28, Expanded **eFigure 11.** Covariate Effects for Mortality at Day 14 and Day 28, Expanded **eFigure 12.** Heterogeneity of Treatment Effect, Ordinal WHO Score at Day 14 **eFigure 13.** Heterogeneity of Treatment Effect, WHO ≥ 7 at Day 14 **eFigure 14.** Heterogeneity of Treatment Effect, Mortality at Day 14 **eFigure 15.** Heterogeneity of Treatment Effect, Ordinal WHO Score at Day 28 **eFigure 16.** Heterogeneity of Treatment Effect, WHO ≥ 7 at Day 28 **eFigure 17.** Heterogeneity of Treatment Effect, Mortality at Day 28 **eTable 3.** Heterogeneity of Treatment Effect: Summary of Outcomes at Day 14 **eTable 4.** Heterogeneity of Treatment Effect: Summary of Outcomes at Day 28 **eTable 5.** Summary of Results **eTable 6.** Summary of Results With Weakly Informative Prior **eTable 7.** Summary of Results With Hypothetical Influential Prior **eTable 8.** Summary of Results With Multiple Imputation **eFigure 18.** Cochrane RoB Tool Results **eAppendix 1.** Supplemental Statistical Information **eAppendix 2.** Committee Rosters **eAppendix 3.** Governance Documents **eAppendix 4.** RCT-Specific Information
