# Error in Figure 3

**DOI:** 10.1001/jamanetworkopen.2022.4556

**Published:** 2022-03-04

In the Original Investigation titled “Association of Convalescent Plasma Treatment With Clinical Status in Patients Hospitalized With COVID-19: A Meta-analysis,”^1^ published January 25, 2022, there were errors in Figure 3. The figure labels indicating posterior probabilities of an odds ratio less than .08 should have used “0.8” instead. This article has been corrected.^1^
