# Supplementary material for: Association of Convalescent Plasma Treatment With Clinical Status in Patients Hospitalized With COVID-19: A Meta-analysis
Source: JAMA Netw Open. 2022 Jan 25;5(1):e2147331. doi: 10.1001/jamanetworkopen.2021.47331 (PMC8790669; doi:10.1001/jamanetworkopen.2021.47331)
Supplement: Supplement. — eFigure 1. WHO 11-Point COVID-19 Clinical Status Scale eTable 1. Prespecified Analyses eFigure 2. Ring Diagram of Patients in COMPILE eTable 2. RCT-Specific Baseline Characteristics eFigure 3. CONSORT Diagram eFigure 4. Covariate Effects for Primary Outcomes at Day 14 (Ordinal WHO Scores and Binary WHO ≥7), Parsimonious eFigure 5. Primary Outcome Results at Day 14 With Expanded Covariate Adjustment eFigure 6. Secondary Outcome Results at Day 28 With Expanded Covariate Adjustment eFigure 7. Mortality and Time to Discharge eFigure 8. Posterior Distributions for Mortality at Day 14 and Day 28 eFigure 9. Covariate Effects for Primary Outcomes at Day 14, Expanded eFigure 10. Covariate Effects for Secondary Outcomes at Day 28, Expanded eFigure 11. Covariate Effects for Mortality at Day 14 and Day 28, Expanded eFigure 12. Heterogeneity of Treatment Effect, Ordinal WHO Score at Day 14 eFigure 13. Heterogeneity of Treatment Effect, WHO ≥ 7 at Day 14 eFigure 14. Heterogeneity of Treatment Effect, Mortality at Day 14 eFigure 15. Heterogeneity of Treatment Effect, Ordinal WHO Score at Day 28 eFigure 16. Heterogeneity of Treatment Effect, WHO ≥ 7 at Day 28 eFigure 17. Heterogeneity of Treatment Effect, Mortality at Day 28 eTable 3. Heterogeneity of Treatment Effect: Summary of Outcomes at Day 14 eTable 4. Heterogeneity of Treatment Effect: Summary of Outcomes at Day 28 eTable 5. Summary of Results eTable 6. Summary of Results With Weakly Informative Prior eTable 7. Summary of Results With Hypothetical Influential Prior eTable 8. Summary of Results With Multiple Imputation eFigure 18. Cochrane RoB Tool Results eAppendix 1. Supplemental Statistical Information eAppendix 2. Committee Rosters eAppendix 3. Governance Documents eAppendix 4. RCT-Specific Information [file jamanetwopen-e2147331-s001.pdf]

Supplemental Online Content

Troxel AB, Petkova E, Goldfeld K, et al. Association of convalescent plasma treatment with clinical status in patients hospitalized with COVID-19: a meta-analysis. *JAMA Netw Open*. 2022;5(1):e2147331. doi:10.1001/jamanetworkopen.2021.47331

eFigure 1. WHO 11-Point COVID-19 Clinical Status Scale
eTable 1. Prespecified Analyses
eFigure 2. Ring Diagram of Patients in COMPILE
eTable 2. RCT-Specific Baseline Characteristics
eFigure 3. CONSORT Diagram
eFigure 4. Covariate Effects for Primary Outcomes at Day 14 (Ordinal WHO Scores and Binary WHO ≥7), Parsimonious
eFigure 5. Primary Outcome Results at Day 14 With Expanded Covariate Adjustment
eFigure 6. Secondary Outcome Results at Day 28 With Expanded Covariate Adjustment
eFigure 7. Mortality and Time to Discharge
eFigure 8. Posterior Distributions for Mortality at Day 14 and Day 28
eFigure 9. Covariate Effects for Primary Outcomes at Day 14, Expanded
eFigure 10. Covariate Effects for Secondary Outcomes at Day 28, Expanded
eFigure 11. Covariate Effects for Mortality at Day 14 and Day 28, Expanded
eFigure 12. Heterogeneity of Treatment Effect, Ordinal WHO Score at Day 14
eFigure 13. Heterogeneity of Treatment Effect, WHO ≥ 7 at Day 14
eFigure 14. Heterogeneity of Treatment Effect, Mortality at Day 14
eFigure 15. Heterogeneity of Treatment Effect, Ordinal WHO Score at Day 28
eFigure 16. Heterogeneity of Treatment Effect, WHO ≥ 7 at Day 28
eFigure 17. Heterogeneity of Treatment Effect, Mortality at Day 28
eTable 3. Heterogeneity of Treatment Effect: Summary of Outcomes at Day 14
eTable 4. Heterogeneity of Treatment Effect: Summary of Outcomes at Day 28
eTable 5. Summary of Results
eTable 6. Summary of Results With Weakly Informative Prior
eTable 7. Summary of Results With Hypothetical Influential Prior
eTable 8. Summary of Results With Multiple Imputation
eFigure 18. Cochrane RoB Tool Results
eAppendix 1. Supplemental Statistical Information
eAppendix 2. Committee Rosters
eAppendix 3. Governance Documents
eAppendix 4. RCT-Specific Information

This supplemental material has been provided by the authors to give readers additional information about their work.

© 2022 Troxel AB et al. *JAMA Network Open.*

eFigure 1. WHO 11-Point COVID-19 Clinical Status Scale

The WHO 11-point COVID-19 scale[1]. The brackets on the right define the outcomes assessed at days 14 and 28.

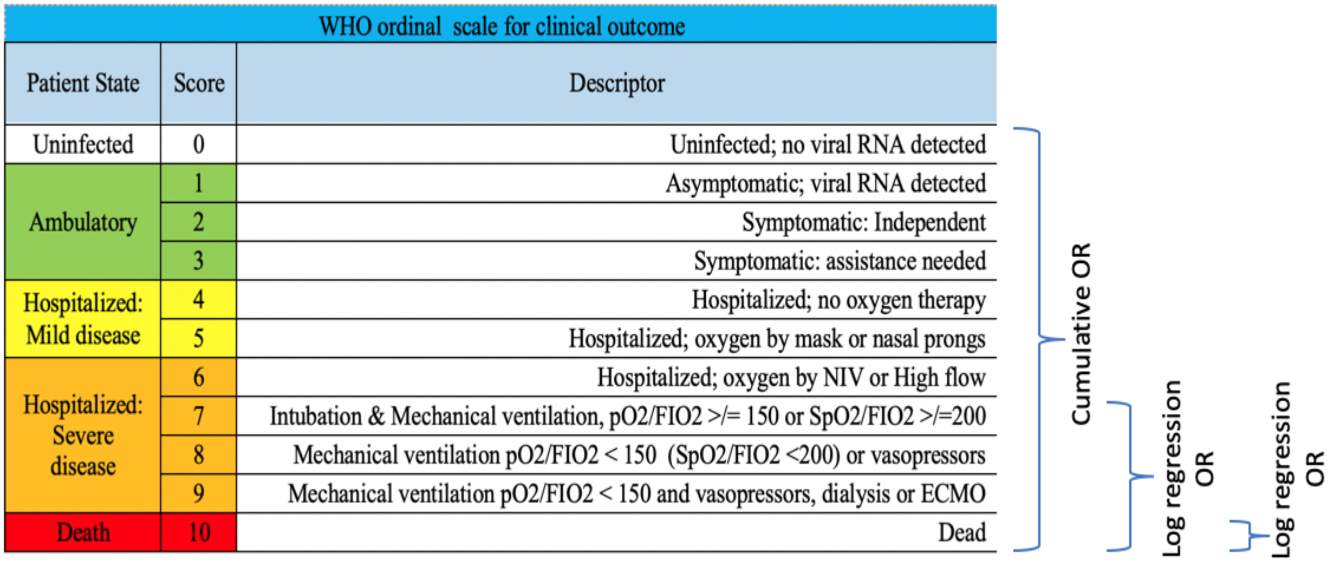

WHO ordinal scale for clinical outcome		
Patient State	Score	Descriptor
Uninfected	0	Uninfected; no viral RNA detected
Ambulatory	1	Asymptomatic; viral RNA detected
	2	Symptomatic: Independent
	3	Symptomatic: assistance needed
Hospitalized: Mild disease	4	Hospitalized; no oxygen therapy
	5	Hospitalized; oxygen by mask or nasal prongs
Hospitalized: Severe disease	6	Hospitalized; oxygen by NIV or High flow
	7	Intubation & Mechanical ventilation, pO2/FIO2 >/= 150 or SpO2/FIO2 >/=200
	8	Mechanical ventilation pO2/FIO2 < 150 (SpO2/FIO2 <200) or vasopressors
	9	Mechanical ventilation pO2/FIO2 < 150 and vasopressors, dialysis or ECMO
Death	10	Dead

© 2022 Troxel AB et al. *JAMA Network Open.*

eTable 1. Prespecified Analyses

Analysis #	Caegory	Description	Time since Randomization	Treatment	Covariate Adjustment	Interim Analysis Rule # 1	Interim Analysis Rule #2	Interactions			
								Sex	Age	Symp dur.	WHO base
1	**Primary**	CO[1] WHO score	D14	CCP vs Control(3)	Parsimonious[2]	Both primary endpoints are satisfied	At least one of the primary is satisfied and at least one of the secondary is satisfied	a	b	c	d
2	**Primary**	WHO 7-10[3]	D14	CCP vs Control(3)	Parsimonious			a	b	c	d
3	Secondary	CO WHO score	D14	CCP vs Control(3)	Expanded[4]			a	b	c	d
4	Secondary	WHO 7-10[3]	D14	CCP vs Control(3)	Expanded			a	b	c	d
5	Secondary	CO WHO score	D28	CCP vs Control(3)	Expanded			a	b	c	d
6	Secondary	WHO 7-10[3]	D28	CCP vs Control(3)	Expanded			a	b	c	d
7	Tertiary	Total Mortality[3]	D14	CCP vs Control(3)	Expanded			a	b	c	d
8	Tertiary	Total Mortality[3]	D28	CCP vs Control(3)	Expanded			a	b	c	d
9	Tertiary	Time to discharge and death[5]		CP vs Control(3)	Expanded			a	b	c	d
Effect of number of CP units (0, 1, 2, …) and levels of antibodies (Ab) in the CP (none, low, not-low)											
10	Tertiary	CO WHO score	D14	# CCP units/Ab level	Expanded			a	b	c	d
11	Tertiary	WHO 7-10[3]	D14	# CCP units/Ab level	expended			a	b	c	d
12	Tertiary	CO WHO score	D28	# CCP units/Ab level	Expanded			a	b	c	d
13	Tertiary	WHO 7-10[3]	D28	# CCP units/Ab level	Expanded			a	b	c	d
14	Tertiary	Total Mortality[3]	D14	# CCP units/Ab level	Expanded			a	b	c	d
15	Tertiary	Total Mortality[3]	D28	# CCP units/Ab level	Expanded			a	b	c	d
16	Tertiary	Time discharge and death		# CCP units/Ab level	Expanded			a	b	c	d

© 2022 Troxel AB et al. *JAMA Network Open.*

eFigure 2. Ring Diagram of Patients in COMPILE

The figure provides information of the COMPILE patients by RCT, site within RCT and type of control

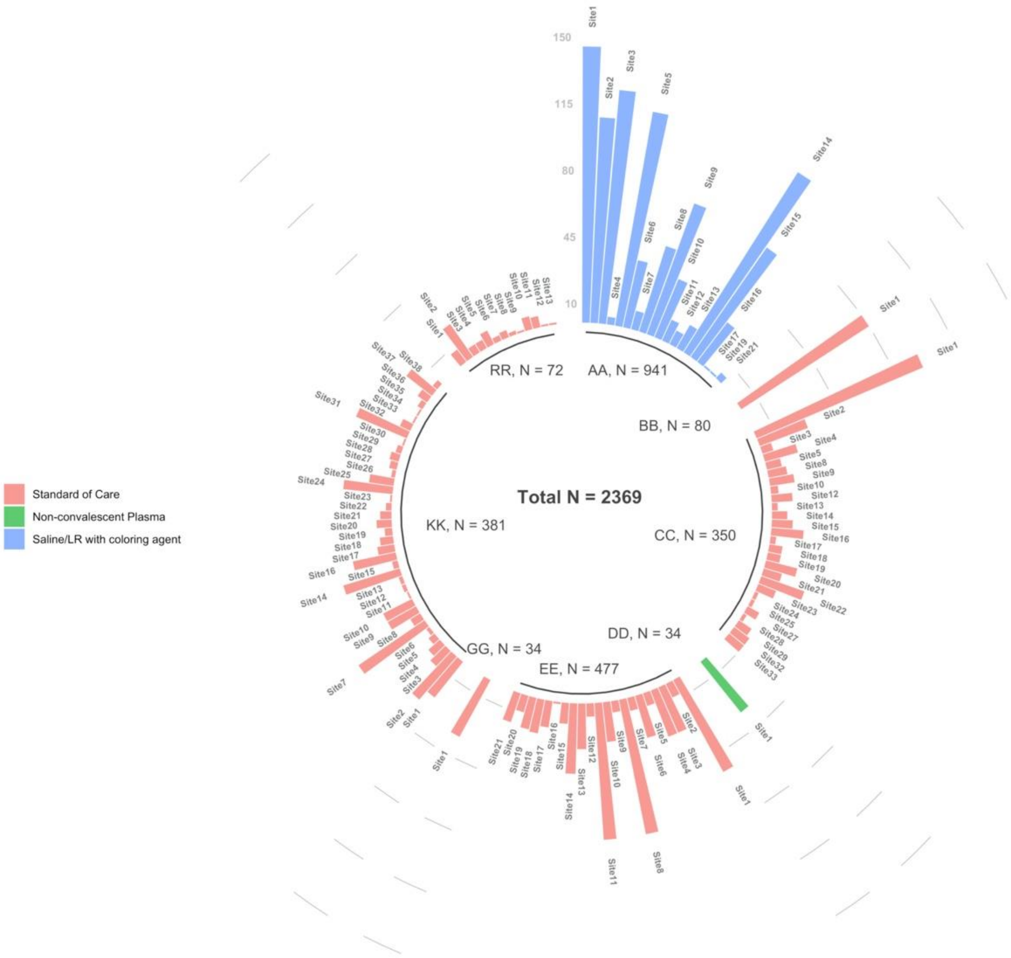

© 2022 Troxel AB et al. *JAMA Network Open.*

eTable 2. RCT-Specific Baseline Characteristics

	AA	BB	CC	DD	EE	GG	KK	RR
n	941	80	350	34	477	34	381	72
Age (median, IQR)	63.00 [52.00, [illegible]	62.50 [51.00, [illegible]	62.00 [53.25, [illegible]	56.00 [44.50, [illegible]	62.00 [52.00, [illegible]	53.50 [46.25, [illegible]	52.00 [42.00, [illegible]	64.00 [56.00, [illegible]
Sex = Female (n, %)	385 (40.9)	44 (55.0)	121 (34.6)	19 (55.9)	150 (31.4)	13 (38.2)	90 (23.6)	23 (31.9)
Baseline WHO [illegible]								
4 - hospitalized/ no [illegible]	0 (0.0)	4 (5.0)	74 (21.1)	2 (5.9)	54 (11.3)	7 (20.6)	303 (79.5)	8 (11.1)
5 - hospitalized/ O2 [illegible]	673 (71.5)	39 (48.8)	276 (78.9)	7 (20.6)	350 (73.4)	26 (76.5)	77 (20.2)	53 (73.6)
6 - hospitalized/ O2	268 (28.5)	37 (46.2)	0 (0.0)	25 (73.5)	73 (15.3)	1 (2.9)	1 (0.3)	11 (15.3)
Blood group (n, %)								
O	489 (52.0)	44 (55.0)	156 (44.6)	16 (47.1)	208 (43.6)	15 (44.1)	134 (35.2)	24 (33.3)
A	274 (29.1)	28 (35.0)	149 (42.6)	8 (23.5)	211 (44.2)	11 (32.4)	91 (23.9)	22 (30.6)
B	135 (14.3)	8 (10.0)	31 (8.9)	7 (20.6)	46 (9.6)	4 (11.8)	135 (35.4)	10 (13.9)
AB	41 (4.4)	0 (0.0)	9 (2.6)	3 (8.8)	12 (2.5)	4 (11.8)	21 (5.5)	2 (2.8)
Not available	2 (0.2)	0 (0.0)	5 (1.4)	0 (0.0)	0 (0.0)	0 (0.0)	0 (0.0)	14 (19.4)
Days since symptoms [illegible]								
0-3 days	153 (16.3)	17 (21.2)	48 (13.7)	3 (8.8)	38 (8.0)	1 (2.9)	24 (6.3)	6 (8.3)
4-6 days	310 (32.9)	24 (30.0)	213 (60.9)	8 (23.5)	156 (32.7)	7 (20.6)	102 (26.8)	15 (20.8)
7-10 days	344 (36.6)	24 (30.0)	83 (23.7)	20 (58.8)	173 (36.3)	26 (76.5)	144 (37.8)	19 (26.4)
11-14 days	80 (8.5)	13 (16.2)	6 (1.7)	3 (8.8)	64 (13.4)	0 (0.0)	82 (21.5)	13 (18.1)
>14 days	53 (5.6)	2 (2.5)	0 (0.0)	0 (0.0)	30 (6.3)	0 (0.0)	29 (7.6)	18 (25.0)
Not available	1 (0.1)	0 (0.0)	0 (0.0)	0 (0.0)	16 (3.4)	0 (0.0)	0 (0.0)	1 (1.4)
Days since COVID-19 diagnosis at	1.00 [1.00, 2.00]	2.00 [1.00, 4.00]	2.00 [1.00, 4.00]	2.00 [1.00, 4.00]	1.00 [1.00, 3.00]	4.00 [2.00, 6.00]	NA [NA, NA]	NA [NA, NA]
History of diabetes = [illegible]	332 (35.3)	32 (40.0)	90 (25.7)	10 (29.4)	140 (29.4)	9 (26.5)	163 (42.8)	19 (26.4)
History of pulmonary [illegible]								
No	844 (89.7)	57 (71.2)	293 (83.7)	21 (61.8)	437 (91.6)	34 (100.0)	343 (90.0)	51 (70.8)
Yes	97 (10.3)	23 (28.7)	57 (16.3)	13 (38.2)	31 (6.5)	0 (0.0)	38 (10.0)	21 (29.2)
Not available	0 (0.0)	0 (0.0)	0 (0.0)	0 (0.0)	9 (1.9)	0 (0.0)	0 (0.0)	0 (0.0)
History of [illegible]								
No	525 (55.8)	21 (26.2)	169 (48.3)	30 (88.2)	191 (40.0)	18 (52.9)	358 (94.0)	42 (58.3)
Yes	416 (44.2)	59 (73.8)	181 (51.7)	4 (11.8)	279 (58.5)	16 (47.1)	23 (6.0)	30 (41.7)
Not available	0 (0.0)	0 (0.0)	0 (0.0)	0 (0.0)	7 (1.5)	0 (0.0)	0 (0.0)	0 (0.0)
Enrollment quarter (n, [illegible]								
Apr-June [illegible]	170 (18.1)	19 (23.8)	77 (22.0)	4 (11.8)	20 (4.2)	6 (17.6)	273 (71.7)	72 (100.0)
July-Sept [illegible]	113 (12.0)	22 (27.5)	67 (19.1)	30 (88.2)	98 (20.5)	19 (55.9)	108 (28.3)	0 (0.0)
Oct-Dec 2020	407 (43.3)	32 (40.0)	136 (38.9)	0 (0.0)	325 (68.1)	9 (26.5)	0 (0.0)	0 (0.0)
Jan-Mar 2021	251 (26.7)	7 (8.8)	70 (20.0)	0 (0.0)	34 (7.1)	0 (0.0)	0 (0.0)	0 (0.0)

Figure 3. CONSORT Diagram

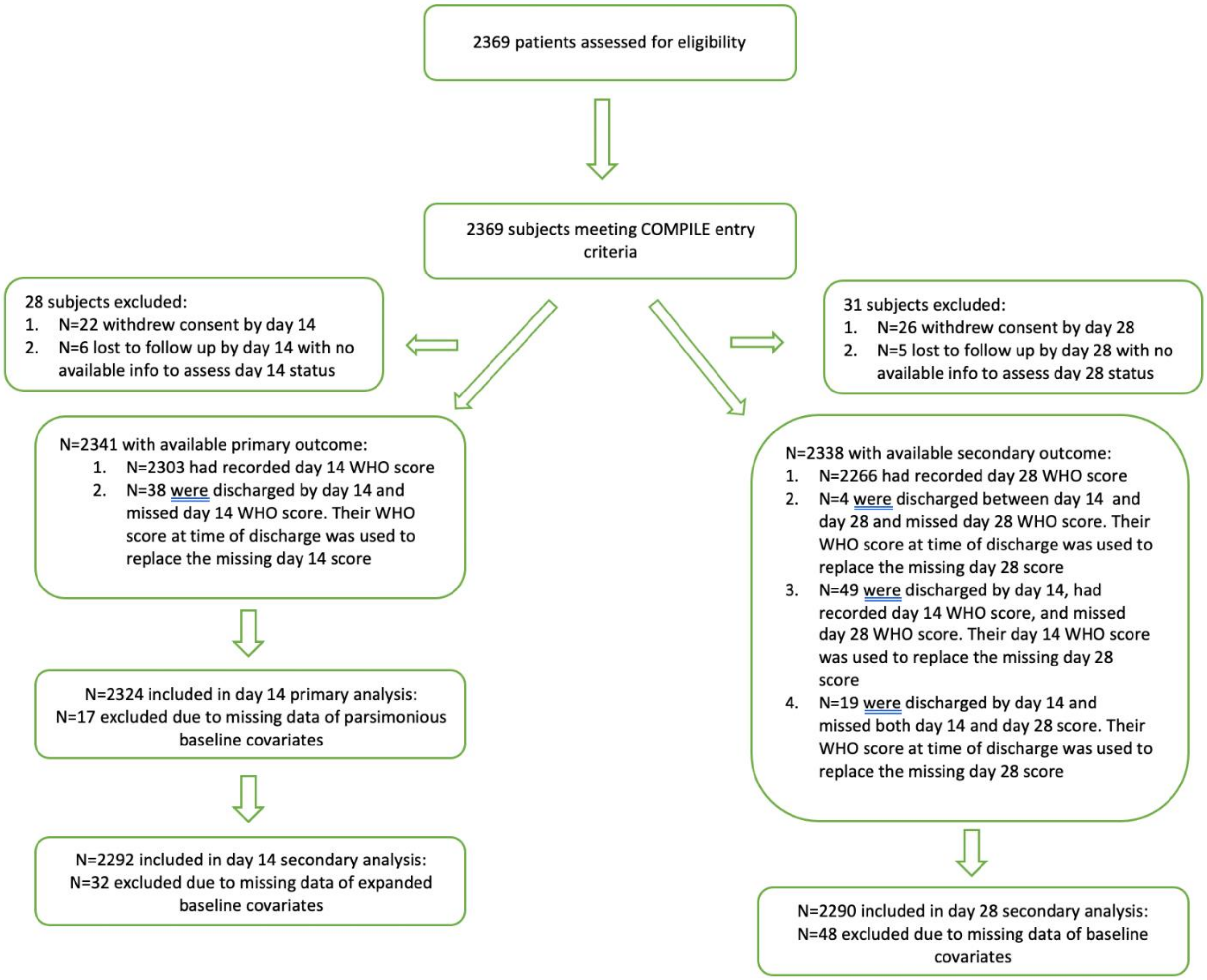

© 2022 Troxel AB et al. *JAMA Network Open.*

eFigure 4. Covariate Effects for Primary Outcomes at Day 14 (Ordinal WHO Scores and Binary WHO ≥7), Parsimonious

The figure below shows the main effects (regardless of treatment) of the covariates on the primary outcomes from the parsimonious adjustment model.

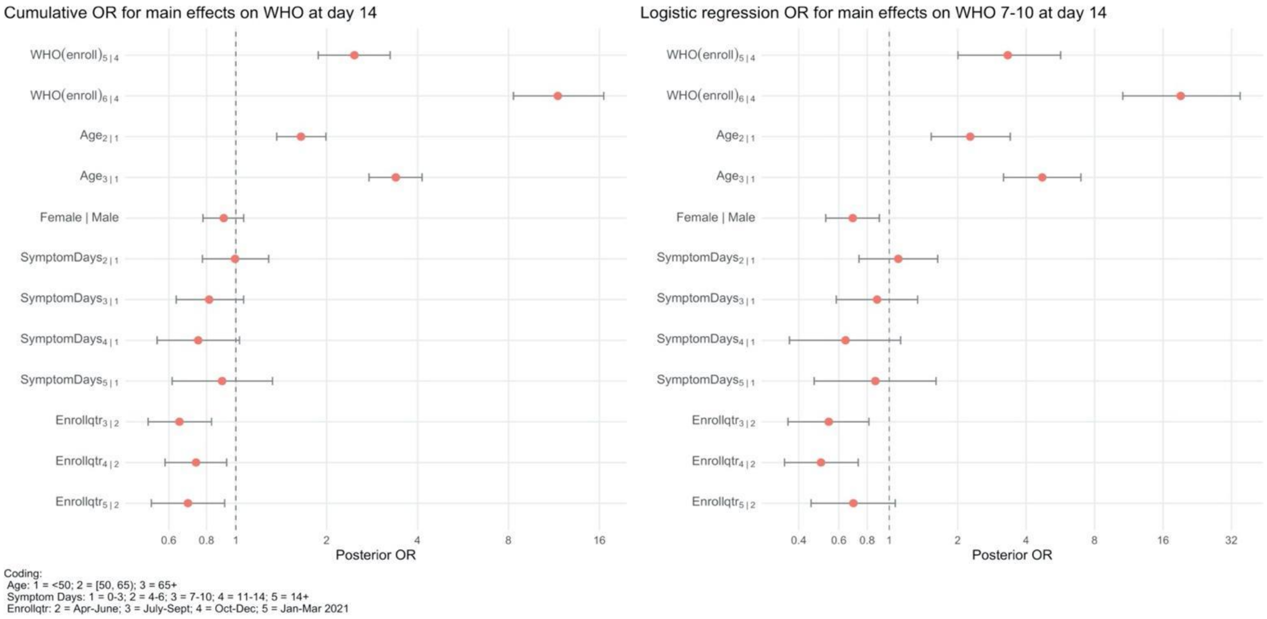

The main effect estimate of the stage of COVID-19 at baseline (measured by baseline WHO score) was by far the largest. In reference to patients with WHO=4 at baseline, those with WHO=5 had 2.8 times higher odds of a worse outcome on the ordinal WHO scale and 3.5 times higher odds of being on ventilator or worse at day 14. Patients with baseline WHO=6 had even worse odds for those undesirable outcomes: compared to patients with WHO=4 at baseline, those with WHO=6 had 12 times higher odds of a worse outcome on the ordinal WHO scale and 20 times higher odds of being on ventilator or worse at day 14. Of note here is that, controlling for baseline WHO score, days since symptom onset (in bins of 3 or 4 days) had a much smaller effect estimate than baseline disease status. In addition, the ordinal categorical days since symptoms factors did not show a monotonic relationship with the outcome.

The next largest main effect estimate was observed for age. In comparison with the reference category of age less than or equal to 50, patients aged 51 to 65 had 1.5 times higher odds of a worse score on the WHO scale and 2.2 times higher odds of being on ventilator or worse at day 14. Patients older than age 65 had even worse odds of an undesirable outcome: compared to patients age 50 or less, those patients had 3.5 times higher odds of a worse WHO scores and 4.3 times higher odds of being on ventilator or worse at day 14.

Interesting here is the effect estimate of quarter of enrollment. In comparison with the first quarter of the global pandemic (March through June 2021, here a reference quarter), the odds of an undesirable outcome were lower in all consecutive quarters. The reduction in the odds ranged from 0.4 to 0.8 for the ordinal WHO score. The reduction in odds of being on mechanical ventilation or worse was between 0.5 and 0.8. Interestingly, there was no clear trend of increased improvement over later quarters. This might be due to the appearance of new variants at the end of 2020 and the

© 2022 Troxel AB et al. *JAMA Network Open.*

beginning of 2021. The effect estimate of sex was small in magnitude, with females having lower odds of worse outcomes on average than males.

© 2022 Troxel AB et al. *JAMA Network Open.*

eFigure 5. Primary Outcome Results at Day 14 With Expanded Covariate Adjustment

Secondary analysis for the primary outcomes (the ordinal WHO score at day 28 and WHO ≥7 at day 28) included models with an adjustment for an expanded list of covariates, using cumulative proportional odds models and logistic regression, respectively. The figure below shows the posterior distributions of the odds ratios comparing CCP to control. The figure also shows a forest plot with the RCT-specific effects with the 95% credible intervals for the RCT-specific ORs.

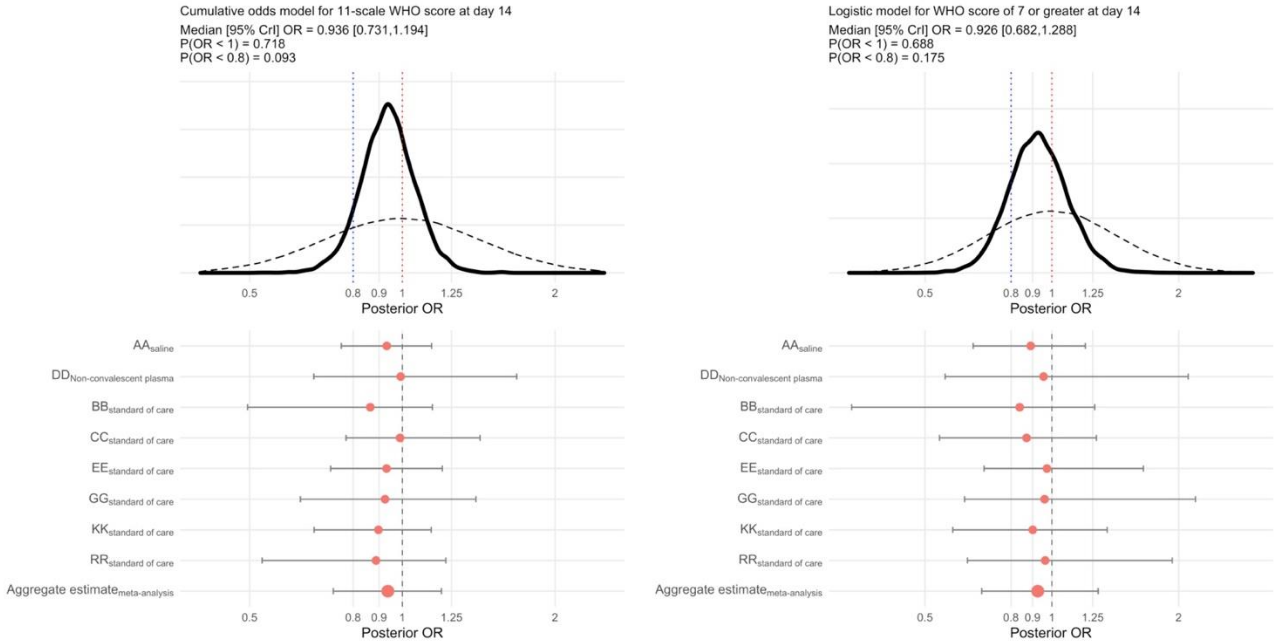

Odds ratios less than 1 (OR < 1) indicate any association of CCP with better outcomes. The posterior probability P(OR<1) indicates the likelihood that there is any CCP association, and P(OR <1) ≥ 90% can be viewed as strong evidence. Odds ratios less than 0.8 (OR<0.8) indicate more than minimal association of CCP with better outcomes. The posterior probability P(OR<0.8) indicates the likelihood that CCP is more than minimally associated. Jointly P(OR<1)≥90% and P(OR< 0.8) ≥ 50% can be viewed as substantial evidence that CCP is more than minimally associated with benefit. These posterior probabilities are shown in the top legends of the left and right panel of the figure above.

The OR measuring association of CCP with the ordinal WHO score at day 14 (left panel), with adjustment for the full set of covariates, is OR=0.94, with 95% credible interval (0.7, 1.19). The posterior probability any CCP association on average is P(OR<1)=72%, indicating weak evidence. The posterior probability for more than minimal association is P(OR<0.8)=9%, indicating lack of evidence for more than minimal association on average across all subjects. The RCT-specific CCP effect sizes were all consistent with respect to both magnitude and direction; see the bottom half of the two panels.

Similar results are observed with respect to the binary WHO≥7 outcome at day 14 (right panel). The overall average OR=0.93 with 95% credible interval (0.69, 1.18). The posterior probability of any association was P(OR<1)=69% indicating weak evidence. The posterior probability of more than minimal association was P(OR<0.8)=18%, indicating lack of evidence for more than minimal CCP association.

eFigure 6. Secondary Outcome Results at Day 28 With Expanded Covariate Adjustment

Secondary outcomes are the ordinal WHO score at day 28 and WHO ≥7 at day 28. These are analyzed using cumulative proportional odds models and logistic regression, respectively. Both models adjust for the expanded list of covariates. The figure below shows the posterior distributions of the odds ratios estimating the association of CCP with outcomes. The figure also shows a forest plot with the RCT-specific effect sizes with the 95% credible intervals for the RCT-specific ORs.

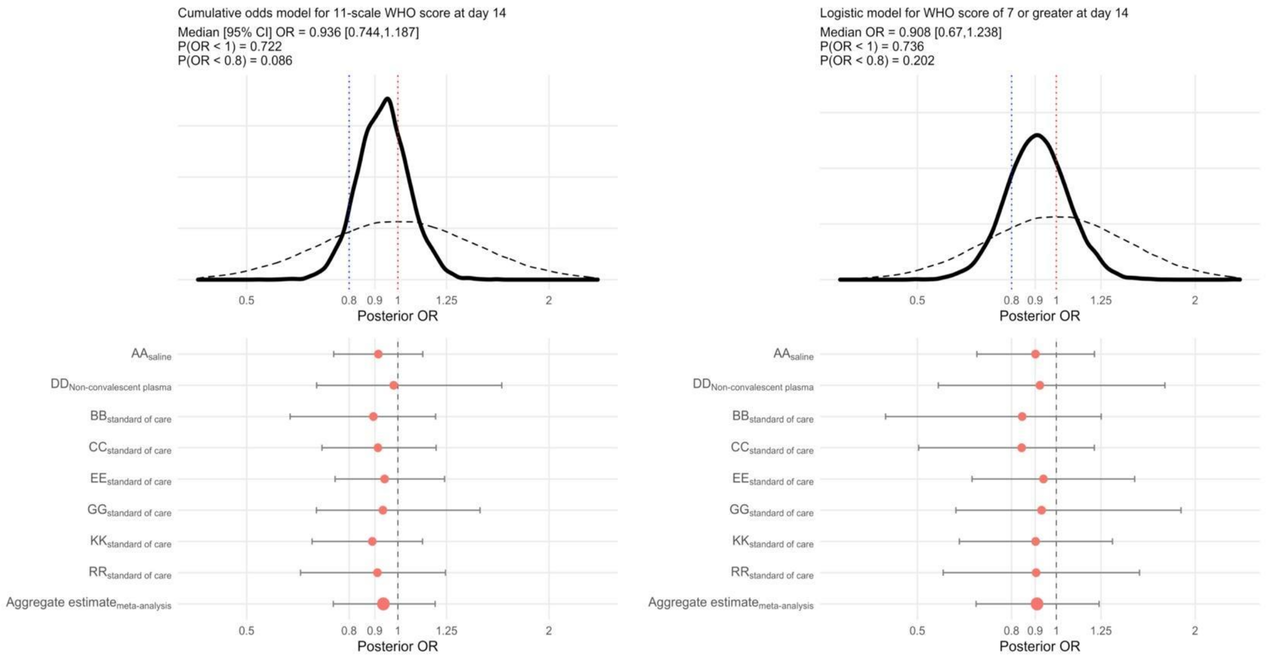

Odds ratios less than 1 (OR < 1) indicate any association of CCP with better outcomes. The posterior probability P(OR<1) indicates the likelihood that there is any CCP association, and P(OR <1) ≥ 90% can be viewed as strong evidence. Odds ratios less than 0.8 (OR<0.8) indicate more than minimal association of CCP with better outcomes. The posterior probability P(OR<0.8) indicates the likelihood that CCP is more than minimally associated. Jointly P(OR<1)≥90% and P(OR< 0.8) ≥ 50% can be viewed as substantial evidence that CCP is more than minimally associated with benefit. These posterior probabilities are shown in the top legends of the left and right panel of the figure above.

The OR measuring association of CCP with the ordinal WHO score at day 28 (left panel), with adjustment for the full set of covariates, is OR=0.94, with 95% credible interval (0.74, 1.19). The posterior probability any CCP association on average is P(OR<1)=72%, indicating weak evidence. The posterior probability for more than minimal association is P(OR<0.8)=9%, indicating lack of evidence for more than minimal association on average across all subjects. The RCT-specific CCP effect sizes were all consistent with respect to both magnitude and direction; see the bottom half of the two panels.

© 2022 Troxel AB et al. *JAMA Network Open.*

eFigure 7. Mortality and Time to Discharge

The figure below shows Kaplan-Meier plots for time to death (top two curves) and cumulative incidence plots for time to discharge (bottom two curves). To compare the treatment groups, stratified long-rank test is used for time to death and Gray's competing risk test is used for time to discharge from the hospital.

The figure shows some evidence for association of CCP with mortality, although not significant by the conventional guidelines (p-value= 0.09). There is also evidence for association of CCP with time to hospital discharge (p-value=0.05), indicating 0.8 days shorter stay for patients treated with CCP compared to those treated with control. The estimated duration of post-recovery time within 28 days was 16.7 days post-randomization for control patients and 17.5 days for patients treated with CCP.

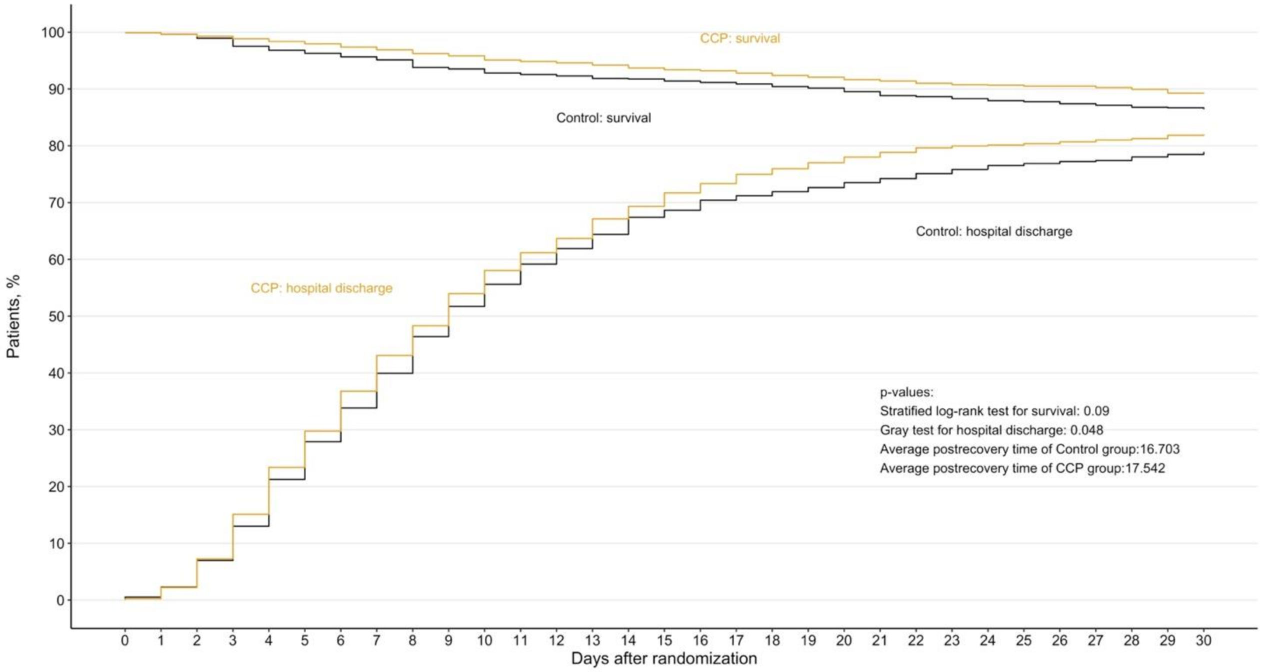

Survival

Control	1138	1131	1127	1117	1101	1093	1087	1080	1073	1058	1055	1047	1044	1041	1036	1034	1030	1027	1023	1017	1014	1007	999	997	993	988	986	982	979	975	974
CP	1231	1225	1220	1215	1209	1203	1198	1191	1185	1176	1171	1162	1159	1156	1151	1143	1138	1136	1131	1126	1122	1117	1114	1109	1106	1105	1103	1103	1100	1096	1087

Discharge

Control	1138	1125	1101	1038	954	853	772	698	623	535	472	420	377	343	310	275	257	234	222	209	199	182	168	156	144	132	126	118	114	103	97
CP	1231	1222	1193	1126	1024	917	834	741	658	586	513	455	414	380	334	301	268	246	221	204	187	170	157	142	135	132	127	123	116	110	97

© 2022 Troxel AB et al. *JAMA Network Open.*

eFigure 8. Posterior Distributions for Mortality at Day 14 and Day 28

The figure below shows the posterior distribution of the odds ratios estimating the association of CCP with mortality at day 14 (left panel) and day 28 (right panel). Bayesian logistic regression models with full covariate adjustment were used.

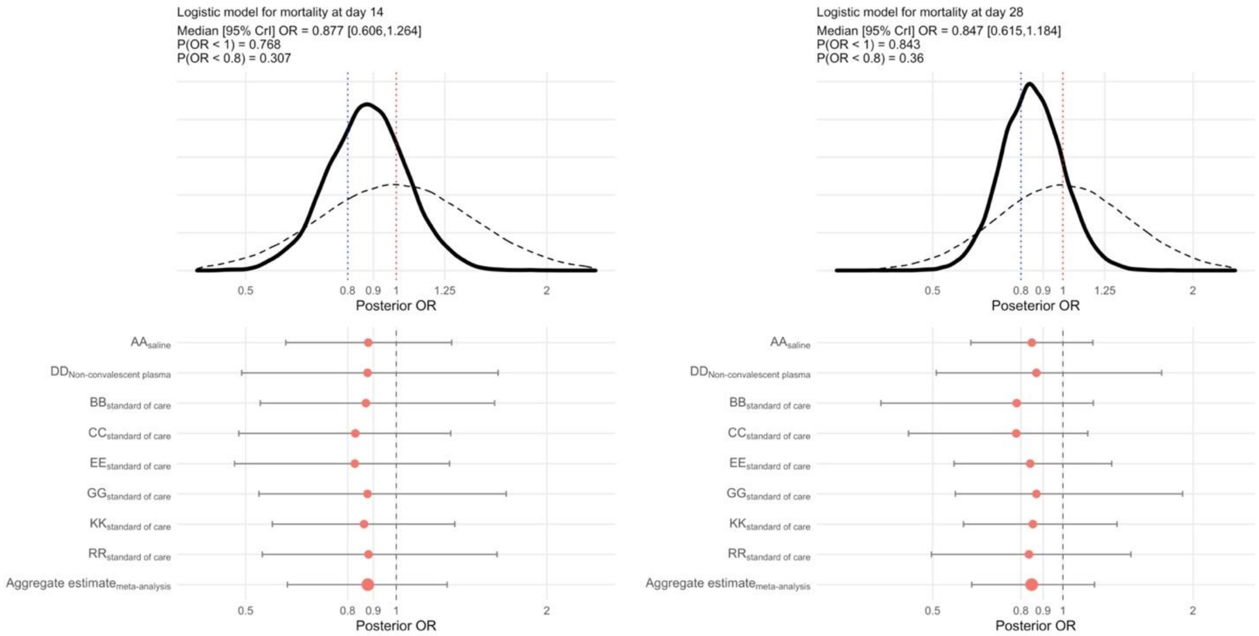

Odds ratios less than 1 (OR < 1) indicate any association of CCP with lower risk of mortality. The posterior probability P(OR<1) indicates the likelihood that there is any CCP association, and P(OR <1) ≥ 90% can be viewed as strong evidence. Odds ratios less than 0.8 (OR<0.8) indicate more than minimal association of CCP with lower mortality. The posterior probability P(OR<0.8) indicates the likelihood that CCP is more than minimally associated. Jointly P(OR<1)≥90% and P(OR< 0.8) ≥ 50% can be viewed as substantial evidence that CCP is more than minimally associated with lower mortality. These posterior probabilities are shown in the top legends of the left and right panel of the figure above.

The OR measuring association of CCP with mortality at day 14 (left panel), with adjustment for the full set of covariates, is OR=0.88, with 95% credible interval (0.61, 1.26). The posterior probability any CCP association on average is P(OR<1)=77%, indicating moderate evidence. The posterior probability for more than minimal association is P(OR<0.8)=31%, indicating weak evidence for more than minimal association on average across all subjects.

The OR measuring association of CCP with mortality at day 28 (right panel), with adjustment for the full set of covariates, is OR=0.85, with 95% credible interval (0.62, 1.18). The posterior probability any CCP association on average is P(OR<1)=84%, indicating substantial evidence. The posterior probability for more than minimal association is P(OR<0.8)=36%, indicating weak evidence for more than minimal association on average across all subjects.

The RCT-specific CCP effect sizes were all consistent with respect to both magnitude and direction; see the bottom half of the two panels.

© 2022 Troxel AB et al. *JAMA Network Open.*

eFigure 9. Covariate Effects for Primary Outcomes at Day 14, Expanded

Secondary analyses included the proportional odds models for the ordinal WHO score at day 14 and WHO≥7 at day 14, adjusting for the full set of covariates. The figure below gives the estimated main effect estimates of the covariates (regardless of treatment) in the models for the primary outcomes adjusted for the full set of covariates. In addition to the median of the posterior distributions of the ORs for the covariates' main effect estimates, 95% credible intervals are shown.

The main effect estimates of the covariates (regardless of treatment) observed from the primary models with parsimonious adjustment only are shown in eFigure 4. The findings regarding the overlapping covariates are similar to the analysis adjusting for the full set of covariates shown in the figure below. Note the evidence for a detrimental main effect of presence of medical complications (cardiovascular disease, diabetes, and pulmonary disease). Notable also is the negative association of blood types A and AB, compared to type O, at least with respect to the ordinal WHO score at day 14.

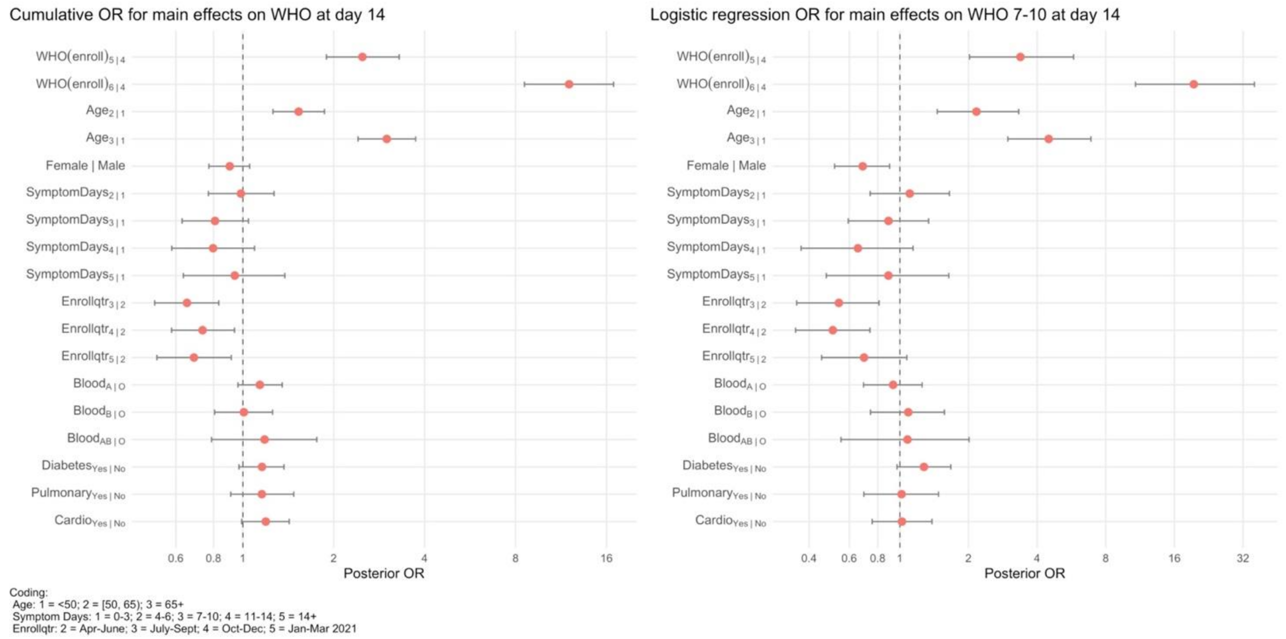

© 2022 Troxel AB et al. *JAMA Network Open.*

eFigure 10. Covariate Effects for Secondary Outcomes at Day 28, Expanded

The figure below shows the main effect estimates of the full set of covariates (regardless of treatment) of the covariates from models for the ordinal WHO scale (left panel) and the binary outcome of being on ventilator or worse (right panel) at day 28.

The main effect estimates for the outcomes at day 28 show a similar pattern to those in the models for outcomes at day 14; see Figure 4 in the main text and eFigure 9. Of note are the large associations with baseline disease status measured by baseline WHO score and age, observed also with respect to the outcomes at day 14. Similar to day 14, medical comorbidities and blood types A and AB show negative association with outcome regardless of treatment.

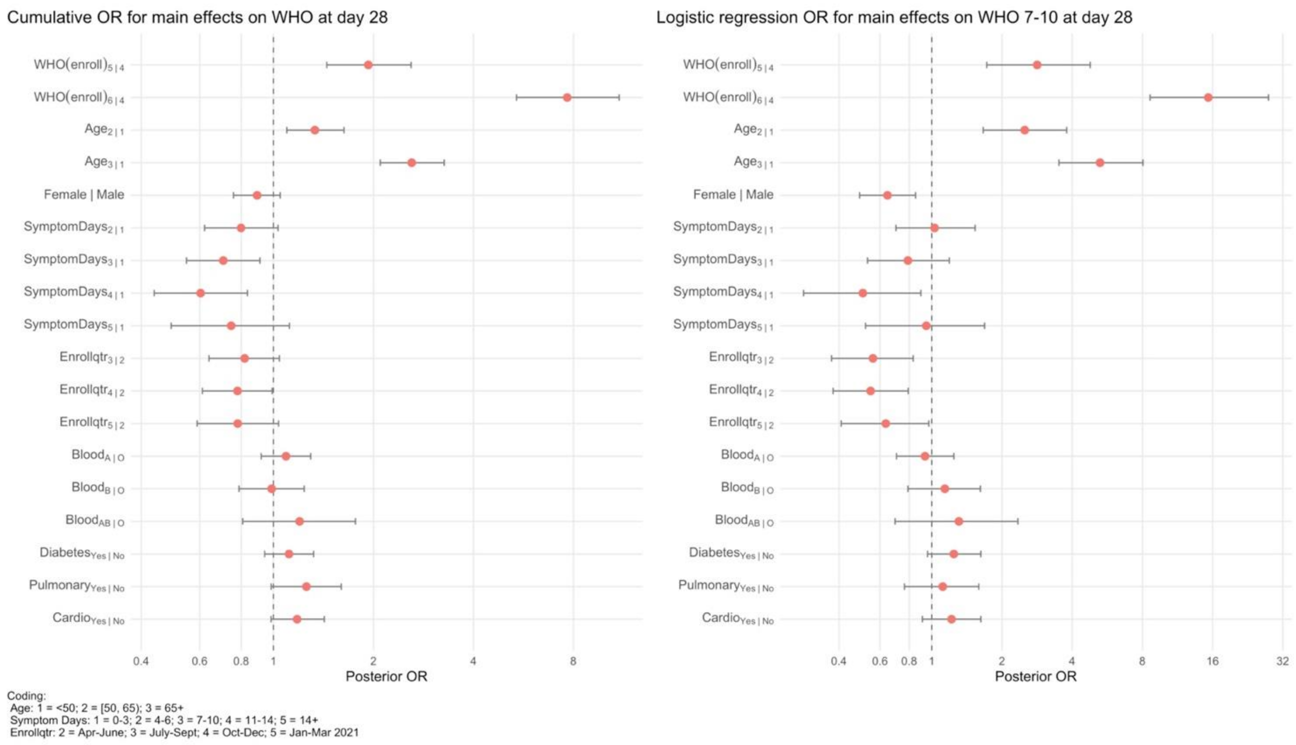

© 2022 Troxel AB et al. *JAMA Network Open.*

eFigure 11. Covariate Effects for Mortality at Day 14 and Day 28, Expanded

The figure below shows the main effect estimates of the full set of covariates (regardless of treatment) of the covariates from models for mortality at day 14 (left panel) and mortality at day 28 (right panel).

Interesting to note here is the strongly pronounced effect estimate of quarter on mortality at both day 14 and day 28. Compared with the first quarter of the pandemic from March through June 2020, the odds for death decreased dramatically in the next quarter from July through September 2020. However, this relative effect decreased in the next quarter from October through December 2020, and decreased further in the first quarter of 2021 (January through March). This might be due to different virus variants affecting the population, while the CCP used may have come from donors who were infected with a previous variant of SARS-CoV2. Remarkable also are the associations of sex and of cardiovascular disease with mortality at both days 14 and 28.

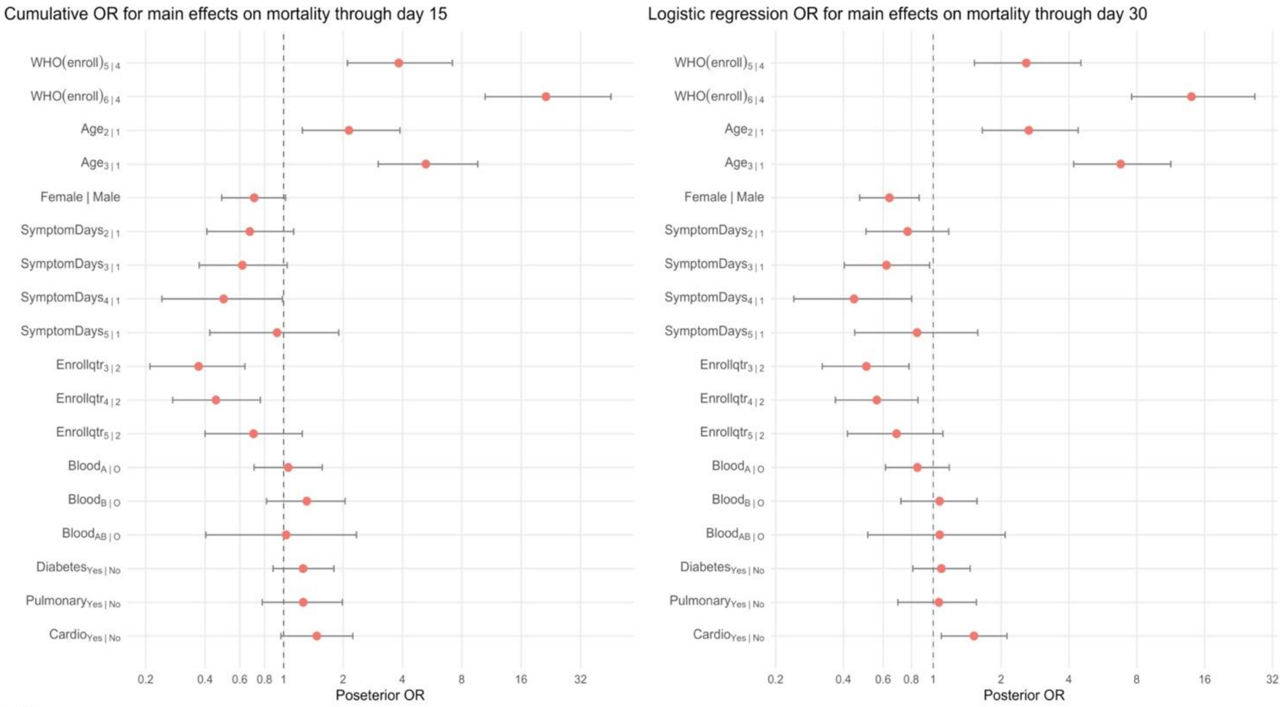

Coding:
Age: 1 = <50; 2 = [50, 65); 3 = 65+
Symptom Days: 1 = 0-3; 2 = 4-6; 3 = 7-10; 4 = 11-14; 5 = 14+
Enrollqtr: 2 = Apr-June; 3 = July-Sept; 4 = Oct-Dec 2020; 5 = Jan-Mar 2021

© 2022 Troxel AB et al. *JAMA Network Open.*

eFigure 12. Heterogeneity of Treatment Effect, Ordinal WHO Score at Day 14

The differential impact of baseline characteristics on association of CCP with outcomes was investigated by including an interaction effect between a covariate and treatment in the main respective models for analysis of each of the six outcomes; see eTable 1. The interaction of each covariate was assessed in separate models, all of which adjusted for the main effects of all covariates in the full set.

The leftmost column gives the posterior probability of OR < 1, and the second from leftmost column gives the posterior probability of OR < 0.8. The three right columns give the 2.5^{th}, 50th, and 97.5^{th} percentiles of the posterior distribution of the OR.

© 2022 Troxel AB et al. *JAMA Network Open.*

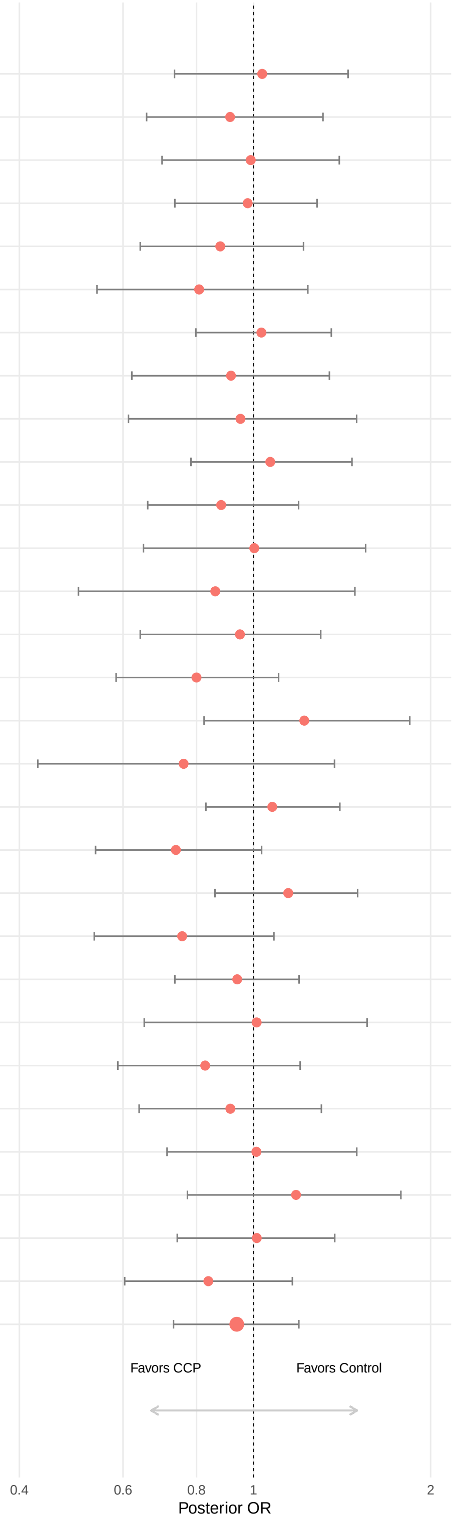

	P(OR<1)	P(OR<0.8)	2.50%	Median	97.50%
Age < 50 (n=602)	0.419	0.068	0.734	1.034	1.448
Age 50−60 (n=828)	0.708	0.217	0.658	0.912	1.312
Age > 65 (n=862)	0.523	0.114	0.699	0.989	1.399
Male (n=1474)	0.568	0.073	0.735	0.977	1.282
Female (n=818)	0.789	0.275	0.642	0.878	1.216
Baseline WHO = 4 (n=448)	0.847	0.479	0.542	0.808	1.237
Baseline WHO = 5 (n=1437)	0.405	0.026	0.798	1.031	1.356
Baseline WHO = 6 (n=407)	0.673	0.243	0.621	0.916	1.346
Symptom duration = 0−3 (n=279)	0.586	0.221	0.613	0.950	1.497
Symptom duration = 4−6 (n=815)	0.328	0.034	0.783	1.068	1.470
Symptom duration = 7−10 (n=818)	0.799	0.259	0.661	0.881	1.193
Symptom duration = 11−14 (n=253)	0.495	0.156	0.650	1.003	1.551
Symptom duration > 14 (n=127)	0.711	0.390	0.504	0.861	1.487
Blood O (n=1063)	0.624	0.180	0.642	0.948	1.301
Blood A (n=768)	0.918	0.500	0.584	0.800	1.103
Blood B (n=371)	0.157	0.018	0.824	1.220	1.843
Blood AB (n=90)	0.821	0.567	0.430	0.761	1.373
Diabetes = No (n=1523)	0.286	0.014	0.830	1.076	1.402
Diabetes = Yes (n=769)	0.967	0.694	0.539	0.738	1.032
CV disease = No (n=1325)	0.162	0.008	0.860	1.145	1.503
CV disease = Yes (n=967)	0.944	0.634	0.536	0.756	1.083
Pulmonary = No (n=2026)	0.699	0.093	0.735	0.938	1.195
Pulmonary = Yes (n=266)	0.478	0.141	0.652	1.012	1.560
Apr−June 2020 (n=621)	0.856	0.424	0.588	0.828	1.200
July−Sept 2020 (n=451)	0.688	0.231	0.639	0.913	1.304
Oct−Dec 2020 (n=868)	0.475	0.089	0.713	1.011	1.498
Jan−Mar 2021 (n=352)	0.209	0.034	0.772	1.181	1.780
CCP 1 unit (AA,CC,DD,GG,RR) (n=1392)	0.466	0.063	0.742	1.013	1.374
CCP 2 units (BB,KK) or 4 units (EE) (n=900)	0.875	0.377	0.604	0.838	1.164
Overall (n=2292)	0.718	0.093	0.731	0.936	1.194

© 2022 Troxel AB et al. *JAMA Network Open.*

eFigure 13. Heterogeneity of Treatment Effect, WHO ≥ 7 at Day 14

The leftmost column gives the posterior probability of OR < 1, and the second from leftmost column gives the posterior probability of OR < 0.8. The three right columns give the 2.5th, 50th, and 97.5th percentiles of the posterior distribution of the OR.

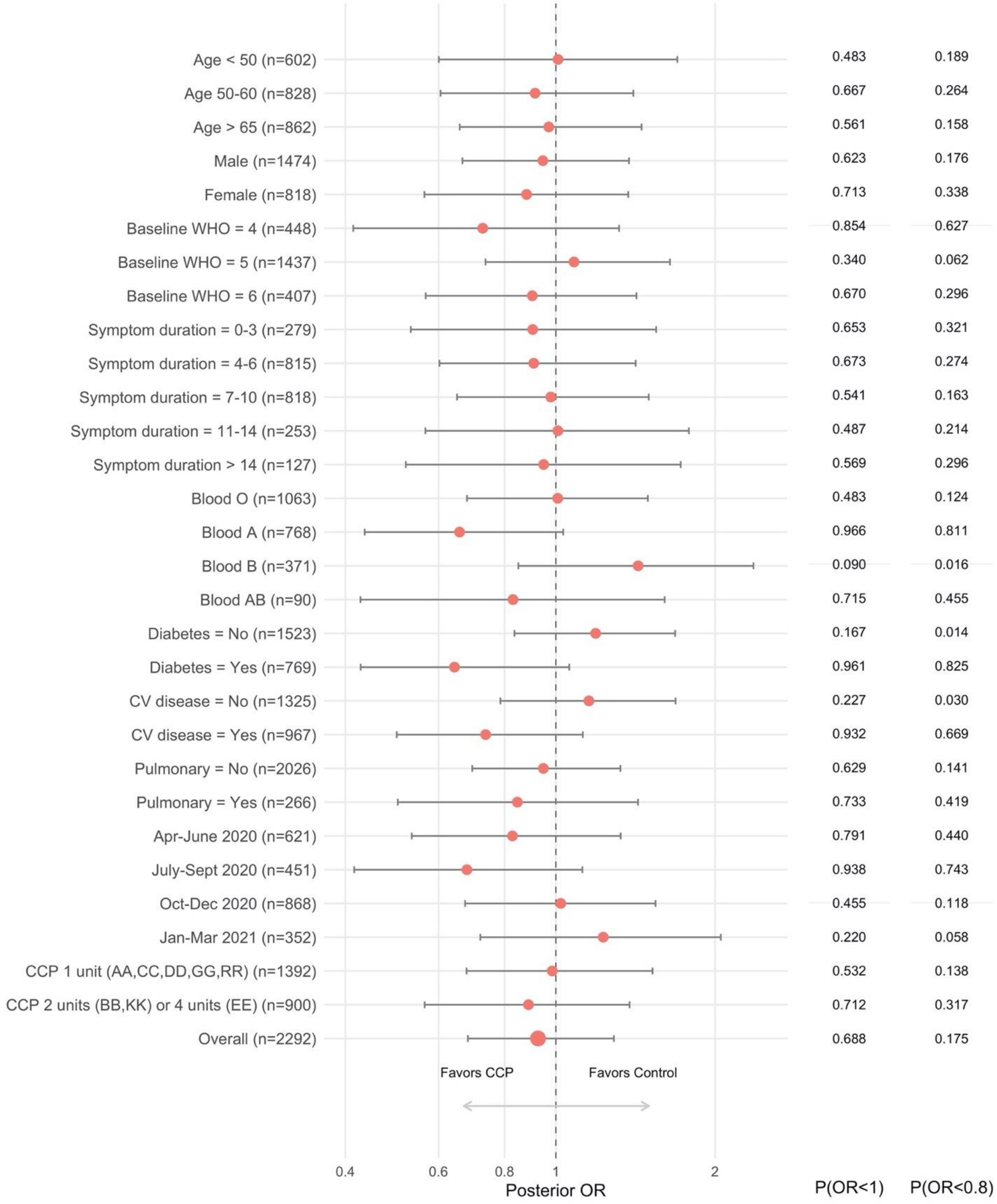

© 2022 Troxel AB et al. *JAMA Network Open.*

eFigure 14. Heterogeneity of Treatment Effect, Mortality at Day 14

The leftmost column gives the posterior probability of OR < 1, and the second from leftmost column gives the posterior probability of OR < 0.8. The three right columns give the 2.5th, 50th, and 97.5th percentiles of the posterior distribution of the OR.

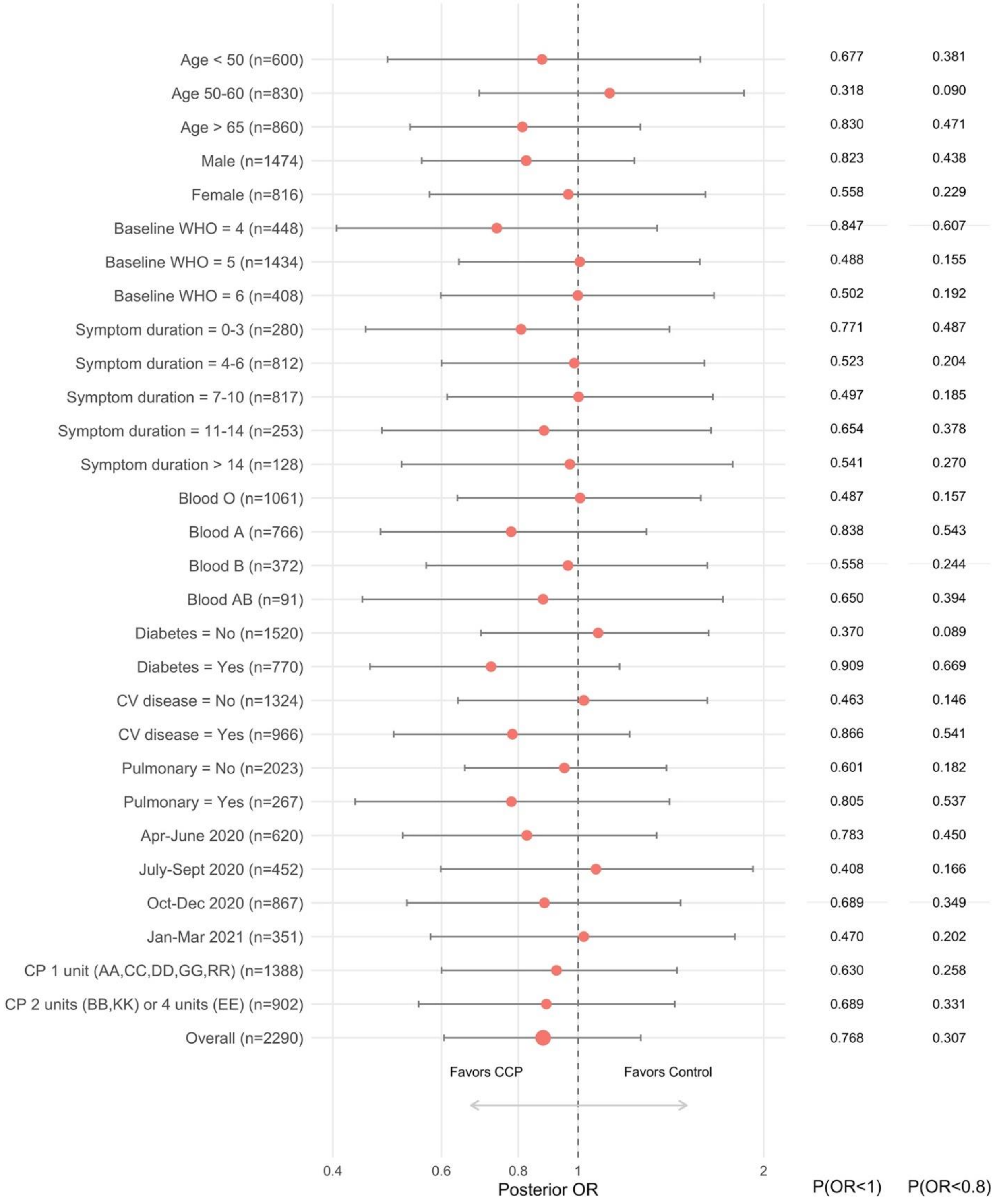

© 2022 Troxel AB et al. *JAMA Network Open.*

eFigure 15. Heterogeneity of Treatment Effect, Ordinal WHO Score at Day 28

The leftmost column gives the posterior probability of OR < 1, and the second from leftmost column gives the posterior probability of OR < 0.8. The three right columns give the 2.5th, 50th, and 97.5th percentiles of the posterior distribution of the OR.

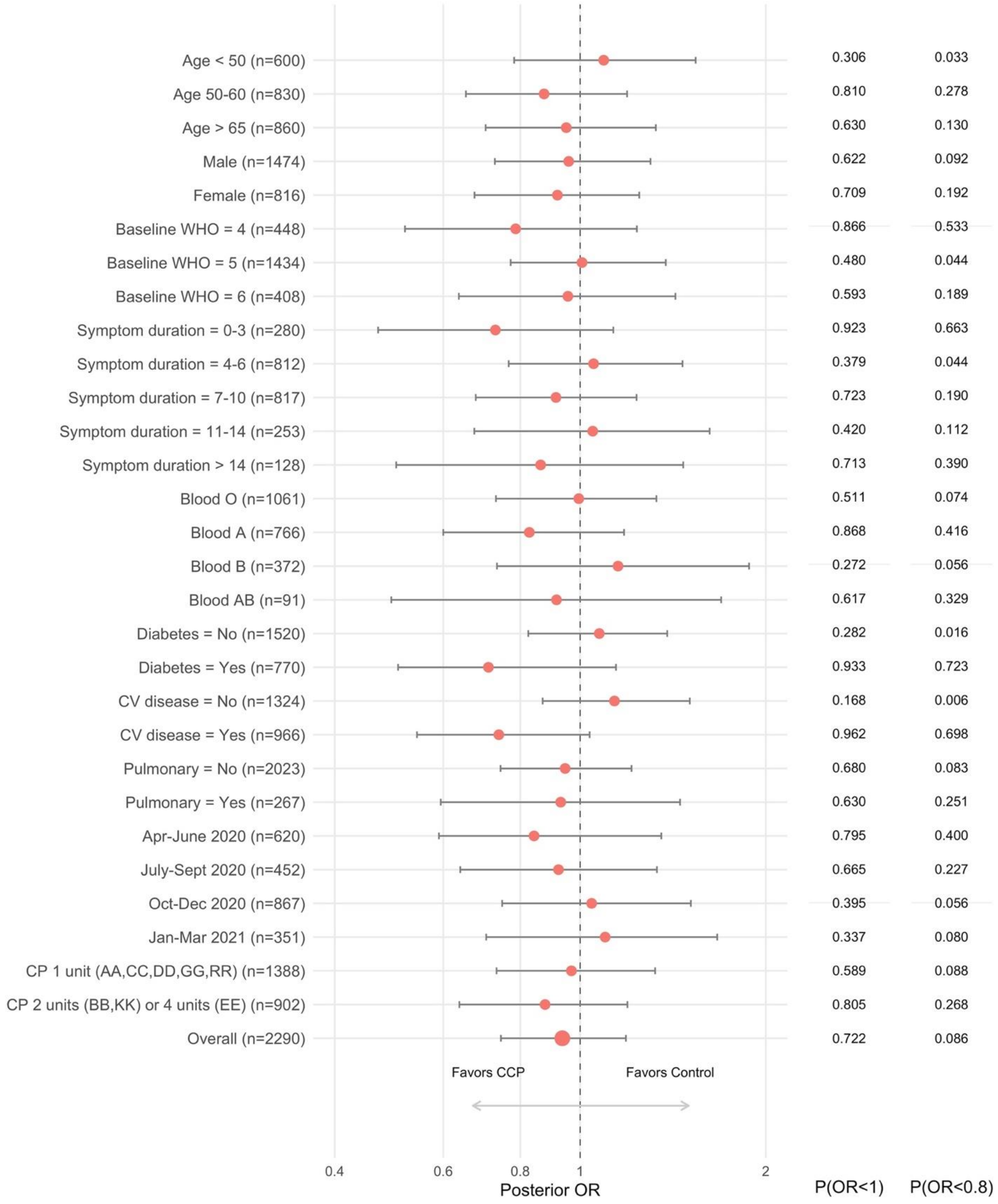

© 2022 Troxel AB et al. *JAMA Network Open.*

eFigure 16: Heterogeneity of Treatment Effect, WHO ≥ 7 at Day 28

The leftmost column gives the posterior probability of OR < 1, and the second from leftmost column gives the posterior probability of OR < 0.8. The three right columns give the 2.5th, 50th, and 97.5th percentiles of the posterior distribution of the OR.

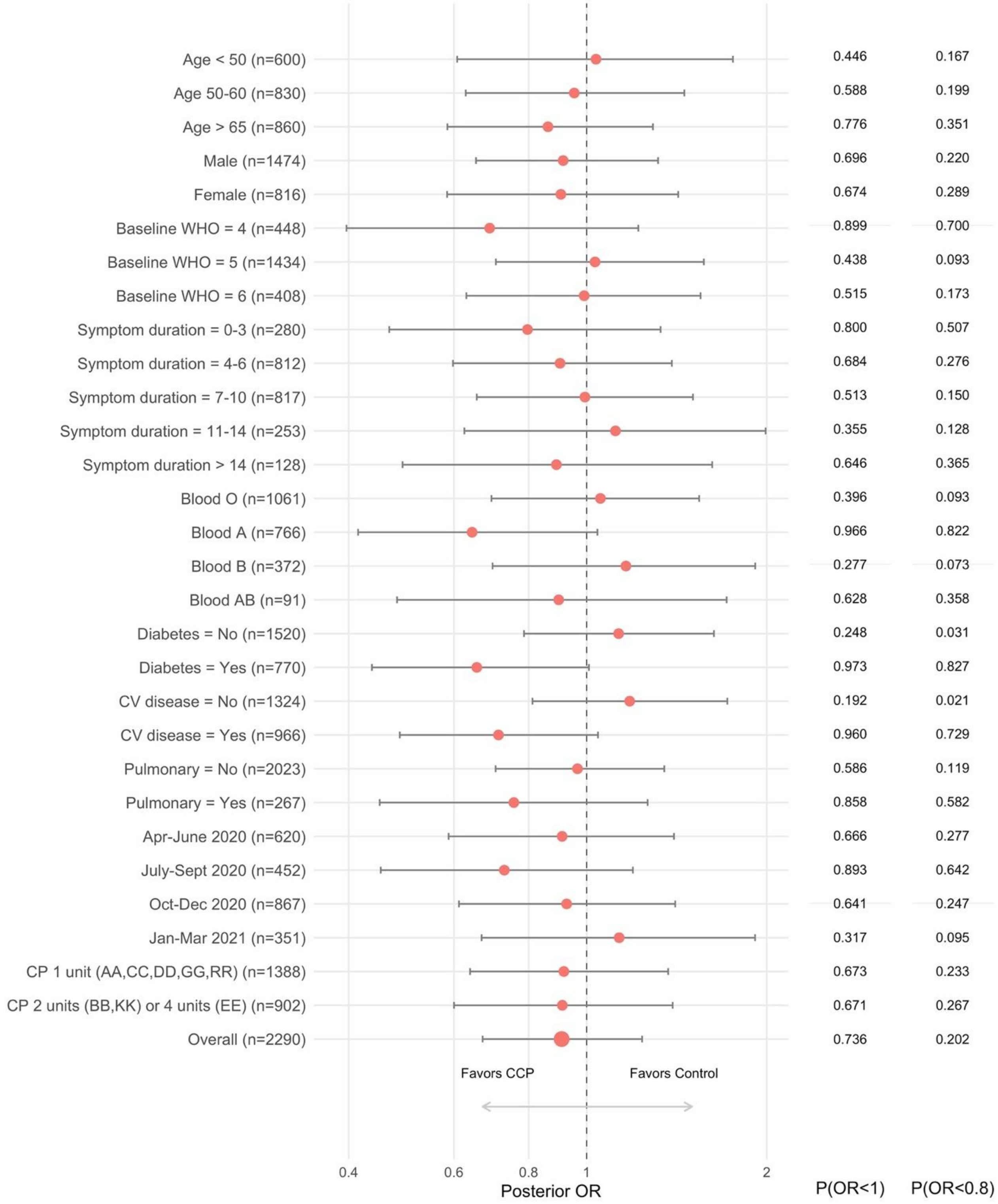

© 2022 Troxel AB et al. *JAMA Network Open.*

eFigure 17. Heterogeneity of Treatment Effect, Mortality at Day 28

The leftmost column gives the posterior probability of OR < 1, and the second from leftmost column gives the posterior probability of OR < 0.8. The three right columns give the 2.5th, 50th, and 97.5th percentiles of the posterior distribution of the OR.

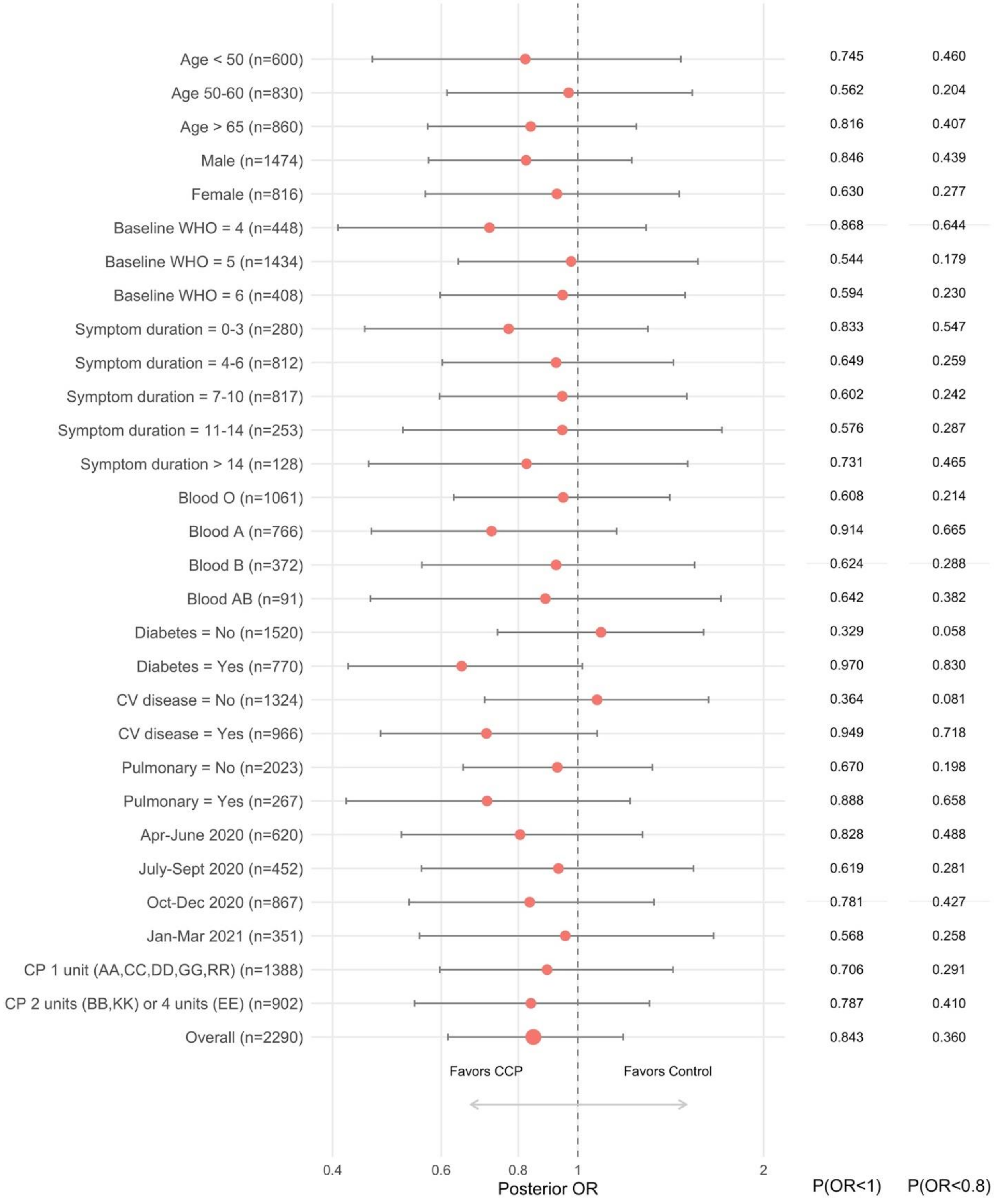

© 2022 Troxel AB et al. *JAMA Network Open.*

eTable 3. Heterogeneity of Treatment Effect: Summary of Outcomes at Day 14

The tables below report the posterior probabilities of the odds ratios estimating the CCP association with outcomes at day 14 and day 28 respectively. Bolded are the cases where the posterior probability of the odds ratios being less than 0.8 is greater than or equal to 50%, i.e., P(OR ≤ 0.8) ≥ 0.5. As discussed in eMethods 2, P(OR<0.8)≥0.5, combined with a high probability of the odds ratio being less than 1 (P(OR<1), i.e., any effect of CCP, conveys strong confidence that there is an effect of CCP and that that effect is more than minimal. The tables suggest that CCP is likely to have more than minimal association in subjects with baseline WHO=4, subjects with blood type A, and patients with pre-existing diabetes and cardiovascular diseases. Those findings are consistent across the three outcomes at both assessment times. It is also likely that CCP has more than minimally association with lower mortality among patients with pre-existing pulmonary disease.

	Ordinal WHO at day 14			WHO > or = 7 at day 14			Mortality (WHO=10) at day 14		
Subgroup	Median	P(OR<1)	P(OR<0.8)	Median	P(OR<1)	P(OR<0.8)	Median	P(OR<1)	P(OR<0.8)
Age < 50 (n=602)	1.034	0.419	0.068	1.010	0.483	0.189	0.874	0.677	0.381
Age 50-60 (n=828)	0.912	0.708	0.217	0.914	0.667	0.264	1.125	0.318	0.090
Age > 65 (n=862)	0.989	0.523	0.114	0.970	0.561	0.158	0.812	0.830	0.471
Male (n=1474)	0.977	0.568	0.073	0.945	0.623	0.176	0.824	0.823	0.438
Female (n=818)	0.878	0.789	0.275	0.881	0.713	0.338	0.964	0.559	0.229
Baseline WHO = 4 (n=448)	0.808	0.847	0.479	0.728	**0.854**	**0.627**	0.738	**0.847**	**0.607**
Baseline WHO = 5 (n=1437)	1.031	0.405	0.026	1.083	0.341	0.063	1.006	0.488	0.155
Baseline WHO = 6 (n=407)	0.916	0.673	0.243	0.903	0.671	0.296	0.999	0.502	0.192
Symptom duration = 0-3 (n=279)	0.950	0.586	0.221	0.904	0.654	0.321	0.808	0.771	0.487
Symptom duration = 4-6 (n=815)	1.068	0.328	0.034	0.908	0.673	0.274	0.986	0.523	0.204
Symptom duration = 7-10 (n=818)	0.881	0.799	0.259	0.978	0.541	0.163	1.002	0.497	0.185
Symptom duration = 11-14 (n=253)	1.003	0.495	0.156	1.009	0.487	0.214	0.881	0.654	0.378
Symptom duration > 14 (n=127)	0.861	0.711	0.390	0.948	0.569	0.296	0.970	0.541	0.270
Blood O (n=1063)	0.948	0.624	0.180	1.009	0.483	0.124	1.008	0.487	0.157
Blood A (n=768)	0.800	**0.918**	**0.500**	0.658	**0.966**	**0.811**	0.779	**0.838**	**0.543**
Blood B (n=371)	1.220	0.157	0.018	1.431	0.090	0.016	0.962	0.559	0.244
Blood AB (n=90)	0.761	**0.821**	**0.567**	0.830	0.715	0.455	0.877	0.650	0.394
Diabetes = No (n=1523)	1.076	0.287	0.014	1.189	0.167	0.014	1.077	0.370	0.089
Diabetes = Yes (n=769)	0.738	**0.967**	**0.694**	0.643	**0.961**	**0.825**	0.723	**0.909**	**0.669**
CV disease = No (n=1325)	1.145	0.162	0.008	1.155	0.227	0.030	1.022	0.463	0.146
CV disease = Yes (n=967)	0.756	**0.944**	**0.634**	0.737	**0.932**	**0.669**	0.782	**0.866**	**0.541**
Pulmonary = No (n=2026)	0.938	0.699	0.093	0.947	0.629	0.141	0.950	0.601	0.183
Pulmonary = Yes (n=266)	1.012	0.478	0.141	0.846	0.733	0.419	0.779	**0.805**	**0.537**
CCP 1 unit (n=1392)	1.013	0.466	0.063	0.985	0.532	0.138	0.922	0.630	0.258
CCP 2 or more units (n=900)	0.838	0.875	0.377	0.888	0.712	0.317	0.888	0.689	0.331
Overall (n=2292)	0.936	0.718	0.093	0.926	0.688	0.175	0.877	0.768	0.307

© 2022 Troxel AB et al. *JAMA Network Open.*

eTable 4. Heterogeneity of Treatment Effect: Summary of Outcomes at Day 28

	Ordinal WHO at day 28			WHO > or = 7 at day 28			Mortality (WHO=10) at day 28		
Subgroup	Median	P(OR<1)	P(OR<0.8)	Median	P(OR<1)	P(OR<0.8)	Median	P(OR<1)	P(OR<0.8)
Age < 50 (n=600)	1.092	0.306	0.033	1.037	0.447	0.167	0.822	0.745	0.460
Age 50-60 (n=830)	0.874	0.810	0.278	0.953	0.588	0.199	0.966	0.562	0.204
Age > 65 (n=860)	0.950	0.630	0.130	0.862	0.776	0.351	0.839	0.816	0.407
Male (n=1474)	0.958	0.622	0.092	0.913	0.696	0.220	0.824	0.846	0.439
Female (n=816)	0.919	0.709	0.192	0.905	0.674	0.289	0.924	0.630	0.277
Baseline WHO = 4 (n=448)	0.786	**0.866**	**0.533**	0.688	**0.899**	**0.700**	0.719	**0.868**	**0.644**
Baseline WHO = 5 (n=1434)	1.007	0.480	0.045	1.033	0.439	0.093	0.975	0.544	0.179
Baseline WHO = 6 (n=408)	0.955	0.593	0.189	0.991	0.515	0.173	0.944	0.594	0.230
Symptom duration = 0-3 (n=280)	0.729	**0.923**	**0.663**	0.796	**0.800**	**0.507**	0.772	**0.833**	**0.548**
Symptom duration = 4-6 (n=812)	1.052	0.379	0.044	0.903	0.684	0.277	0.921	0.649	0.259
Symptom duration = 7-10 (n=817)	0.914	0.723	0.191	0.993	0.513	0.150	0.943	0.602	0.242
Symptom duration = 11-14 (n=253)	1.048	0.420	0.113	1.117	0.355	0.128	0.943	0.576	0.287
Symptom duration > 14 (n=128)	0.863	0.713	0.390	0.890	0.646	0.365	0.825	0.731	0.465
Blood O (n=1061)	0.995	0.511	0.074	1.054	0.396	0.093	0.946	0.608	0.214
Blood A (n=766)	0.827	0.868	0.416	0.643	**0.966**	**0.822**	0.724	**0.914**	**0.665**
Blood B (n=372)	1.152	0.272	0.056	1.163	0.277	0.073	0.922	0.624	0.288
Blood AB (n=91)	0.916	0.617	0.329	0.898	0.628	0.358	0.885	0.642	0.382
Diabetes = No (n=1520)	1.074	0.282	0.016	1.131	0.248	0.031	1.090	0.329	0.058
Diabetes = Yes (n=770)	0.710	**0.933**	**0.723**	0.655	**0.973**	**0.827**	0.647	**0.970**	**0.830**
CV disease = No (n=1324)	1.137	0.169	0.006	1.180	0.192	0.021	1.074	0.364	0.081
CV disease = Yes (n=966)	0.738	**0.962**	**0.698**	0.712	**0.960**	**0.729**	0.711	**0.949**	**0.718**
Pulmonary = No (n=2023)	0.946	0.680	0.083	0.965	0.586	0.119	0.926	0.671	0.198
Pulmonary = Yes (n=267)	0.930	0.630	0.251	0.756	**0.858**	**0.582**	0.712	**0.888**	**0.658**
CP 1 unit (n=1388)	0.968	0.589	0.088	0.916	0.674	0.233	0.891	0.706	0.291
CP 2 or more units (n=902)	0.877	0.805	0.268	0.911	0.671	0.267	0.839	0.787	0.410
Overall (n=2290)	0.936	0.722	0.086	0.908	0.736	0.202	0.847	0.843	0.360

© 2022 Troxel AB et al. *JAMA Network Open.*

eTable 5. Summary of Results

The table below shows the average association of CCP with all outcomes at day 14 and day 28 post-randomization, based on the prespecified analyses. The last two columns give the likelihood that CCP has any association with better outcomes (P(OR<1)) and the likelihood that CCP has more than minimal association with better outcomes (P(OR<0.8)). The column labeled 2.5% is the lower bound and the column labeled 97.5% is the upper bound of the 95% credible intervals for the ORs.

Assessment	Outcome	Adjustment	2.5%	Median	97.5%	P(OR<1)	P(OR<0.8)
Day 14	11-point WHO score	Parsimonious	0.735	0.937	1.187	0.710	0.089
Day 14	WHO score ≥ 7	Parsimonious	0.690	0.940	1.295	0.653	0.154
Day 14	11-point WHO score	Full	0.731	0.936	1.194	0.718	0.093
Day 14	WHO score ≥ 7	Full	0.682	0.926	1.288	0.688	0.175
Day 14	Mortality	Full	0.606	0.877	1.264	0.768	0.307
Day 28	11-point WHO score	Full	0.744	0.936	1.187	0.722	0.086
Day 28	WHO score ≥ 7	Full	0.670	0.908	1.238	0.736	0.202
Day 28	Mortality	Full	0.615	0.847	1.184	0.843	0.360

eTable 6. Summary of Results With Weakly Informative Prior

Assessment	Outcome	Adjustment	2.5%	Median	97.5%	P(OR<1)	P(OR<0.8)
Day 14	11-point WHO score	Parsimonious	0.699	0.928	1.214	0.721	0.129
Day 14	WHO score 7 to 10	Parsimonious	0.650	0.917	1.337	0.693	0.214
Day 14	11-point WHO score	Expanded	0.706	0.928	1.195	0.723	0.130
Day 14	WHO score 7 to 10	Expanded	0.636	0.910	1.345	0.711	0.228
Day 14	Mortality	Expanded	0.545	0.836	1.290	0.799	0.416
Day 28	11-point WHO score	Expanded	0.724	0.928	1.195	0.733	0.110
Day 28	WHO score 7 to 10	Expanded	0.626	0.886	1.267	0.759	0.273
Day 28	Mortality	Expanded	0.556	0.814	1.191	0.871	0.462

eTable 7. Summary of Results With Hypothetical Influential Prior

Assessment	Outcome	Adjustment	2.5%	Median	97.5%	P(OR<1)	P(OR<0.8)
Day 14	11-point WHO score	Parsimonious	0.759	0.919	1.109	0.804	0.073
Day 14	WHO score 7 to 10	Parsimonious	0.729	0.909	1.133	0.805	0.125
Day 14	11- point WHO score	Expanded	0.757	0.918	1.108	0.816	0.079
Day 14	WHO score 7 to 10	Expanded	0.727	0.906	1.128	0.816	0.135
Day 14	Mortality	Expanded	0.694	0.882	1.123	0.846	0.215
Day 28	11- point WHO score	Expanded	0.765	0.918	1.107	0.823	0.069
Day 28	WHO score 7 to 10	Expanded	0.717	0.895	1.117	0.839	0.153
Day 28	Mortality	Expanded	0.695	0.868	1.084	0.896	0.240

© 2022 Troxel AB et al. *JAMA Network Open.*

eTable 8. Summary of Results With Multiple Imputation

Assessment	Outcome	Adjustment	2.5%	Median	97.5%	P(OR<1)	P(OR<0.8)
Day 14	11- point WHO score	Parsimonious	0.742	0.953	1.218	0.658	0.078
Day 14	WHO score 7 to 10	Parsimonious	0.684	0.933	1.296	0.672	0.161
Day 14	11- point WHO score	Expanded	0.742	0.949	1.209	0.672	0.08
Day 14	WHO score 7 to 10	Expanded	0.675	0.921	1.275	0.702	0.183
Day 14	Mortality	Expanded	0.618	0.880	1.263	0.763	0.298
Day 28	11- point WHO score	Expanded	0.743	0.936	1.189	0.716	0.087
Day 28	WHO score 7 to 10	Expanded	0.647	0.882	1.210	0.793	0.263
Day 28	Mortality	Expanded	0.601	0.832	1.170	0.865	0.406

© 2022 Troxel AB et al. *JAMA Network Open.*

eFigure 18. Cochrane RoB Tool Results

Intention-to-treat	Unique ID	Study ID	Experimental	Comparator	Outcome	Weight	D1	D2	D3	D4	D5	Overall
	AA	NA	CCP	S	WHO	1	+	+	+	+	+	+
	BB	NA	CCP	UC	WHO	1	+	+	+	+	+	+
	CC	NA	CCP	UC	WHO	1	+	+	+	+	+	+
	DD	NA	CCP	NCP	NA	1	+	+	+	+	+	+
	EE	NA	CCP	UC	WHO	1	+	+	+	+	+	+
	GG	NA	CCP	UC	WHO	1	+	+	+	+	+	+
	KK	NA	CCP	UC	WHO	1	+	+	+	+	+	+
	RR	NA	CCP	UC	WHO	1	+	+	+	+	+	+

+ Low risk
! Some concerns
- High risk

D1 Randomisation process
D2 Deviations from the intended interventions
D3 Missing outcome data
D4 Measurement of the outcome
D5 Selection of the reported result

© 2022 Troxel AB et al. *JAMA Network Open.*

eAppendix 1. Supplemental Statistical Methods Model specification and prior distributions (begins on next page)

© 2022 Troxel AB et al. *JAMA Network Open.*

S6.B: MODELS SPECIFICATION AND PRIOR DISTRIBUTIONS

1 | PRIMARY EFFICACY ANALYSIS

The primary efficacy outcome is bivariate: (1) clinical status at 14 days $\pm$ 1 day post-randomization, assessed using the WHO 11-point ordinal outcome scale and (2) a binary indicator WHO score between 7 and 10 at 14 days $\pm$ 1 day post-randomization (indicating ventilation requirement or death). While the binary outcome is properly viewed as a subset of the ordinal outcome, we have chosen to accommodate two key functions: efficiency and interpretability. The ordinal scale provides the most efficient use of all available data and provides less variable estimates. The binary outcome is more easily interpreted by clinicians and patients who will ultimately make the treatment decisions. Taken together, the two outcomes provide a more complete picture for the research community with its myriad interests and needs.

1.1 | WHO score at 14 days

The analysis of the first component of the primary outcome will be a cumulative proportional odds model for the ordinal WHO score at 14 days ($\pm$1 day). Let Y be the WHO 11-point scale, ($Y = 0, ..., 10$), with

$$q_y = P(Y = y), y = 0, \ldots, 10, \sum_{y=0}^{10} q_y = 1,$$

and let

$$p_y = P(Y \geq y) = \sum_{s=y}^{10} q_s, \; y = 1, \ldots, 10.$$

Assume that data from K RCTs are available, with n_k subjects in the kth trial, $k = 1, \ldots, K$. Denote the outcome for the ith patient from the kth trial on the 11-point WHO ordinal COVID-19 scale at 14 days ($\pm$1 day) by $Y_{ki} = y$, $y = 0, \ldots, 10$, and let $\boldsymbol{x}_{ki}$ denote a vector of covariates of length $m = 5$ that includes age, sex, baseline WHO score, duration of symptoms before randomization, and quarter of the year when the patient was enrolled (1 = January-March 2020, 2 = April-June 2020, 3 = July-September 2020, 4 = October-December 2020, 5 = January-March 2021). A_{ki} will indicate the treatment assignment for the ith subject in the kth RCT; $A_{ki} = 0$ if the patient was randomized to CP arm and $A_{ki} = 1$ if the patient was randomized to control. The following cumulative proportional odds (co) model for Y_{ki} will be considered:

$$\begin{aligned}
Y_{ki} &\sim \text{Ordinal multinomial}\left(\boldsymbol{p}_{ki}\right) && \boldsymbol{p}_{ki} = \{p_{kiy}\}_0^{10} \\
\text{logit}\left(P\left(Y_{ki} \geq y\right)\right) &= \alpha + \tau_{yk} + \boldsymbol{\beta}\boldsymbol{x}_{ki} + \delta_{k_c} A_{ki} \\
\alpha &\sim \text{Normal}\left(\mu = 0, \sigma = 0.1\right) \\
\tau_{yk} &\sim t_{\text{student}}\left(\text{df} = 3, \mu = 0, \sigma = 8\right) && \text{monotone within } k \\
\boldsymbol{\beta} &\sim \text{Normal}\left(\boldsymbol{\mu} = \boldsymbol{0}, \Sigma = 2.5^2 I_{m\times m}\right) && (1)\\
\delta_{k_c} &\sim \text{Normal}\left(\mu = \delta_c, \sigma = \eta\right) && c = 0, 1, 2 \text{ for the three control conditions} \\
\eta &\sim t_{\text{student}}\left(\text{df} = 3, \mu = 0, \sigma = 0.25\right) \\
\delta_c &\sim \text{Normal}\left(\mu = -\Delta_{co}, \sigma = 0.1\right) \\
-\Delta_{co} &\sim \text{Normal}\left(\mu = 0, \sigma = 0.354\right).
\end{aligned}$$

The parameters p_{kiy} represent the respective probabilities for the ith subject in the kth RCT of being in state y at 14 days($\pm$1 day). The four parameters of the cumulative log-odds model are α, τ_{yk}, $\boldsymbol{\beta}$, and δ_{k_c}. α is a nuisance parameter, which should be very close to 0. However, model fitting improves when α can be freely estimated. $\boldsymbol{\beta}$ is a vector of coefficients for the five baseline covariates.

The τ_{yk}'s represent the RCT-specific intercepts or cut points for the cumulative odds model. Since CP treatment is the reference, the log-odds defined from the cumulative probabilities of the CP arm are estimated by these τ_{yk}'s. All τ_{yk}, $y = 1, \ldots, 10$ satisfy the monotonicity requirements for the intercepts of the proportional odds model (i.e. for all $y > y'$, $\tau_{yk} > \tau_{y'k}$).

δ_{k_c} is the kth RCT-specific "control effect". Because all RCTs will have the experimental treatment arm of convalescent plasma (CP), but may have different control treatment arms, the proposed statistical model has the following notation for *control treatment effect* modeling: c denotes control treatment type and can represent one of three levels: standard of care, $c = 0$; non-convalescent plasma, $c = 1$; saline, $c = 2$. Each of the K RCT's will be associated with one level of c. Each δ_{k_c} will be normally

distributed around a pooled "control effect" δ_c, with a standard deviation η, also to be estimated. η represents the variability in treatment effects across RCTs.

The proposed model conceptualizes the three control conditions as three "treatments" to be compared against the reference condition of CP. Each δ_c is in turn modeled as having a normal distribution around a pooled "control effect" $-\Delta_{co}$. We use $-\Delta_{co}$ so that Δ_{co} will correspond to the difference of log-odds for CP and log-odds for control, rather than control minus CP. Δ_{co}, the key parameter of interest, represents the pooled cumulative odds ratio across all RCT's.

Further details regarding the prior distribution assumptions for the parameters described here are provided at the end of this section in (1.3).

1.2 | Binary Indicator of WHO score between 7 and 10 at 14 days

The analysis of the second component of the primary outcome will be a logistic (l) regression model where the event $W = 1$ if the patient has a WHO score between 7 and 10 at 14 days ($\pm$ 1 day) post-randomization (and $W = 0$ otherwise). The notation largely follows the model described for the first component of the primary outcome.

$$
\begin{aligned}
W_{kt} &\sim \text{Bernoulli}\ (p_{kt}) \\
\text{logit}\left(\boldsymbol{P}\left(W_{kt} = 1\right)\right) &= \tau_k + \boldsymbol{\beta x}_{kt} + \delta_{k_c}\boldsymbol{A}_{kt} \\
\tau_k &\sim t_{\text{student}}\,(\text{df} = 3, \mu = 0, \sigma = 8) \\
\boldsymbol{\beta} &\sim \text{Normal}\ \left(\boldsymbol{\mu} = \boldsymbol{0}, \Sigma = 2.5^2 I_{m\times m}\right) \\
\delta_{k_c} &\sim \text{Normal}\ \left(\mu = \delta_c, \sigma = \eta\right) \qquad\qquad c = 0, 1, 2 \text{ for the three control conditions} \\
\eta &\sim t_{\text{student}}\,(\text{df} = 3, \mu = 0, \sigma = 0.25) \\
\delta_c &\sim \text{Normal}\ \left(\mu = -\Delta_l, \sigma = 0.1\right) \\
-\Delta_l &\sim \text{Normal}\ (\mu = 0, \sigma = 0.354)\,.
\end{aligned}
\tag{2}
$$

The parameters of the logistic model mirror the parameters in the cumulative odds model. The notable difference is that τ_k replaces τ_{yk}, because there is only a single intercept for each RCT. The primary parameter of interest is Δ_l, the pooled log-odds ratio for the binary outcome across all RCT's.

1.3 | Rationale for assumed prior distributions

The prior distributions we will use in Models (1) and (2) (above), and in Models (3), (4), and (5) (below) were selected based on extensive simulations that had three goals: (i) to understand the behavior of the estimating procedure in a variety of realistic situations for the number of the RCTs with different control conditions, sample sizes of the different RCTs, and reasonable-to-anticipate variations in the CP effects across RCTs and between control conditions; (ii) to compare the inferences from the Bayesian analysis with Bayesian monitoring to frequentist analysis with frequentist interim monitoring (with 3 to 5 interim looks) and to anchor the prior distributions to results consistent with inferences from frequentist analyses; this was an identified goal because the clinical community is still more familiar with and more comfortable with inferences from frequentist analyses; and (iii) to assess any convergence issues and sensitivity of the posterior distributions to variations in the postulated priors. Examples of simulation methodology that helped inform these decisions are available online. The simulations were performed in R and Stan.

Prior distributions for parameters can range from skeptical to less skeptical to diffuse. The most skeptical distributions have most of the mass close to zero, which will pull the posterior estimates towards zero. Diffuse priors (e.g. uniform distributions) have mass spread out across the possible range of parameters, and allows the observed data to largely determine the shape of the posterior distribution. The overarching philosophy has been to be conservative (skeptical priors) with respect to efficacy effects, to be moderately conservative (less skeptical priors) with respect to parameters that will not influence decision making but are important to estimate, and to be least conservative or more flexible (diffuse priors) with respect to safety effects (to ensure we do not miss a safety issue) and nuisance parameters (to ensure stable model fitting).

Global intercept for the cumulative proportional odds models

In models (1) and (3), α is a nuisance parameter, which should be very close to 0. However, model fitting improves when α can be freely estimated. We propose a highly informative prior centered around 0, to reflect the belief that this parameter

© 2022 Troxel AB et al. *JAMA Network Open.*

should be 0 while we allow its estimation, resulting in adequate estimation of the pooled treatment effect and regression coefficients.

RCT-specific intercepts/cut points

τ_{yk} are the RCT-specific cut points of the cumulative proportional odds model. They are constrained to be monotonically increasing; the priors for these parameters are based on a modified $t-$distribution with 3 degrees of freedom. (The tails are a compromise between a *Cauchy* distribution and a *normal* distribution with equivalent scale parameters.) Stan implements this through the use of an inverse transformation function, where the MCMC draws are on an unconstrained parameter space and transformed back to the desired monotonic parameters. In the binary outcome models for efficacy (2) and for safety (5) as well as in (4) for evaluating the effect of different antibody levels, the prior distribution for each τ_k is diffuse, which solves the problem by model fitting without the introduction of global intercept.

Covariate coefficients

The covariate coefficients $\boldsymbol{\beta}$ each have a diffuse prior on the log-odds scale, corresponding to little prior information about the effects, and allowing the data to quickly prevail in the estimation. Note that the (relatively) large variance of the Normal distribution ($\sigma = 2.5$) makes the prior diffuse without the need for heavy tails that the $t-$ distributions allow. In this case, the Normal distribution and the t-distribution result in similar posterior distributions for the parameters, but the Normal distribution achieves somewhat better model convergence.

RCT-specific treatment effect

The RCT-specific effects are denoted by δ_k. The prior distribution for the δ_k effect is centered on the control-type effect δ_c associated with that RCT. The variation across RCTs (within each control type) - η in the prior distribution - is a hyperparameter that will be estimated.

Between-RCT variation

The variation across RCTs η will be estimated using an informative prior distribution $t(\text{df} = 3, 0, 0.25)$. The $t-$distribution with df = 3 has wider tails than the slightly more informative Normal$(\mu = 0, \sigma = 0.25)$ distribution.

Control-type effect

The prior distribution for the effects associated with different control conditions (δ_c for efficacy and θ_c for safety) are centered on the pooled efficacy treatment effects $-\Delta_{co}$ and $-\Delta_l$ for the two components of the primary outcome, and on the overall safety treatment effect $-\Theta$, respectively. The three types of control (standard of care, saline and non-convalescent plasma) are not expected to differ greatly from each other. Thus, we impose an informative prior with narrow tails.

Pooled treatment effects

In order to be conservative with respect to the efficacy analysis and to maintain desired operating characteristics of the model, we impose a skeptical prior on the pooled treatment effects Δ_{co} and Δ_l that are centered around 0. The $\sigma = 0.354$ of the Normal priors for the Δ's (on the log-odds ratio scale) corresponds to a prior for the efficacy odds ratio with 95% of the density between 0.5 and 2. With this postulated skeptical prior we ensure that only large amount of information and strong evidence can alter the prior belief.

With respect to safety analysis, we want the flexibility to act as soon as even relatively weak evidence for safety concerns arises. Therefore, we use a diffuse prior for the pooled treatment effect on safety Θ, namely t_{student} (df=3, $0, \sigma = 5.0$).

2 | SECONDARY EFFICACY ANALYSES

Several secondary analyses are planned; see Section 4. Here we outline the analytic principles for investigating interactions between treatment and a pre-specified covariate and the investigation of the effect of the quality and quantity of the CP on its efficacy, by reporting the planned analysis for addressing these specific questions.

2.1 | Effect of duration of COVID-19 symptoms prior to CP transfusion

The clinical understanding of the mechanisms of action of CP indicate that transfused antibodies should be most useful when administered soon after a patient is infected but before the patient's autoimmune system has had time to react while the virus

© 2022 Troxel AB et al. *JAMA Network Open.*

is potentially taking hold. Thus, the effect of duration of symptoms prior to treatment with CP is of high importance, because knowledge of this feature could improve clinical practice. The COMPILE study collects information on duration of symptoms in the format of an ordinal variable, because patients are often uncertain about the precise onset of their symptoms: 0-3 days, 4-6 days, 7-10 days, 11-14 days, and >14 days. To explore the impact of symptom duration on the CP effect on the WHO 11-point score, we will develop an extended version of the models described for the primary outcomes (Section 1). The extended version of the models will include *RCT-specific* treatment by symptom duration interaction parameters $\gamma_{(ks)_c}$, $s \in \{1,2,3,4,5\}$ that are assumed to be normally distributed with a control-type mean γ_{cs}. In this model, there is an indicator variable d_{kis} that equals 1 if the duration of symptoms for the ith patient in the kth RCT falls in duration stratum s, and is 0 otherwise.

The extended version of the Bayesian model (1) is as follows:

$$
\begin{aligned}
Y_{ki} &\sim \text{Ordinal multinomial}\,(\boldsymbol{p}_{ki}) && \boldsymbol{p}_{ki} = \{p_{ki}\}_1^{10} \\
\text{logit}\left(P\left(Y_{ki} \geq y\right)\right) &= \alpha + \tau_{yk} + \boldsymbol{\beta x}_{ki} + A_{ki}\left(\delta_{k_c} + \gamma_{(ks)_c} d_{kis}\right) && s = 1,\ldots,5 \text{ for symptom duration strata} \\
\alpha &\sim \text{Normal}\,(\mu = 0, \sigma = 0.1) && \\
\tau_{yk} &\sim t_{\text{student}}\,(\text{df} = 3, \mu = 0, \sigma = 8)\,, && \text{monotone within } k \\
\boldsymbol{\beta} &\sim \text{Normal}\left(\boldsymbol{\mu} = \boldsymbol{0}, \Sigma = 2.5^2 I_{m\times m}\right) && \\
\delta_{k_c} &\sim \text{Normal}\left(\mu = \delta_c, \sigma = \eta\right) && c = 0,1,2 \text{ for the three control conditions} \\
\eta &\sim t_{\text{student}}\,(\text{df} = 3, \mu = 0, \sigma = 0.25) && \\
\delta_c &\sim \text{Normal}\,(\mu = -\Delta, \sigma = 0.1) && \\
-\Delta &\sim t_{\text{student}}\,(\text{df} = 3, \mu = 0, \sigma = 2.5) && \\
\gamma_{(ks)_c} &\sim \text{Normal}\left(\mu = \gamma_{cs}, \sigma = 1\right) && \\
\gamma_{cs} &\sim \text{Normal}\left(\mu = -\Gamma_s, \sigma = 0.25\right) && \\
-\Gamma_s &\sim t_{\text{student}}\,(\text{df} = 3, \mu = 0, \sigma = 1.5) &&
\end{aligned}
\qquad (3)
$$

The pooled effect of CP (on the log-odds scale) across all RCTs for patients with symptom duration s will be $\Delta_s = \Delta + \Gamma_s$. In this exploratory analysis, we will estimate the posterior probability for Δ_s at each level of s to identify subgroups that might warrant further study.

Model (2) for the binary component of the primary outcome will be extended in a similar way to evaluate the interaction between treatment and duration of symptoms. Similar models will be employed to evaluate the interactions between treatment and sex, age, and baseline clinical status (measured by WHO 11-point scale) on the primary and secondary outcomes.

2.2 | Effect of donor CP antibodies on the efficacy of CP

The primary analysis of COMPILE will address the question *whether treatment with convalescent plasma (yes/no) is efficacious against any control treatment (standard of care, non-convalescent plasma or saline)*. The statistical models to address this primary question are discussed in Section 1. A second and equally important question that COMPILE aims to address is whether the *quantity* of CP that was transfused and/or the *amount* of antibodies in the CP matters and if so, how the quantity of CP and/or the amount of antibodies are related to the efficacy of treatment with CP. There are several ways to characterize the quality and quantity of CP. **First**, CP for transfusion comes in standardized units of sizes 250-300ml and the CP treatment in the RCTs is indicated by the number of units. For example, RCTs collaborating in COMPILE used 1, 2, or 4 units of CP. **Second**, in order for a sample of plasma to be considered convalescent for SARS-CoV-2, it must contain a certain amount of anti-SARS-CoV-2 antibodies. While guidelines regarding which measurement platforms should be used to assess the potency of the CP are beginning to emerge, RCTs across the globe have used different platforms that sometimes measure different types of antibodies. The COMPILE Antibodies Subcommittee conducted an investigation to enable conversion of measurements of antibodies obtained on different platforms in the different RCTs to a uniform scale. The Subcommittee recommended that the CP levels be classified into two groups – one reflecting *low* levels (i.e., levels that are expected to be insufficient) or a second reflecting levels of antibodies that are *not low*. The **third** and most rigorous approach for assessing the effect of antibody levels on the efficacy of CP is based on measurements of antibody titer in samples from *all* transfused CP units performed on the same platform. Samples from almost all transfused CP units are preserved in all clinical trials. Obtaining those measurements requires coordination that will take time, but this will provide the most definitive answer to the question. In the meantime, we will use

© 2022 Troxel AB et al. *JAMA Network Open.*

the measures described in the first two options as a surrogate for the actual antibody titers from the third option. Below is the proposed analytic model that assumes that treatment is scored on a 3-point scale according to the second alternative:

- zero antibodies – subjects randomized to the control condition in the RCTs will be considered to have received this level of treatment;
- low level antibodies – subjects in the CP arm of the RCTs who received CP classified as *low* level according to the scale proposed by the Antibodies Subcommittee;
- not low level antibodies – subjects in the CP arm of the RCTs, who received CP classified as *not low level* according to the scale proposed by the Antibodies Subcommittee.

Just as in the primary outcome model (1), the outcome is the WHO 11-point score at Day 14±1. The observed data are Y_{kt}, the individual WHO score for the ith patient in the kth study; $\boldsymbol{x}_{kt}$ is a vector of covariates as in the previous models, and a_{kt} takes a value of 0, 1, or 2, depending on the level of antibodies. For those randomized to the control condition, $a_{kt} = 0$.

For the purposes of addressing the specific question about the effect of the amount of antibodies, all control conditions are considered the same, because the amount of antibodies received by the patients in the control arms is zero. In the model below, the cumulative odds for patients receiving zero antibodies will be reflected in τ_{yk}, the study-specific baseline log cumulative odds. The following model is proposed for the evaluation of the amount of antibodies in the CP:

$$
\begin{aligned}
Y_{kt} &\sim \text{Ordinal multinomial}\left(\boldsymbol{p}_{kt}\right) && \boldsymbol{p}_{kt} = \{p_{kt}\}_1^{10} \\
\text{logit}\left(\boldsymbol{P}\left(Y_{kt} \geq y\right)\right) &= \alpha + \tau_{yk} + \boldsymbol{\beta x}_{kt} + \delta_{kt} I\left(a_{kt} = t\right) && t = 1 \text{ or } 2 \text{ for low and not low levels of antibodies} \\
\alpha &\sim \text{Normal}\left(\mu = 0, \sigma = 0.1\right) \\
\tau_{yk} &\sim t_{\text{student}}\left(\text{df} = 3, \mu = 0, \sigma = 8\right) && \text{monotone within } k \\
\boldsymbol{\beta} &\sim \text{Normal}\left(\boldsymbol{\mu} = \boldsymbol{0}, \Sigma = 2.5^2 I_{m\times m}\right) \\
\delta_{kt} &\sim \text{Normal}\left(\mu = \delta_t, \sigma = \eta\right) \\
\eta &\sim t_{\text{student}}\left(\text{df} = 3, \mu = 0, \sigma = 0.25\right) \\
\delta_t &\sim \text{Normal}\left(\mu = 0, \sigma = 0.354\right)
\end{aligned}
\tag{4}
$$

If it is possible to measure the antibodies on a single platform so that the measures across RCTs (or a subset of RCTs) are directly comparable, we could extend the model further to include a continuous exposure Z_{kt}:

$$\text{logit}\left(\boldsymbol{P}\left(Y_{kt} \geq y\right)\right) = \alpha + \tau_{yk} + \boldsymbol{\beta x}_{kt} + \delta_k Z_{kt}$$

3 | TERTIARY EFFICACY ANALYSES

Mortality and time to hospital discharge

The tertiary outcomes will include overall mortality (time to death) and time to hospital discharge. The analysis of overall mortality will be based on a (frequentist) log-rank stratified by RCT. Cox proportional hazards models will be employed to adjust for the covariates in the comprehensive covariates list (age, sex, etc.) and to evaluate interactions of baseline characteristics with treatment. The proportional hazards assumption will be evaluated using the method of cumulative martingale residuals.

Time to discharge (also to be analyzed using frequentist methods) is defined as the duration from randomization to hospital discharge to home, acute, or long-term care facilities. Death before discharge is a competing risk event that precludes a successful discharge and thus will be properly accounted in the analysis of time to discharge. Gray's test will be used to compare the sub-distribution hazards (cumulative incidence function, CIF) of time to discharge between treatment groups. The Fine-Gray regression model will be employed to estimate treatment effect on the CIF adjusting for the comprehensive list of covariates.

Precision medicine analysis

A very important question for patients, clinicians, and researchers is to determine *what are the patient characteristics associated with the greatest benefit from treatment with CP.* These questions can be addressed using precision medicine methodology. We will employ existing and newly developed methodologies to identify biosignatures for response to CP

© 2022 Troxel AB et al. *JAMA Network Open.*

treatment. Biosignatures are patient characteristics, or more likely combination of such characteristics, that are associated with heterogeneity of treatment effect. In its simplest form, a biosignature is a continuous variable (e.g., a linear combination of baseline patient characteristics) that has a strong (large in magnitude, significant) interaction with the treatment indicator in the model for the outcome. The methodologies developed for discovery of such biosignatures for treatment response fall under the rubric of developing optimal treatment decision rules; based on what is known about the patient at the time of treatment decision making, the goal is to give a particular treatment only to patients who are likely to benefit. Precision medicine is a highly active area of research, and new approaches are constantly being developed to address ever more complex clinical circumstances.

4 | SUMMARY OF EFFICACY ANALYSES

Table 1 provides a schematic representation of all the analyses that we plan to conduct for five outcomes: **WHO score at day 14±1**, **WHO score at day 28±2**, **mortality at day 14±1**, **mortality at day 28±2**, and **time to discharge**. Stopping rules for efficacy will be based on the non-interaction models of the bivariate primary outcome **WHO score at day 14±1** and **WHO score 7-10 (yes/no)**; the treatment comparison is **any CP vs. Control = 1, 2, and 3**. Stopping COMPILE for efficacy will be considered if both primary endpoints are met (see Section 6).

TABLE 1 Planned Analyses

Description	Day	Adjustment	Interaction models			
			Age	Sex	Symptoms Duration	WHO Baseline
**Primary analysis: comparison of CP vs. Control (3 types)**						
1. WHO score: cum. prop. OR	14	parsimonious[1]	x	x	x	x
2. WHO 7-10 (yes/no)	14	parsimonious	x	x	x	x
**Secondary analyses: comparison of CP vs. Control (3 types)**						
3. WHO score: cum. prop. OR	14	expanded[2]	x	x	x	x
4. WHO 7-10 (yes/no)	14	expanded	x	x	x	x
5. WHO score: cum. prop. OR	28	expanded	x	x	x	x
6. WHO 7-10 (yes/no)	28	expanded	x	x	x	x
**Tertiary analyses: comparison of CP vs. Control (3 types)**						
7. All-cause mortality (yes/no)	14	expanded	x	x	x	x
8. All-cause mortality (yes/no)	28	expanded	x	x	x	x
9. Time to discharge		expanded	x	x	x	x
**Tertiary analyses: dose-response (comparison of no CP vs. different number of CP units or levels of AB[3])**						
10. WHO score: cum. prop. OR	14	expanded	x	x	x	x
11. WHO 7-10 (yes/no)	14	expanded	x	x	x	x
12. WHO score: cum. prop. OR	28	expanded	x	x	x	x
13. WHO 7-10 (yes/no)	28	expanded	x	x	x	x
14. All-cause mortality (yes/no)	14	expanded	x	x	x	x
15. All-cause mortality (yes/no)	28	expanded	x	x	x	x
16. Time to discharge		expanded	x	x	x	x

[1] Parsimonious adjustment includes age, sex, WHO score at baseline, days since symptom onset and quarter when the patient was enrolled in the RCT.

[2] Expanded adjustment also includes past medical history and concomitant medications at time of randomization.

[3] AB = antibodies

© 2022 Troxel AB et al. *JAMA Network Open.*

treatment. Biosignatures are patient characteristics, or more likely combination of such characteristics, that are associated with heterogeneity of treatment effect. In its simplest form, a biosignature is a continuous variable (e.g., a linear combination of baseline patient characteristics) that has a strong (large in magnitude, significant) interaction with the treatment indicator in the model for the outcome. The methodologies developed for discovery of such biosignatures for treatment response fall under the rubric of developing optimal treatment decision rules; based on what is known about the patient at the time of treatment decision making, the goal is to give a particular treatment only to patients who are likely to benefit. Precision medicine is a highly active area of research, and new approaches are constantly being developed to address ever more complex clinical circumstances.

4 | SUMMARY OF EFFICACY ANALYSES

Table 1 provides a schematic representation of all the analyses that we plan to conduct for five outcomes: **WHO score at day 14±1**, **WHO score at day 28±2**, **mortality at day 14±1**, **mortality at day 28±2**, and **time to discharge**. Stopping rules for efficacy will be based on the non-interaction models of the bivariate primary outcome **WHO score at day 14±1** and **WHO score 7-10 (yes/no)**; the treatment comparison is **any CP vs. Control = 1, 2, and 3**. Stopping COMPILE for efficacy will be considered if both primary endpoints are met (see Section 6).

TABLE 1 Planned Analyses

Description	Day	Adjustment	Interaction models			
			Age	Sex	Symptoms Duration	WHO Baseline
**Primary analysis: comparison of CP vs. Control (3 types)**						
1. WHO score: cum. prop. OR	14	parsimonious[1]	x	x	x	x
2. WHO 7-10 (yes/no)	14	parsimonious	x	x	x	x
**Secondary analyses: comparison of CP vs. Control (3 types)**						
3. WHO score: cum. prop. OR	14	expanded[2]	x	x	x	x
4. WHO 7-10 (yes/no)	14	expanded	x	x	x	x
5. WHO score: cum. prop. OR	28	expanded	x	x	x	x
6. WHO 7-10 (yes/no)	28	expanded	x	x	x	x
**Tertiary analyses: comparison of CP vs. Control (3 types)**						
7. All-cause mortality (yes/no)	14	expanded	x	x	x	x
8. All-cause mortality (yes/no)	28	expanded	x	x	x	x
9. Time to discharge		expanded	x	x	x	x
**Tertiary analyses: dose-response (comparison of no CP vs. different number of CP units or levels of AB[3])**						
10. WHO score: cum. prop. OR	14	expanded	x	x	x	x
11. WHO 7-10 (yes/no)	14	expanded	x	x	x	x
12. WHO score: cum. prop. OR	28	expanded	x	x	x	x
13. WHO 7-10 (yes/no)	28	expanded	x	x	x	x
14. All-cause mortality (yes/no)	14	expanded	x	x	x	x
15. All-cause mortality (yes/no)	28	expanded	x	x	x	x
16. Time to discharge		expanded	x	x	x	x

[1] Parsimonious adjustment includes age, sex, WHO score at baseline, days since symptom onset and quarter when the patient was enrolled in the RCT.

[2] Expanded adjustment also includes past medical history and concomitant medications at time of randomization.

[3] AB = antibodies

© 2022 Troxel AB et al. *JAMA Network Open.*

When $OR_{co} < 1$ and $OR_l < 1$, CP is more effective than control; we will require a very high level of certainty that this is the case. When $OR_{co} < 0.8$ and $OR_l < 0.8$, it is considered that the beneficial effect of CP is more than trivial; we will require a moderate level of certainty that this is the case. *The study will not be stopped unless all four criteria are met.*

Stopping for harm

Stopping for harm will be based on the models used for the primary efficacy analyses (1) and (2). Evidence for harm due to CP will be based on the same odds ratios used in assessing evidence for efficacy. Observing odds ratios (OR_{co} or OR_l) that exceed 1 will indicate that CP is less effective than control (i.e., CP is harmful). The stopping rule for harm is:

$$P\left(OR_{co} > 1\right) \geq 0.80 \quad \text{or} \quad P\left(OR_l > 1\right) \geq 0.80.$$

Note that the stopping rule for harm is much less stringent than the stopping rule for efficacy: the required level of certainty about possible harm is set at a lower threshold (0.80) than the level of certainty concerning efficacy (0.95); furthermore, it is sufficient if this lower level of certainty is satisfied with respect to only one of the bivariate outcomes, not both as in the case of assessing efficacy.

Stopping for safety

In the logistic regression model Section 5 for evaluating safety, described in Section 5, the parameter of interest (the overall CP effect on safety) is Θ. We propose stopping for safety based on the posterior probability for the odds ratio (OR) of adverse events in the CP condition compared to the control condition ($OR_{ae} = e^{\Theta}$). The proposed stopping rule enforces considerations for stopping for safety reasons, even if only a relatively weak evidence for safety concerns is observed:

$$P(OR_{ae} > 1) \geq 0.75.$$

No stopping rules based on symptoms duration and donor CP antibodies The COMPILE study does not have stopping rules based on the analysis of symptoms duraration and donor CP antibodies. At the end of COMPILE (due to either achieving one of the stopping criteria based on the primary analyses or if all studies have stopped recruitment and completed follow up) the posterior probabilities of Γ_s, $s = 1$ to 5 from (3) and of $\delta_t, t = 1$ or 2 from (4) will be used to make recommendations regarding the effect of symptom duration of the efficacy of CP and the therapeutic effects of CP with different levels of antibodies, respectively.

☐

© 2022 Troxel AB et al. *JAMA Network Open.*

Primer on bayesian inference

Here we give an explanation about how evidence from the bayesian posterior distributions of the odds ratios should be interpreted and how this interpretation contrasts with the more common frequentist reporting of evidence in terms of p-values.

Prior probability distribution and posterior probability distribution

A **probability distribution** of an odds ratio (OR) encapsulates all the information we have about the size of the OR. A probability distribution allows us to compute, for example, the confidence we have that the OR is within a specific interval, say, that the OR is less than 1 [P(OR < 1)]. The **prior** probability distribution is our knowledge about the OR up until a particular point in time, before we have any more information about the OR, i.e., **before any data are observed**. The **posterior** probability distribution characterizes the state of our knowledge about the OR **after the data are observed**.

Posterior probability

The posterior probability distribution allows us to quantify the confidence we have that the OR is, for example, less than 1 [P(OR < 1)]. An OR < 1 indicates that better outcomes are associated with treatment with CCP. The posterior probability for OR < 1 [P(OR < 1)] tells us how confident we can be that CCP has some association better outcomes; for example, P(OR<1) > 90% suggests we can be quite confident that CCP is associated with better outcomes.

However, with a large sample size, this posterior probability of effectiveness can increase considerably, even if the median of the OR is very close to 1; for example, median OR = 0.93. To ensure that beyond the presence of any association, the CCP association is large enough to be clinically meaningful, a useful characteristic of the posterior probability distribution is the quantity P(OR < 0.8). An OR = 0.8 means that the odds for undesirable outcome under the control treatment are reduced by 20% with CCP. Such an effect can be considered more than a minimal association. With P(OR < 0.8) ≥ 50%, we can have some confidence in the statement: "it is likely that the association of CCP with better outcomes is more than minimal". When the combined conditions P(OR < 1) ≥ 90% and P(OR < 0.8) ≥ 50% are satisfied, our confidence that CCP is more than minimally associated with better outcomes can be quite strong.

Bayesian credible intervals vs. frequentist confidence intervals

Bayesian credible intervals (CrIs) convey different information than frequentist confidence intervals (CIs). A frequentist confidence interval refers to confidence in the analytic procedure (see below). The bayesian credible interval refers to the confidence that the estimate of interest (here the OR comparing CCP and control) lies within a particular interval. Therefore, the forest plots shown in eFigures 12-17 should not be interpreted in the same way as frequentist CIs.

Next, we provide more discussion on the difference between bayesian credible intervals (CrIs) and frequentist confidence intervals (CIs). For illustration we focus on 95% intervals. A frequentist 95% CI either includes or excludes the true but unknown effect (OR comparing CCP and control), and the probability that it includes the true but unknown OR is 95%. The confidence in the procedure means that if we repeated the procedure many times (i.e., performed the same experiment, collected

© 2022 Troxel AB et al. *JAMA Network Open.*

data, fit the models we used, and estimated the OR and the 95% frequentist CIs), 95% of those CIs would contain the true OR. For any given experiment, we cannot know whether our particular 95% CI contains the true OR or not, but we are confident in the procedure that we used, because under repeated experimentation, 95% of the CIs would cover the true OR. On the other hand, a 95% bayesian credible interval tells us that, given the data we observed and prior information, the probability that the OR is in the interval (a, b) is 95%: this statement incorporates the uncertainty about the distribution of the OR of interest, given the prior information and data.

Likelihood of the data given the hypothesis vs. likelihood of the hypothesis given the data

Another contrast between the bayesian and frequentist frameworks for making inferences and statements about uncertainty is the following. P-values indicate the likelihood of the data given the (null) hypothesis, i.e., how (un)likely is it for what we observed to happen, if the null hypothesis (OR = 1) were true? In other words, the p-value is a conditional **probability of the data, given a (null) hypothesis**. A bayesian posterior probability, on the other hand, assesses the **probability of a hypothesis given the data**. For example, P(OR < 1) is the conditional probability that CCP has an effect (i.e., OR < 1) given the observed data. As noted above, having both P(OR < 1) ≥ 90% and P(OR < 0.8) ≥ 50% indicates strongly that CCP is associated with better outcomes compared to control.

© 2022 Troxel AB et al. *JAMA Network Open.*

Sensitivity analyses and Cochrane Risk of Bias assessment

We performed additional analyses to evaluate the sensitivity of the results to several data analytic decisions. A summary of the sensitivity investigations is presented below.

1. Effect of different priors for the treatment effect parameter
    a. The results reported in the paper used a skeptical prior for the treatment parameter of interest: the log odds ratio (OR) for CCP vs. control (Δ) was assumed to have normal distribution with mean 0 and standard deviation σ =0.354, i.e., N(mean=0, σ = 0.354), thus constraining 95% of the distribution of the log OR to be between 0.5 and 2. Results are shown in eTable 3.
    b. To assess the sensitivity of the results to this assumption, we repeated all analyses assuming the prior distribution for the log OR to be less skeptical, i.e., Δ ~ N(mean=0, σ =2.5). The relatively large variance of the Normal distribution (σ=2.5) makes the prior weakly informative. With σ=2.5, the Normal distribution and the t-distribution (df=3,0, σ=2.5) result in similar posterior distributions for the parameters, but the Normal distribution achieves better model convergence. Results are shown in eTable 6. The posterior distributions of the estimated ORs are very similar in the case of the skeptical and less skeptical priors. This was expected given the large sample size of the COMPILE data set.
    c. Next, we assumed a hypothetical scenario that data from another large CCP RCT (not currently part of COMPILE) became available and could influence the priors used in the model. This was examined by assuming a different prior that favors CCP. Thus, we assumed the prior Δ ~ N(-0.1, σ =0.15). This prior corresponds approximately to the posterior from an RCT with n=1000, that started with our original prior for Δ ~ N(0, σ =0.354) and ended up with a median OR=0.9. Results are shown in eTable 7. As with the skeptical prior, the results remain essentially unchanged; given the large sample size of the COMPILE study, the data overwhelms the prior distribution and results in similar posteriors

2. Multiple imputation of missing data in both covariates and outcomes (day 14 and 28 WHO score)

Multiple imputations (n=50) were conducted separately for the CCP group and control groups within each RCT. We used the following variables in the multiple imputations:
- Expanded covariates at randomization (age, sex, WHO score, symptom duration, quarter of enrollment, blood type, history of diabetes, history of pulmonary disease, history of cardiovascular disease) *
- Indicator of whether or not the patient was discharged between randomization and day 7 **
- Indicator of whether or not the patient was discharged between day 8 and day 15 **
- Daily WHO score assessed on the day closest to day 14 and the number of days from that time point to day 14 **
- Day 14 WHO score **
- Indicator of whether or not the patient was discharged between day 16 and day 23 ***
- Indicator of whether or not the patient was discharged between day 24 and day 30 ***
- Daily WHO score assessed on the day closest to day 28 and the number of days from that time point to day 28 ***
- Day 28 WHO score ***

© 2022 Troxel AB et al. *JAMA Network Open.*

The multiple imputations were done in a monotone way such that only baseline information (*) was used to impute baseline information, day 14 outcome was imputed by the information accrued by day 14 (*/**), and day 28 outcome was imputed by all the available data up to day 30 (*/**/***). Results are shown in eTable 8. They show little sensitivity to our approach to missing data.

3. We conducted a Cochrane risk of bias (RoB) assessment using the online tool, available at https://sites.google.com/site/riskofbiastool/welcome/rob-2-0-tool/current-version-of-rob-2?authuser=0. One author (ABT) rated each of the eight RCTs on the five specified domains: randomization process, deviations from intended interventions, missing outcome data, measurement of the outcome, and selection of the reported result. The RCTs were each judged to have a low risk of bias on each domain. The summary is shown in eFigure 18.

© 2022 Troxel AB et al. *JAMA Network Open.*

eAppendix 2. Committee Rosters

Steering Committee

Country/RCT	Location	Name	Role on RCT	Role on COMPILE
COMPILE	NYU	Andrea Troxel (Chair)	COMPILE PI	Chair of SC
USA/CONTAIN	NYU	Mila Ortigoza	PI	Member of SC
USA/CONTAIN	Einstein	Liise-anne Pirofski	PI	Member of SC
Spain	Madrid	Cristina Avendano-Sola	PI	Member of SC
Spain	Madrid	Rafael Duarte	PI	Member of SC
USA/UCSF	San Francisco	Annie Luetkemeyer	PI	Member of SC
USA/UCSF	San Francisco	Priscilla Hsue	PI	Member of SC
USA/UPenn	Philadelphia	Katharine Bar	PI	Member of SC
Brazil	Brasilia	Andre Moraes Nicola	PI	Member of SC
Netherlands	Rotterdam	Bart JA Rijnders	PI	Member of SC
Belgium	Leuven	Geert Meyfroidt	PI	Member of SC
Belgium	Leuven	Timothy Devos	PI	Member of SC
India	New Delhi	Aparna Mukherjee	PI	Member of SC

© 2022 Troxel AB et al. *JAMA Network Open.*

Publications Committee

Country/RCT	Location	Name	Role on RCT	Role on COMPILE
USA/CONTAIN	NYU	Mila Ortigoza (Chair)	PI	Chair of PC
USA/CONTAIN	NYC Einstein	Liise-anne Pirofski (Chair)	PI	Chair of PC
USA/COMPILE	NYU	Andrea Troxel	PI	Member of PC
USA/UCSF	San Francisco	Annie Luetkemeyer	PI	Member of PC
USA/UCSF	San Francisco	Priscilla Hsue	PI	Member of PC
USA/UCSF	San Francisco	Emma Bainbridge	Co-Investigator	Member of PC
USA/UPenn	Philadelphia	Katharine Bar	PI	Member of PC
Spain	Madrid	Cristina Avendano-Sola	PI	Member of PC
Spain	Madrid	Rafael Duarte	PI	Member of PC
Netherlands	Rotterdam	Bart J.A. Rijnders	PI	Member of PC
Brazil	Brasilia	Andre Moraes Nicola	PI	Member of PC
India	New Delhi	Aparna Mukherjee	PI	Member of PC
Belgium	Leuven	Geert Meyfroidt	PI	Member of PC
Belgium	Leuven	Timothy Devos	Co-Investigator	Member of PC

© 2022 Troxel AB et al. *JAMA Network Open.*

CCP Committee

Country/RCT	Location	Name	Role on RCT	Role on COMPILE
USA/CONTAIN	NYC Einstein	Liise-anne Pirofski	PI	Chair of CPAC
USA/UPenn	Philadelphia	John Younger	Chair DSMB	Member of CPAC
Brazil	Brasilia	Andre Moraes Nicola	PI	Member of CPAC
USA/COMPILE	Washington University, St. Louis	Jeffrey Henderson	Consultant	Member of CPAC
Spain	Madrid	José Alcami*	Co-Investigator	Member of CPAC
Netherlands	Rotterdam	Corine Geurtsvankessel	Co-Investigator	Member of CPAC

© 2022 Troxel AB et al. *JAMA Network Open.*

Statisticians Committee

a. Blinded

Country/RCT	Location	Name	Role on RCT	Role on COMPILE
USA/CONTAIN	NYU	Keith Goldfeld	Study Statistician	Chair of BSSC
COMPILE	NYU	Andrea Troxel	PI COMPILE	Member of BSSC

b. Unblinded

Country/RCT	Location	Name	Role on RCT	Role on COMPILE
USA/COMPILE	NYU	Eva Petkova		Unblinded [illegible]
USA/COMPILE	NYU	Mengling Liu		Unblinded [illegible]

© 2022 Troxel AB et al. *JAMA Network Open.*

cDSMB

Country/RCT	Location	Name	Role on RCT	Role on COMPILE
USA/CONTAIN	Boston	Elliott Antman	DSMB Chair	Chair of cDSMB
USA/COMPILE	NYU	Eva Petkova	DSMB Statistician	Member of cDSMB
Spain	Madrid	Arantxa Sancho-Lopez	DSMB Chair	Member of cDSMB
Spain	Madrid	Aitor Perez		Member of cDSMB
USA/UCSF	San Francisco	John Szumowski	DSMB Chair	Member of cDSMB
USA/UCSF	San Francisco	David Glidden	Unblinded Statistician	Member of cDSMB
USA/UPenn	Philadelphia	John Younger	DSMB Chair	Member of cDSMB
USA/UPenn	Philadelphia	Pamela Shaw	Unblinded Statistician	Member of cDSMB
Brazil	Brasilia	Andre Siqueira		Member of cDSMB
The Netherlands	Rotterdam	Erik Boersma	DSMB Chair	Member of cDSMB
The Netherlands	Rotterdam	Greg Papageorgiou	Unblinded Statistician	Member of cDSMB
India	New Delhi	L. Jeyaseelan	Unblinded statistician	Member of cDSMB
India`	New Delhi	Suman Pramanik	DSMB member	Member of cDSMB
Belgium	Leuven	Emmanuel Lesaffre	DSMB statistician	Member of cDSMB
Belgium	Leuven	Severine Vermeire	DSMB Chair	Member of cDSMB
USA/CONTAIN	NYU	Mengling Liu	Unblinded statistician	Member of cDSMB
USA	NYU	David Wallach	Science and Research Officer	Non-voting member of cDSMB
USA/COMPILE	NYU	Danni Wu		Non-voting member of cDSMB
USA/COMPILE	NYU	Yinxiang Wu		Non-voting member of cDSMB
USA/COMPILE	NYU	Hyung Park		Non-voting member of cDSMB

© 2022 Troxel AB et al. *JAMA Network Open.*

Data Analysis Team

Country/RCT	Location	Name	Role on RCT	Role on COMPILE
USA/COMPILE	NYU	Eva Petkova		Unblinded Statistician
USA/COMPILE	NYU	Mengling Liu		Unblinded Statistician
USA/COMPILE	NYU	Yinxiang Wu		Unblinded Statistician
USA/COMPILE	NYU	Danni Wu		Unblinded Statistician
USA/COMPILE	NYU	Hyung Park		Unblinded Statistician

© 2022 Troxel AB et al. *JAMA Network Open.*

eAppendix 3. Governance Documents

cDSMB Charter (begins on next page)

© 2022 Troxel AB et al. *JAMA Network Open.*

Collective DSMB for the COMPILE: A Prospective Individual Patient Data Meta-Analysis Study

CHARTER

This charter defines the roles and responsibilities of the collective Data and Safety Monitoring Board (cDSMB) for "COMPILE: A prospective individual patient data meta-analysis" study of the Continuous Monitoring of Pooled International Trials of Convalescent Plasma for COVID-19 Hospitalized Patients (COMPILE) Consortium. The goal of the "COMPILE: A prospective individual patient data meta-analysis" study is to compile the existing individual patient data (IPD) from the collaborating RCTs, to regularly update the pooled data set with new de-identified IPD from the ongoing RCTs, and to continuously monitor the accumulating data until sufficient evidence exists to enable reliable and convincing conclusions regarding the safety and efficacy (or harm) of CP in the target population.

This DSMB, referred to as the cDSMB, will consist of representatives from all DSMBs monitoring the randomized clinical trials (RCTs) that are part of the COMPILE Consortium. The cDSMB will be responsible for monitoring the data submitted by the collaborating RCTs and the accumulating data from the ongoing collaborating RCTs. Given the rapidly evolving nature of the COVID-19 pandemic, the cDSMB may find it helpful to exchange confidential information about evolving findings so as to facilitate decision-making in the face of insufficient sample sizes, a high rate of events, and statistical uncertainty with DSMBs monitoring other RCTs of convalescent plasma (CP) in the target population of hospitalized COVID-19 patients.

The cDSMB will serve in accordance with the guidelines set forth in this charter. The cDSMB members will review and agree to this charter. If amendments to the charter are necessary, the cDSMB reviews and affirms members' agreement with the changes. Their concurrence will be noted in the cDSMB meeting summary and the most updated version of the cDSMB Charter will be considered the document of record from the date of adoption by the cDSMB.

DSMB RESPONSIBILITIES

The cDSMB is responsible for safeguarding the interests of study participants and assessing the safety and efficacy of study procedures and interventions.

Responsibilities include:

- Review and recommend edits to the COMPILE: A prospective individual patient data meta-analysis study protocol and the data and safety monitoring plan prior to the first review of data from that study. Given the time-sensitive nature of the COVID-19 studies, the cDSMB will do their best to provide such input as soon as possible.
- Assist the COMPILE collaborators in protecting the safety of their RCT's participants through review of likely CP-related adverse events.
- Provide input to the COMPILE collaborators on the progress of "COMPILE: A prospective individual patient data meta-analysis study", including periodic assessments of data quality, participant risk versus benefit, and other factors that may affect study outcome.
- Consider factors external to the study when relevant information becomes available, such as scientific or therapeutic developments that may have an impact on the safety of the participants or the ethics of the study.
- Review and recommend subsequent substantive changes to the protocol (after initiation of enrollment), and provide input to the COMPILE collaborators on modification of the study protocol or possible early termination of the study because of attainment of study objectives, safety concerns, or inadequate performance (such as enrollment and retention problems). Minor changes that do not impact patient safety or the assessment of efficacy do not require

© 2022 Troxel AB et al. *JAMA Network Open.*

independent review by the cDSMB. Instead they will be reported to the cDSMB as a part of ongoing monitoring reports;

- Provide input to the investigator(s) on the potential impact of ancillary studies

To assist the cDSMB in carrying out its responsibilities, communications of a confidential nature will occur over a secure server. In addition, a secure website for cDSMB members and unblinded statisticians will be established in the password- protected COMPILE website for collaborators, where the array of "COMPILE: A prospective individual patient data meta-analysis" study-related documents, for which the DSMB is providing oversight, can be accessed.

MEMBERSHIP

Members of the cDSMB, including the Chair, appointed by the Steering Committee (SC) of the COMPILE Consortium and is an independent, multidisciplinary group consisting of clinical investigators, ethicists, and biostatisticians who are experts in clinical trial methodology and conduct. Members will agree to serve on the cDSMB for a period of one year. If members become unable to complete this term of service they will bring this to the attention of the cDSMB Chair and the COMPILE SC who will devise a plan for naming a replacement. Membership is designed to ensure that the DSMB that is/was monitoring each of the collaborating RCTs is represented in the COMPILE cDSMB, and should there be a conflict of interest for any of the voting members, including the Chair, another voting member may lead the meeting instead. The cDSMB has the authority to seek input from non-member experts as needed.

Voting Members:
Chair: Elliott Antman, MD
Alison Bateman-House, PhD
Eric Boersma, PhD
David Glidden, PhD
L. Jeyaseelan, PhD
Emmanuel Lesaffre, PhD
Mengling Liu, PhD
Grigorios Papageorgiou, PhD
Aitor Perez, PhD
Eva Petkova, PhD
Suman Pramanik, MD
Arantxa Sancho-Lopez,
André Siqueira, MD
John Szumowski, MD
Séverine Vermeire,
John Younger, MD

Non-Voting:
Data analysts
Yinxiang Wu, MS
Danni Wu, MS

© 2022 Troxel AB et al. *JAMA Network Open.*

DSMB Coordinators:

David Wallach
Katherine Armstrong

CONFLICT OF INTEREST

All COMPILE DSMB members are expected to disclose potential conflicts of interest to the Chair. Any member with a conflict of interest on a study will be recused from the study-specific discussion.

COMPILE DSMB MEETINGS

COMPILE DSMB meetings are usually held by remote connections. In special circumstances, the meetings may also be conducted by email. Meetings shall be closed to the public because discussions may address confidential participant data. A quorum consisting of at least 1 member of 80% of the collaborating RCTs is required for the meeting to proceed. Due to the wide diversity in time zones of cDSMB members, it is expected that not all members will be able to be regularly present. Members who cannot attend the meetings will be asked to send comments in advance of the meetings. Ideally, recommendations from the cDSMB should be a result of cDSMB consensus. If consensus cannot be achieved, the cDSMB members should formally vote on all recommendations to be submitted to the COMPILE Steering Committee (SC). To vote, a cDSMB member must either be present (via teleconference) at the convened meeting or must have reviewed the relevant report and minutes of the cDSMB meeting at the time in question. While a simple majority vote of members passes a proposal, motion, or recommendation, the cDSMB voting results should also be reported.

Emergency meetings

An emergency meeting of the COMPILE DSMB may be called at any time by the DSMB Chair, should questions of participant safety or COMPILE study integrity arise.
All reviews are based on data provided to the COMPILE DSMB by the COMPILE unblinded statisticians.

Meeting Format

COMPILE DSMB meetings are held initially approximately every once a month to allow for continuous monitoring of accumulating data. The cDSMB has the authority to adjust the pre-specified meeting frequency. The cDSMB coordinator will be responsible for creating and maintaining the meeting schedule, as well as drafting the agenda for cDSMB meetings. The cDSMB coordinator will ensure that data have been submitted sufficiently in advance and the reports generated prior to the meeting. Meeting agendas will be drafted 1-3 days before each meeting, and the cDSMB coordinator will distribute the agenda and meeting materials to all cDSMB members at that time. A final agenda will be distributed the day of the meeting if any changes are needed. Reports will be drafted and shared with the cDSMB in advance of each meeting when practical.
The cDSMB meetings will be divided into two sessions. This format may be modified as needed.

Open Session

Due to the dispersed locations and time zones of the international collaborating RCTs, the COMPILE Steering Committee and other members of the collaborating RCTs will be invited to the open sessions of cDSMB only in exceptional circumstances. Issues discussed in the open session will include the conduct and progress of the study, including data accumulation, recruitment of new participants in the ongoing RCTs, data quality, general adherence and toxicity issues, compliance with the COMPILE protocol, and any other logistical matters that may affect either the conduct or outcome of the COMPILE study. Proposed protocol amendments will also be presented in this session.

© 2022 Troxel AB et al. *JAMA Network Open.*

Closed Session
The closed session is attended only by cDSMB members without conflicts of interest. At the closed sessions the treatment groups will not be blinded. Focus will be on monitoring for efficacy and harm, safety concerns, concerns with compliance with data transmission by the individual collaborating RCTs and review of any pending action items. The cDSMB will make a recommendation for either continuation or termination of the COMPILE study and will communicate their recommendations to the COMPILE Steering Committee. If questions arise during the members' discussion, the COMPILE ES might be called in to respond, after which the Closed session can proceed without external members.

Early termination

Termination may be suggested by the DSMB at any time. Reasons for early termination include:
- Accumulating evidence of safety problems from the CP treatment (e.g., stopping guidelines for safety are met; large number of serious and related AEs that are not balanced by evidence of benefit)
- Accumulated evidence indicating efficacy of CP (guidelines for stopping for efficacy are met)
- Accumulated evidence indicating harm of CP (guidelines for stopping for harm are met)
- Logistical or data quality problems so severe that the scientific integrity of the data is in doubt, and correction is not feasible

REPORTS TO THE cDSMB

The unblinded data analysis team will prepare summary reports and tables using the reporting templates requested by the cDSMB. The cDSMB should discuss at the first or subsequent meetings what data they wish to review and how it should be presented. The reports will be distributed to the cDSMB with the agenda in advance of each meeting. Requested most recent updated summaries might be distributed during the cDSMB meeting. These reports shall be provided by secure email, or by access to the password-protected COMPILE website.

Open Data reports will include overall, by RCT and by a recruitment site within an RCT (if multiple sites are involved in a given RCT):
- Rolling enrollment reports
- Summaries of likely CP-related AEs and SAEs
- Participant status reports (WHO score at day 14±1 and day 28±2)
- Participant is at levels 7-10 of the WHO score, at day 14±1 and day 28±2
- Mortality at day 14±1 and day 28±2

Closed Data reports will include by treatment group
- Summaries of likely transfusion-related AEs and SAEs and results from the logistic regression model for presence/absence of AE and SAEs (see details below)
- Results from analytic models specified in Table 1

List of prespecified analyses to be performed and reviewed by cDSMB at each meeting. Details about the models are given in the Statistical analysis plan.

Analysis			Time since Rand			Interim	Interim	Interactions

© 2022 Troxel AB et al. *JAMA Network Open.*

	Category	Description	omiza tion	Treatment	Covariate Adjustment	Analysis Rule # 1	Analysis Rule #2	Sex	Age	Symp dur.	WHO base
1	**Primary**	CPR[1] WHO score	D14	CP vs Control(3)	Parsimonious[2]	Both primary endpoints are satisfied	At least one of the primary is satisfied and at least one of the secondary is satisfied	a	b	c	d
2	**Primary**	WHO 7-10[3]	D14	CP vs Control(3)	Parsimonious			a	b	c	d
3	Secondary	CPR WHO score	D14	CP vs Control(3)	Expanded[4]			a	b	c	d
4	Secondary	WHO 7-10	D14	CP vs Control(3)	Expanded			a	b	c	d
5	Secondary	CPR WHO score	D28	CP vs Control(3)	Expanded			a	b	c	d
6	Secondary	WHO 7-10	D28	CP vs Control(3)	Expanded			a	b	c	d
7	Tertiary	Total Mortality[3]	D14	CP vs Control(3)	Expanded			a	b	c	d
8	Tertiary	Total Mortality[3]	D28	CP vs Control(3)	Expanded			a	b	c	d
9	Tertiary	Time to discharge		CP vs Control(3)	Expanded			a	b	c	d
Effect of quantity of CP units (0, 1, 2, …) and levels of antibodies (Ab) in the CP (none, low, not-low)											
10	Tertiary	CPR WHO score	D14	# CP units/Ab level	Expanded			a	b	c	d
11	Tertiary	WHO 7-10[3]	D14	# CP units/Ab level	expended			a	b	c	d
12	Tertiary	CPR WHO score	D28	# CP units/Ab level	Expanded			a	b	c	d
13	Tertiary	WHO 7-10[3]	D28	# CP units/Ab level	Expanded			a	b	c	d
14	Tertiary	Total Mortality[3]	D14	# CP units/Ab level	Expanded			a	b	c	d
15	Tertiary	Total Mortality[3]	D28	# CP units/Ab level	Expanded			a	b	c	d
16	Tertiary	Time to discharge		# CP units/Ab level	Expanded			a	b	c	d

[1] CO=Cumulative odds model (will be used to model the WHO 11-points ordinal outcome of patient clinical status)
[2] Parsimonious covariate adjustment = age, sex, WHO status at baseline, duration of symptoms before randomization, quarter of enrollment
[3] Logistic regression model for binary outcome
[4] Expanded covariate adjustment = parsimonious & medical history, concomitant medication at randomization, blood type

DOCUMENTATION OF cDSMB MEETINGS

The cDSMB coordinator will prepare a formal memo containing the cDSMB's decision and recommendations regarding study continuation 1 business day after each meeting. Once approved and signed by the cDSMB Chair, the memo will be sent to the Chair of the SC to share with SC members. It is the responsibility of the PIs of individual RCTs to distribute the summary to their study teams.

The cDSMB coordinator will also prepare formal minutes to document all DSMB meeting discussions. Minutes will be approved by the cDSMB prior to being archived.

MONITORING PLAN

Details about the analyses and models on which the stopping guidelines are based are given in the COMPILE Statistical Analysis Plan (SAP). The cDMSB charter should always be read the relation to the latest version of the SAP.
The text below only presents highlights of the models.

Monitoring for efficacy and harm

© 2022 Troxel AB et al. *JAMA Network Open.*

WHO score at 14 days

The first primary efficacy/harm outcome is the WHO 11 points clinical status scale. The analysis will be a cumulative odds model for the ordinal WHO score at 14 days (±1 day). If Y is the WHO 11-point scale, (Y=0, ..., 10), let

$$q_{\$} = P(Y = y),\ \ y = 0, \dots, 10,\ \ \mathop{/}\limits_{\$21}^{01} q_{\$} = 1$$

and let (1)

$$p_{\$} = P(Y \geq y) = \mathop{/}\limits_{52\$}^{01} q_{5},\quad y = 1, \dots, 10.$$

Assume that data from K RCTs are available. Only subjects randomized to the CP or the control condition from the studies will be used, i.e., patients randomized to other active treatments in an RCT with more than two treatment arms will be ignored. There are n_{8} subjects in the kth trial, $k = 1, \dots, K$. Denote the outcome for the i-th patient from the kth trial on the 11-point WHO ordinal COVID-19 scale at day 14 by $Y_{8<} = y$, $y = 0, \dots, 10$, and that patient's baseline covariates (a vector of length m) by $\boldsymbol{x}_{8<}$. Also, denote by $p_{8<\$}$, the respective probabilities in (1) for the ith subject in the kth RCT.

Because all trials will have the intervention arm of convalescent plasma but may have different control arms, we propose a statistical model with the following notation for treatment effect modeling. Let $I^{@ABC}$ be a 3-dimensional indicator vector for the treatment and let C denote the control treatment variable: standard of care, $C = 0$; non-convalescent plasma, $C = 1$; saline/LR with coloring, $C = 2$. Then

$I^{@ABC}$ = (1,0,0) Control treatment $C = 0$ (standard of care)
$I^{@ABC}$ = (0,1,0) Control treatment $C = 1$ (non-convalescent plasma)
$I^{@ABC}$ = (0,0,1) Control treatment $C = 2$ (saline/LR with coloring agent)
$I^{@ABC}$ = (0,0,0) Active treatment (convalescent plasma)

and $I_{8<}^{@ABC}$ will indicate the treatment assignment for subject i in the kth RCT. The corresponding k^{th} trial-specific control- arm effects are denoted by $I^{@ABC}\boldsymbol{\delta}_{8}$, where $\boldsymbol{\delta}_{8} = (\delta_{81}, \delta_{80}, \delta_{8H})'$, and the control effects are denoted by $\boldsymbol{\delta}_{@} = (\delta_{1}, \delta_{0}, \delta_{H})'$.

The following cumulative odds (CO) model for $Y_{8<}$ will be considered:

$$
\begin{aligned}
Y_{8<} &\sim \text{Ordinal multinomial}(\boldsymbol{p}_{8<}),\quad \boldsymbol{p}_{8<} = \{p_{8<\$}\}_{\$}^{01} \\
\text{logit}\text{Y}P(Y_{8<} \geq y)\text{Z} &= \tau_{\$8} + \boldsymbol{\beta}_{8}\boldsymbol{x}_{8<} + \boldsymbol{I}_{8<}^{@ABC}\boldsymbol{\delta}_{8} \\
\alpha &\sim \text{Normal}(0, \sigma = 0.1) \\
\tau_{\$8} &\sim t_{\text{cdefghd}}(\text{df} = 3, 0, \sigma = 8),\qquad \text{monotone within } k \\
\boldsymbol{\beta}_{8} &\sim \text{Normal}(\boldsymbol{0}, \Sigma = 2.5^{H} I_{\mathbf{r}\times\mathbf{r}}) \\
\boldsymbol{I}_{8}^{@ABC}\delta_{8} &\sim \text{Normal}(\delta_{@}, \sigma = \eta),\quad c = 0,1,2 \text{ for the 3 control conditions} \qquad (2) \\
\eta &\sim t_{\text{cdefghd}}(\text{df} = 3, 0, 0.25) \\
\delta_{@} &\sim \text{Normal}(-\Delta_{\{|}, \sigma = 0.5),\ c = 0,1,2 \\
-\Delta_{\{|} &\sim \text{Normal}(0, \sigma = 0.354)
\end{aligned}
$$

The proposed model (2) conceptualizes the three control conditions as three treatments to be compared against the reference condition of convalescent plasma (CP). Being the reference treatment, the log odds defined from the cumulative probabilities of CP arm are estimated by α_{yk} from (2), which corresponds to the kth study's intercept associated with level y on the ordinal WHO outcome $Y = y$, $y = 1, \dots, 10$. We impose a very skeptical hyper-prior for these trial-specific CP effects. All $\tau_{\$8}$, $y = 1, \dots, 10$ satisfy the monotonicity requirements for the intercepts of the proportional odds model. We impose a hyper-prior distribution for three study-specific control treatment effects, which come from the same distribution with the overall treatment effect Δ being the parameter of primary interest. We take $-\Delta_{\{|}$ as the

© 2022 Troxel AB et al. *JAMA Network Open.*

mean of the distribution to which $\delta_{@}$ belongs so that $\Delta_{\{|}$ will correspond to the difference of log-odds for CP and log-odds for control, rather than control minus CP.

WHO score greater than or equal to 7 at 14 days

The second primary efficacy/harm outcome is an indicator whether a patient's WHO 11-points scale was 7 or higher, at day 14 ($\pm$1 day). Bayesian logistic regression model will be used to model this outcome:

© 2022 Troxel AB et al. *JAMA Network Open.*

$$W_{8<} \sim \text{Bernoulli}\,(p_{8<})$$
$$\text{logit}\,\Upsilon P(W_{8<} = 1)Z == \tau_k + \boldsymbol{\beta}_k \boldsymbol{x}_{ki} + \boldsymbol{I}_{ki}^{ctrl}\boldsymbol{\delta}_k$$
$$\tau_8 \sim t_{\text{cde}\mathbf{f}\text{ghd}}(\text{df} = 3{,}0, \sigma = 8),$$
$$\boldsymbol{\beta}_8 \sim \text{Normal}(\mathbf{0}, \Sigma = 2.5^{\text{H}} I_{\mathbf{r}\times\mathbf{r}})$$
$$\boldsymbol{I}_8^{@\text{ABC}}\delta_8 \sim \text{Normal}(\delta_@, \sigma = \eta), \quad c = 0,1,2 \text{ for the 3 control conditions} \qquad (3)$$
$$\eta \sim t_{\text{cde}\mathbf{f}\text{ghd}}(\text{df} = 3, 0, 0.25)$$
$$\delta_@ \sim \text{Normal}(-\Delta_{\text{Ñ}}, \sigma = 0.5), \; c = 0,1,2$$
$$-\Delta_{\text{Ñ}} \sim \text{Normal}(0, \sigma = 0.354)$$

Similar to model (2) for the ordinal WHO scale, we take $-\Delta_{\text{Ñ}}$ as the mean of the distribution to which $\delta_@$ belongs so that $\Delta_{\text{Ñ}}$ will correspond to the difference of log-odds for CP and log-odds for control, rather than control minus CP.

Stopping for efficacy: Rule 1

$P(OR_{@\text{á}} < 1) = P\Upsilon e^{\text{äàà}} < 1Z = P(\Delta_{\{|} < 0) \geq 0.95$ **and** $P(OR_{@\text{á}} < 0.8) = P\Upsilon e^{\text{äàà}} < 0.8Z = P(\Delta_{\{|} < -0.223) \geq 0.5$

and

$P(OR_{\text{C}} < 1) = P\Upsilon e^{\text{ä}_{\text{é}}} < 1Z = P(\Delta_{\text{Ñ}} < 0) \geq 0.95$ **and** $P(OR_{\text{C}} < 0.8) = P\Upsilon e^{\text{ä}_{\text{é}}} < 0.8Z = P(\Delta_{\text{Ñ}} < -0.223) \geq 0.5$

Stopping for efficacy: Rule 2

For $e^{\text{ä}}$ denoting the efficacy/harm OR parameter in any of the primary and secondary outcome listed in Table 1

$P(OR < 1) = P\Upsilon e^{\text{ä}} < 1Z = P(\Delta < 0) \geq 0.95$ **and** $P(OR < 0.8) = P\Upsilon e^{\text{ä}} < 0.8Z = P(\Delta < -0.223) \geq 0.5$

should be satisfied for one primary and for at least 2 of the secondary outcomes.

Stopping for harm

$P(OR_{@\text{á}} > 1) = P\Upsilon e^{\text{äàà}} > 1Z = P(\Delta_{\{|} > 0) \geq 0.80$ **or** $P(OR_{\text{C}} > 1) = P\Upsilon e^{\text{ä}_{\text{é}}} > 1Z = P(\Delta_{\text{Ñ}} > 0) \geq 0.80.$

Monitoring for safety

The model
We propose monitoring for safety based on adverse events related to the transfusion of plasma. Specifically, we will compare the CP and control conditions with respect to the proportion of patients who experienced at least one of the adverse events in Section 5.2, E: TRALI, TACO, or any other transfusion reaction.

© 2022 Troxel AB et al. *JAMA Network Open.*

The presence/absence of transfusion related adverse events is a binary outcome, so we will be using logistic regression models. Let $Z_{8<}$ be an indicator that the i-th subject in the kth RCT experiencing a transfusion-related event. Similar to the consideration in Section 10.1, to accommodate the three different control conditions, we conceptualize the three different control conditions as three treatments to be compared against the reference condition (CP). The effects of the control

© 2022 Troxel AB et al. *JAMA Network Open.*

conditions $C = c$, $c \in \{0,1,2\}$ on the transfusion-related adverse events, will be denoted by $\theta_{@}$, $c \in \{0,1,2\}$ and we will impose a hyper-prior distribution for those three estimates of control effects, that are coming from the same distribution. Being the reference treatment, the log odds of having the transfusion-related event in the CP arm is estimated by γ_{8} from (3), which corresponds to the kth trial's intercept. We impose a very skeptical hyper-prior for these site-specific CP effects.

The following logistic regression model will be used to model Z:

$$
\begin{aligned}
Z_{8<} &\sim \text{Binomial}(\boldsymbol{r}_{8<}), \quad 0 < \boldsymbol{r}_{8<} < \\
1 \ \text{logit} Y P(Z_{8<}) Z &= \gamma_{8} + \boldsymbol{\lambda}_{8} \boldsymbol{x}_{8<} + \boldsymbol{I}^{@\text{ABC}} \theta_{8} \\
\gamma_{8} &\sim t_{\text{cdefghd}}(\text{df} = 3, 0, \sigma = 2.3) \\
\boldsymbol{\lambda}_{8} &\sim \text{Normal}(\boldsymbol{0}, \Sigma = 10^{\text{H}} \boldsymbol{I}_{\boldsymbol{m} \times \boldsymbol{m}}) \\
\boldsymbol{I}_{8}^{@\text{ABC}} \theta_{8} &\sim \text{Normal}(\theta_{@}, \eta^{\text{H}}), \quad c = 0,1,2 \text{ for the 3 control conditions} \qquad (3) \\
\eta &\sim \text{Cauchy}(0, 2.5) \\
\theta_{@} &\sim \text{Normal}(-\Theta, \sigma = 0.1), \ c = 0,1,2 \\
-\Theta &\sim \text{Normal}(0, \sigma = 5)
\end{aligned}
$$

We take $-\Theta$ as the mean of the distribution to which θ belongs, so that Θ will correspond to the difference of log-odds for CP and log-odds for control, rather than control minus CP.

Stopping for safety

$$P(e^{\hat{u}} > 1) = P(\Theta > 0) > 0.75.$$

CONTENT of cDSMB REPORTS

A: Cumulative enrollment

This will be presented across all RCTs, by RCT and by recruitment site within RCT

B: Baseline characteristics of all participants

The information below will be presented in several tables:

- All patients
- By RCT and by recruitment site within RCT
- By treatment

C: AEs

Individual AEs summarized by Category
This information will be presented in several tables:

- All patients
- By Site
- By treatment

D: WHO Ordinal Score for Clinical Outcomes: These outcomes will be presented in the following format

Major assessment times are pre-treatment (baseline), day 14 (±1), day 28 (± 2). The information will be presented in several tables:

- All patients

© 2022 Troxel AB et al. *JAMA Network Open.*

- By site
- By treatment

© 2022 Troxel AB et al. *JAMA Network Open.*

Patient State day 14±1 (30±2)	core	Descriptor	WHO score at baseline					
			Level 4	Level 5	Level 6	Level 7	Level 8	Level 9
		Total N (%)						
Uninfected	0	Uninfected; no viral RNA detected N (%)						
Ambulatory	1	Asymptomatic; viral RNA detected N (%)						
	2	Symptomatic: Independent N (%)						
	3	Symptomatic: assistance needed N (%)						
Hospitalized: Mild disease	4	Hospitalized; no oxygen therapy N (%)						
	5	Hospitalized; oxygen by mask or nasal prongs N (%)						
Hospitalized: Severe disease	6	Hospitalized; oxygen by NIV or High flow N (%)						
	7	Intubation & Mechanical ventilation, pO2/FIO2 >/= 150 or SpO2/FIO2 >/=200 N (%)						
	8	Mechanical ventilation pO2/FIO2 < 150 (SpO2/FIO2 <200) or vasopressors N (%)						
	9	Mechanical ventilation pO2/FIO2 < 150 and vasopressors, dialysis or ECMO N (%)						
Death	10	Dead N (%)						

Tables, Listings, and Figures

The following are shells of the tables we expect to produce for the interim and final analyses.

Table 0. Participating studies

			Final sample size	
Title	Participating sites	Control condition	Control	CP
XX	Site 1	Standard of care	m1	n1
	Site 2			
	Site 3			
YY	Site 1	Saline	m2	n2
ZZ	Site 1	Non-convalescent plasma	m3	n3
…	…	…	…	…
Total			mm	nn

© 2022 Troxel AB et al. *JAMA Network Open.*

Table 1. Baseline demographic and clinical characteristics		
Variable	Control Group (total # = mm)	CP Group (total # = nn)
Age (median, IQR)		
Female sex (n, %)		
Enrollment quarter		
January -March 2020		
April-June 2020		
July-September 2020		
October-December 2020		
January -March 2021		
April-June 2021		
Baseline WHO severity		
4 (n, %)		
5 (n, %)		
6 (n, %)		
7 (n, %)		
8 (n, %)		
9 (n, %)		
Blood group		
O (n, %)		
A (n, %)		
B (n, %)		
AB (n, %)		
Not available (n, %)		
Days since symptoms onset at time of enrollment		
0-3		
4-6		
7-10		
11-14		
More than 14		
Days since COVID-19 diagnosis at enrollment (median, IQR)		
Status at enrollment		
Outpatient (n, %)		
Hospitalized, non-ICU (n, %)		
Hospitalized, ICU not on mechanical ventilation (n, %)		
Hospitalized, ICU on mechanical ventilation (n, %)		
Time of treatment in days since enrollment (median, IQR)		
History of diabetes (all types) (n, %)		
History of pulmonary disease (all types) (n, %)		
History of cardiovascular disease (all types) (n, %)		

© 2022 Troxel AB et al. *JAMA Network Open.*

**Table 2. Primary outcome analysis**						
	WHO score at day 14 (±1)			WHO score at day 14 (±1) > or =7		
	OR	Prob(OR<1)	Prob(OR<0.8)	OR	Prob(OR<1)	Prob(OR<0.8)
**Control: Standard of care**						
RCT 1						
RCT 2						
…						
Sub-aggregate estimate						
**Control: Saline**						
RCT 5						
RCT 6						
…						
Sub-aggregate estimate						
**Control: Non-convalescent plasma**						
RCT 9						
RCT 10						
…						
Sub-aggregate estimate						
**Meta-analysis aggregate estimate**						

**Monitoring history**						
Interim analysis sequence			WHO score at day 14 (±1)		WHO score at day 14 (±1) > or =7	
	Date	N control & CP	Prob(OR<1)	Prob(OR<0.8)	Prob(OR<1)	Prob(OR<0.8)
1						
2						
3						
…						

© 2022 Troxel AB et al. *JAMA Network Open.*

Main governance document

The COMPILE Consortium will be governed by the Steering Committee (SC), which will consist of at least one member of each RCT. Typically, that member would be the PI of the RCT, but the PI might appoint another member of the RCT to replace her/him. The SC will be chaired by the PI of the COMPILE study, Dr. Andrea Troxel. The Chair of the SC, who is responsible for the overall conduct of the COMPILE study, will be assisted in this by members of the SC. The SC will ensure that updated minimal data sets (MDS) of de-identified individual patient data (IPD) are transferred to the COMPILE database in a timely manner. The SC will be responsible for establishing sub-committees necessary for the proper conduct of the COMPILE study. For example, the SC will establish and facilitate the work of a sub-committee on publications, a sub-committee of blinded statisticians and a sub-committee on convalescent plasma antibodies. Other sub-committees will be established as needed.

The collective DSMB (cDSMB) for the COMPILE study will consist of the Chairs and the statisticians from DSMBs of all collaborating RCTs. Dr. Elliott Antman, Chair of the DSMB for the CONTAIN COVID-19 study (with recruiting hubs at NYU, Einstein University, Yale University, University of Miami and the University of Texas in Houston) will chair the cDSMB. The cDSMB will review interim analyses results on a regular basis, every 2 weeks. The cDSMB will be assisted by a data manager team and a team of unblinded statisticians who will perform the interim analyses according to the statistical analysis monitoring plan and will prepare the cDSMB reports.

Schematic representation of the COMPILE governance is presented in Figure 1. The mandates and the membership of all committees, sub-committees and assisting teams are listed below. The committees' and sub-committees' membership will be updated as new RCTs join the COMPILE Consortium. The mandates can also be updates as needed.

© 2022 Troxel AB et al. *JAMA Network Open.*

COMPILE Governance

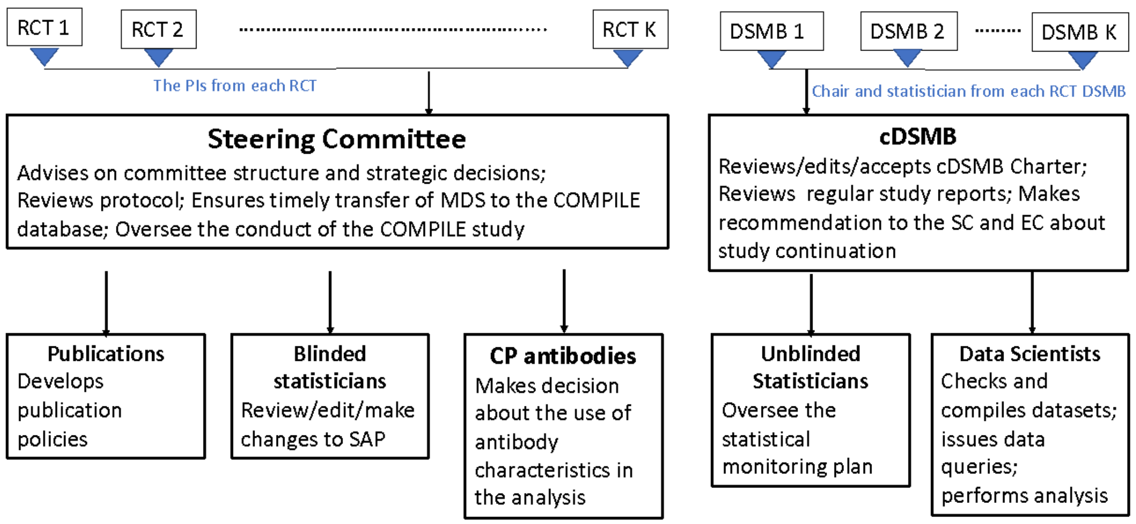

8

Template consortium agreement

Continuous Monitoring of Pooled International Trials
of Convalescent Plasma for COVID-19 Hospitalized Patients
(COMPILE)

CONSORTIUM AGREEMENT

THIS AGREEMENT, effective as of the date of the last signature below, is by and between NYU Grossman School of Medicine, an administrative unit of New York University ("NYU"), and [_____________] (the "Consortium Investigator"), for the purpose of the Consortium Investigator's participation in the Continuous Monitoring of Pooled International Trials of Convalescent Plasma for Covid-19 Hospitalized Patients (COMPILE).

A. GENERAL OVERVIEW

1) The COMPILE Consortium is an international pooling project designed to combine patient level data from multiple randomized controlled trials of convalescent plasma in hospitalized patients with COVID-19 ("Qualifying Trials").
2) The goal of the COMPILE Consortium is to provide robust data to inform interim analyses and potentially to arrive at a collective scientifically justified recommendation regarding the efficacy or lack of efficacy of convalescent plasma sooner than would be possible if real-time pooling did not occur.
3) The COMPILE Consortium will assemble datasets from multiple Qualifying Trials not originally configured as a network into an aggregate de-identified individual patient data set from multiple Qualifying Trials (the "COMPILE Consortium Data Set") securely maintained by NYU and managed under the direction of Andrea B. Troxel, ScD (the "NYU Investigator").
4) The COMPILE Data Set will help researchers determine more quickly if convalescent plasma is effective against COVID-19. An answer to this question is urgently needed since, as of the date of establishment of COMPILE Consortium, there is only one therapeutic with modest efficacy against COVID-19, while the pandemic continues to spread unabated in the United States and other parts of the world.
5) Investigators conducting Qualifying Trials may participate in the COMPILE Consortium by entering into this Agreement.

B. CONSORTIUM INVESTIGATOR. The Consortium Investigator is a principal investigator of [Name of Trial], a Qualifying Trial, and is willing to transmit to NYU de-identified individual patient data from the Qualifying Trial for inclusion by NYU in the COMPILE Consortium Data Set pursuant to the Continuous Monitoring of Pooled International Trials of Convalescent Plasma for Covid-19 Hospitalized Patients Terms & Conditions set forth in Schedule 1 to this Agreement. In the event the Consortium Investigator needs approvals to transmit such data to NYU hereunder, the Consortium Investigator will work in good faith to obtain the approval of his or her institution (the "Consortium Institution") to the terms and conditions of this Agreement.

C. ENTIRE AGREEMENT. This Agreement, including Schedule 1, constitutes the entire agreement between the parties with respect to the subject matter; supersedes and replaces all prior agreements, oral or written, between the parties relating to the subject matter; and may not be modified or otherwise changed in any manner other than as a set forth in Schedule 1. This Agreement may be executed in counterparts, each of which shall be deemed an original but all of which together shall constitute one and the same instrument.

© 2022 Troxel AB et al. *JAMA Network Open.*

IN WITNESS WHEREOF, the parties hereto have caused this Agreement to be executed as of the date first written above.

**NYU GROSSMAN SCHOOL OF MEDICINE**	**Consortium Investigator**
______________________________	______________________________
Name: Samantha Ebel<br>Title: Associate Director-OSR Contracts<br><br>Date:<br><br>Accepted and Agreed by the NYU Investigator:	Name:<br><br>Date:<br><br>IF REQUIRED:<br>Accepted and Agreed by Consortium Institution:
______________________________	______________________________
Andrea B. Troxel, ScD	Name:<br>Title:<br><br>Date:

© 2022 Troxel AB et al. *JAMA Network Open.*

SCHEDULE 1

Continuous Monitoring of Pooled International Trials of Convalescent Plasma for COVID-19 Hospitalized Patients (COMPILE)

TERMS & CONDITIONS

Capitalized terms not defined herein shall have the meaning set forth in the Agreement to which these Terms & Conditions are attached.

A. CONSORTIUM INVESTIGATOR RESPONSIBILITIES

1) The Consortium Investigator represents that he or she is a principal investigator of a Qualifying Trial, that a copy of the protocol and informed consent form for the Qualifying Trial has been provided to NYU, and that he or she has authority to transmit to NYU de-identified individual patient data (IPD) from the Qualifying Trial for inclusion in the COMPILE Consortium Data Set and/or has obtained written approval from his or her Consortium Institution to enter into this Agreement.
2) The Consortium Investigator agrees to submit, and/or to work with the Consortium Institution to submit, to NYU de-identified individual patient data (IPD) from the Qualifying Trial on preselected characteristics of hospitalized patients (the "Qualifying Trial Data") through the data submission method described in Section C below. Submissions will be made approximately every two weeks for inclusion in the COMPILE Consortium Data Set.
3) The Consortium Investigator shall ensure that the Qualifying Trial Data submitted to NYU has been collected with and is provided in accordance with all applicable privacy rules, including but not limited to the US' Health Insurance Portability and Accountability Act (HIPAA) or the European Union's General Data Protection Regulation (GDPR). All submitted Qualifying Trial Data will be submitted without identifiers that would allow NYU or any other recipient to link the data to any patient.
4) The Consortium Investigator (or designee(s)) may participate in periodic conference calls on COMPILE Consortium related matters.
5) The Consortium Investigator acknowledges that any submitted Qualifying Trial Data may be reviewed for accuracy, completeness, and data quality by NYU. The data management team at NYU will provide guidance to ensure high quality data standards.

B. OWNERSHIP AND SAFEGUARDING OF QUALIFYING TRIAL DATA

1) The Consortium Investigator shall retain ownership of any rights he or she may have to any Qualifying Trial Data.
2) Neither NYU nor any other member of the COMPILE Consortium obtains any rights to the Qualifying Trial Data other than as set forth herein.
3) Except as authorized under this Agreement or otherwise required by law, NYU agrees to retain control over the Qualifying Trial Data it receives and shall not disclose, release, sell, rent, lease, loan, or otherwise grant access to the Qualifying Trial Data to any third party, without the prior written consent of the Consortium Investigator.
4) NYU specifically acknowledges its responsibility to safeguard the confidentiality and security of all Qualifying Trial Data.

C. DATA SUBMISSION

1) The Consortium Investigator will transfer, and/or work with the Consortium Institution to transfer, data files with the Qualifying Trial Data, to NYU's MCIT Research DataCore by secure file transfer protocol (FTP) using the GlobalScape managed file transfer (MFT) solution.
2) The Consortium Investigator/Consortium Institution's designated person will be provided by NYU a unique account requiring valid credentials (user ID and password) to access their corresponding folders in the MFT system to deposit encrypted data files.
3) NYU will set up event transfer rules so that any file a Consortium Investigator/Consortium Institution designated person uploads to their account will immediately transfer to a corresponding folder for the

© 2022 Troxel AB et al. *JAMA Network Open.*

Consortium Investigator/Consortium Institution in the NYU network drive. Following transfer, all data files with Qualifying Trial Data will be deleted from the MFT folder.

4) Submissions and access to COMPILE database may necessitate NYU's issuance to the Consortium Investigator and/or a designated member of their teams of unique identification credentials, a password, verification code, or other electronic signature codes to the COMPILE Application. The Consortium Investigator shall keep, and cause their designees to keep, the ID/Passwords in confidence and use them solely for purposes of this Agreement. The obligation to maintain the ID/Passwords in confidence will survive the term of this Agreement. The Consortium Investigator will notify NYU promptly if an individual provided with an ID/Password is no longer working on the Qualifying Trial; or if the Consortium Investigator becomes aware of any security breach regarding ID/Passwords.

D. DATA POOLING AND ANALYSES AT NYU FOR THE MAIN COMPILE **ANALYSES**

1) NYU will include any submitted Qualifying Trial Data in the COMPILE Consortium Data Set.
2) NYU will maintain the COMPILE Consortium Data in a secure environment. Files of Qualifying Trial Data received at NYU will be pooled into the COMPILE Consortium Data Set, which will be maintained by NYU as a common database within secure Hadoop or MCIT-managed network drive. Routinely scheduled transfers of updated Qualityfing Trial Data from each Consortium Institution/Consortium Investigator will override existing data in the common database.
3) NYU will use the COMPILE Consortium Data Set to conduct pre-specified interim analyses. Snapshots or data dumps from the COMPILE Consortium Data Set database will be transferred by NYU to corresponding read-only folders for the unblinded biostatisticians of an umbrella Data Safety Monitoring Board ("DSMB") (consisting of members of the DSMBs for the Qualifying Trials) solely to conduct such pre-specified interim analyses.
4) Patient-level data in the pooled COMPILE Consortium Data Set will always remain at NYU. NYU will not transfer any data back to any Consortium Investigator/Consortium Institution. Except for the access for analyses as contemplated herein, NYU will not disclose, release, sell, rent, lease, loan, or otherwise grant access to the COMPILE Consortium Data Set to any third party, without the prior written consent of the Consortium Investigator and all Consortium investigators.
5) Programming of operational reports, interim analyses, and final analyses for the main COMPILE project will be performed by NYU employees or agents and the unblinded biostatisticians of NYU's DSMB for COVID-19 related studies (in collaboration with biostatisticians from Qualifying Trials led by Consortium Investigators) in folders within NYU's secure MCIT-managed network drive. Such folders will be accessible only to Consortium Investigator and other Consortium investigators.

E. COMPILE DATA ACCESS FOR ADDITIONAL ANALYSES—MAINTENANCE OF IPD SECURITY

1) Following completion of the main COMPILE project analyses, the Consortium Investigator and other Consortium investigators may request access to the COMPILE Consortium Data Set for additional analyses of scientific interest (substudies).
2) Requests for access may be made through a process defined by the COMPILE Publication Committee and NYU.
3) Decisions regarding all requests will be handled in accordance with the COMPILE Publication Policy noted below.
4) Analyses required by Consortium investigators will be supported by NYU with all programming performed by NYU personnel within the secure network drive.
5) Requests for direct access to the COMPILE Consortium Data Set will require approval by the COMPILE Publication Committee and an additional data use agreement with NYU.
6) If a request is approved and a data use agreement has been signed, NYU will provide to the Consortium Investigator requestor a snapshot or data dump of the common database, which will be created and stored within a virtual device infrastructure (VDI), or virtual machine, residing within NYU's data center. Designated analysts/programmers from the Consortium Institution will be provided with NYU's Kerberos ID and password in order to access the VDI remotely and perform all analyses within the VDI. All statistical applications, analyses datasets and programming will remain within the VDI.

© 2022 Troxel AB et al. *JAMA Network Open.*

F. COMPILE PUBLICATION POLICY

1) The results of the pooling analysis will be published as a joint publication with authorship representation from each Qualifying trial.
2) The COMPILE Publication Committee will oversee the scientific goals of the COMPILE Consortium, reduce duplication, and identify opportunities for collaboration, and will help ensure that all print and online publications and presentations that are based on the COMPILE Data Set are presented accurately with scientific integrity.
3) The COMPILE Publications Committee will be composed of Dr. Liise-anne Pirofski, Dr. Mila Ortigoza, and additional representation from the COMPILE Consortium.
4) All COMPILE Consortium investigators are permitted to develop proposals and analysis plans, and collaborate in the development and publication of research papers based on the COMPILE Consortium Data Set. Collaboration by Consortium investigators is encouraged.
5) Every new proposal using the COMPILE Consortium Data must be submitted to and reviewed and approved by the COMPILE Publication Committee
6) All investigators are expected to adhere to authorship standards described in the International Committee of Medical Journal Editors Uniform Requirements for Manuscripts Submitted to Medical Journals. The requirements state that all persons designated as authors should qualify for authorship and all those who qualify should have participated sufficiently in the work to take public responsibility for appropriate portions of the content. One or more authors should take responsibility for the integrity of the work as a whole from inception to published article.
7) Authorship credit will be assigned in an equitable fashion and will be commensurate with participation and effort on the individual manuscript.
8) All publications will acknowledge the use of the COMPILE Data Set in any publication or presentation made from its use.
9) For the avoidance of any doubt, nothing in this Section F shall limit or restrict the Consortium Investigator from using his or her local data in an abstract, publication or presentation.

G. COSTS AND EXPENSES

Each party shall be responsible for its costs and expenses associated with the COMPILE consortium. Neither the Consortium Investigator nor the Consortium Institution will be paid or be charged in connection with COMPILE Consortium or this Agreement.

H. USE OF NAME

Neither party shall use the other party’s name, trademarks, or other logos in any publicity, advertising, or news release without the prior written approval of an authorized representative of that party. The parties agree that each party may disclose factual information regarding the existence and purpose of the COMPILE Consortium and the other party’s participation in lists of participating Consortium Investigators and Qualifying Trials for other purposes without written permission from the other party provided that any such statement shall accurately and appropriately describe the relationship of the parties and shall not in any manner imply endorsement by the other party whose name is being used.

I. DISCLAIMER; LIMITATION ON LIABILITY

1) Each party shall be responsible for its negligent acts or omissions of its employees, officers, or directors, to the extent allowed by law.
2) Except as is provided in this Agreement, any Qualifying Trial Data delivered by the Consortium Investigator is understood to be provided “AS IS”, and any access to the COMPILE Consortium Data Set made available by NYU is understood to be provided “AS IS”.
3) NO PARTY SHALL BE LIABLE FOR INDIRECT, SPECIAL, CONSEQUENTIAL, PUNITIVE OR EXEMPLARY DAMAGES OF THE OTHER PARTY.

© 2022 Troxel AB et al. *JAMA Network Open.*

J. TERM AND TERMINATION

1) This Agreement shall be effective for so long as the COMPILE Consortium Data Set is maintained by NYU, unless sooner terminated.
2) Either party may terminate this Agreement at any time upon written notice to the other party.
3) Upon termination of this Agreement for any reason, NYU will disable the Consortium Investigator's access to the COMPILE Consortium and COMPILE Consortium Data Set. The Consortium Investigator acknowledges that any data submitted prior to such termination will have been included by NYU in the COMPILE Consortium Data Set and will not be removed in connection with such termination.
4) All use, privacy, confidentiality, security, non-transfer, publication, and other obligations regarding the Qualifying Trial Data, COMPILE Consortium Data Set, and COMPILE Consortium, shall survive expiration or termination of this Agreement.

K. RELATIONSHIP OF THE PARTIES.

NYU and the Consortium Investigator is an independent entity and neither party shall hold itself out to third parties as purporting to act as, or on behalf of, the other party hereto.

© 2022 Troxel AB et al. *JAMA Network Open.*

Data transfer and security

The COMPILE Consortium provides secure transfer and storage of from collaborating RCTs in compliance with the NYU security requirements for data collection, user access, file transfer, and security both in transit and in motion. The security requisites for data transfer for the COPILE project are compliant with the HIPPA & HITECH of USA, GDPR of EU, Canada's PIPEDA and the Australia's CDR.

NYU has set up event transfer rules so that any file a collaborating RCT uploads to their account will immediately transfer to a corresponding folder for the this RCT in the NYU network drive. Following transfer, all data files with are deleted from the MFT folder. The Consortium Investigators transfer data files from their RCT to NYU's MCIT Research DataCore by secure file transfer protocol (sFTP) using the GlobalScape managed file transfer (MFT) solution. A designated person from the collaborating RCT is provided by NYU a unique account requiring valid credentials (user ID and password) to access their corresponding folders in the MFT system to deposit encrypted data files.

Upon approval for data use by the COMPILE Publication committee, NYU will provide to the approved data requestor a snapshot or data dump of the common database, which will be created and stored within a virtual device infrastructure (VDI), or virtual machine, residing within NYU's data center. A data analyst/programmer designated by the approved data requestor will be provided with an NYU Kerberos ID and password in order to access the VDI remotely and perform all analyses within the VDI. All statistical applications, analyses datasets, and programming will remain within the VDI.

Submissions and access to the COMPILE database necessitates issuance to designated members of the collaborating RCT of unique identification credentials, a password, verification code, or other electronic signature codes. The investigators are obligated to keep, and cause their designees to keep, the ID/Passwords in confidence and use them solely for purposes of transferring data and accessing the COMPILE database.

Figure S.2.c. A schematic representation of COMPILE secure data transfer and access

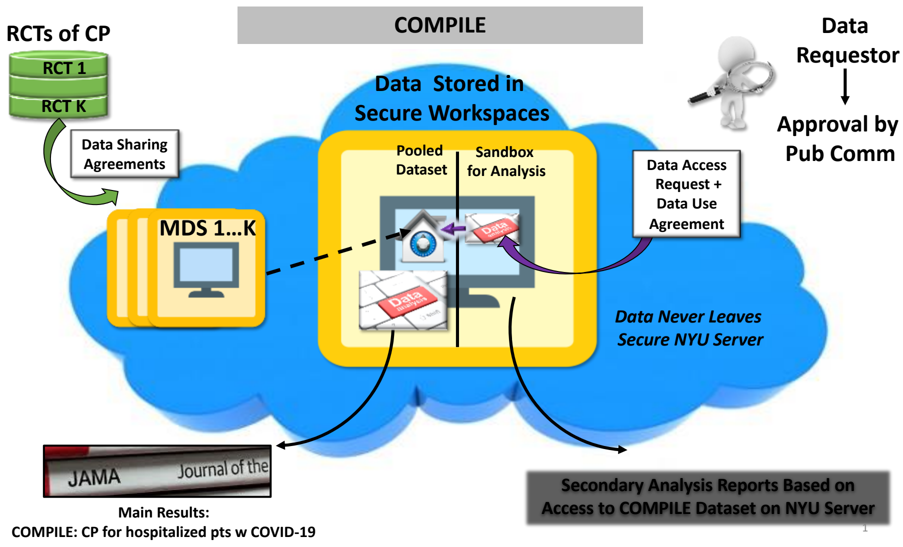

© 2022 Troxel AB et al. *JAMA Network Open.*

Minimal data set instructions

Data Delivery Instructions for COMPILE

1. Introduction to the Data Request Packet

This Data Request Packet (DRP) and accompanying contents are intended to provide information and instruction to assist with the assembly and delivery of COMPILE data to NYU. COMPILE data will be sent to NYU as two types. Randomized clinical trial (RCT) level data (i.e. randomization, blinding, control treatment) will be the first data transfer and only delivered once (single delivery). Cumulative subject-level data will then be delivered every two weeks, beginning August 1, 2020 (recurrent delivery).

Subjects must meet the following conditions to be included in the data:

1) **Subject was not on ventilator at time of randomization**
2) **If subject was randomized to receive convalescent plasma, the plasma was confirmed to be convalescent (meets local antibody level threshold).**

This document lists the DRP components and their intended uses and provides contact information when questions arise regarding the assembly of the data delivery files.

2. Content and use of DRP components

Section 2.1. **Output File Instructions** describe the format of the data delivery file, which will contain the data elements from the data dictionary.

Section 2.2. **Data Dictionaries** describe the data elements, data elements formats, and accompanying data element.

Section 2.3. **QC Components** recommend data quality checks to perform against data intended for delivery to the NYU Team prior to actual data delivery.

Section 2.4. **Transfer and Delivery Instructions** describe the method of delivery of the data files to the NYU Team.

Section 2.5 **COMPILE ID Assignment** describes the process through which COMPILE IDs are assigned.

Section 3. **Contact Information** provides contact information if there are questions regarding data delivery.

Please read through the entirety of this document. The final delivery file requirements are a combination of Section 2.1 which describes how to package the extracted data into individual site records and assemble them into a completed file ready for delivery to the COMPILE

© 2022 Troxel AB et al. *JAMA Network Open.*

Consortium AND Section 2.2 which describes how to map the requested data elements. Note that these sections will cross-reference each other.

2.1 Output file instructions

The data dictionary tables in section 2.2 provide descriptions of the data elements for each unique file (i.e. data point for each patient).

2.1.1 **File-level components:**

2.1.1.1 The data format type will be a flat file, also commonly known as a comma-delimited or comma-separated file (.csv).

2.1.1.2 Data element names, commonly known as variable names or column names, will be delivered in the first row of the data output file. Each data element variable listed within a given data dictionary table should be included in the first record (row).

2.1.2 **Naming instructions for the files:**

2.1.2.1 Each file should be constructed with the following naming convention of three or four components and the standard ".csv" filename extension.

File containing RCT Information:
COMPILE_*RCT ID*_RCT_INFO _*date***.***csv*

Files containing cumulative individual Subject data:
COMPILE_*RCT ID _date***.***csv*

2.1.2.2 The Consortium acronym will be included as the first component of the output file name (i.e. "COMPILE") for both RCT and subject-level data files.

2.1.2.3 After the Consortium acronym, include an underscore ("**_**"). **Do not** use a space (" ") in the file name.

2.1.2.4 The *"RCT ID"* should be included next. Each RCT will be assigned a COMPILE RCT #.

2.1.2.5 After the *"RCT ID"* include an underscore ("**_**"). **Do not** use a space (" ") in the file name.

2.1.2.6 For RCT level data only: Next include "RCT_INFO". After the "RCT_INFO" include an underscore ("_"). **Do not** use a space (" ") in the file name.

2.1.2.7 The date that the file was assembled will be included as the last component in the output filename. The formatted date should not include any separators such as hyphens or slashes. First,

© 2022 Troxel AB et al. *JAMA Network Open.*

include the 4-digit year (YYYY), then the 2-digit month (MM), and lastly the 2-digit day (DD) so that the output appears as YYYYMMDD. If the numeric value of the month or day is 1-9, then precede the values with a zero.

2.1.2.7 All filenames should include the standard ".csv" filename extension.

2.1.2.8 Example of RCT Level Data filename

Output File Name	RCT #	Fixed Value	Assembly Date	Output Filename
COMPILE	XX	RCT INFO	May 2, 2020	COMPILE_XX_RCT_INFO_20200502.csv

2.1.2.9 Example of Subject Level Data filename

Output File Name	RCT #	Assembly Date	Output Filename
COMPILE	XX	May 2, 2020	COMPILE_XX_20200502.csv

2.1.3 **Record-level components:**

2.1.3.1 An observation in any field that contains no data being delivered because it is either missing, unknown, or not-applicable should be represented with the missing value code of -999.

Every effort should be made to find and report missing data. Given that trials and data collection are ongoing, it is expected that data could be missing at the time of one upload, but later obtained and included in a following upload.

2.2. Data Dictionaries

These tables describe the analytic variables corresponding to the files to be delivered in the data submission for each COMPILE data file. COMPILE RCT INFO data will be delivered to NYU once. COMPILE subject-level data will be delivered every two weeks.

COMPILE RCT INFO

Table name: COMPILERCT
Version: 1
Record Structure: One record per RCT.
Missing Data: Enter -999 for any missing, unknown, or not-applicable data

Sequence Number	Variable Name to be included in data file	Data Element Text	Formatting and/or Permissible Values	Instructions and Notes
1	tr_blind	Blinding	0=No	
			1= Single blind	

© 2022 Troxel AB et al. *JAMA Network Open.*

			2= Double blind	
2	tr_rand	Randomization	0= No	
			1=Yes	
3	tr_units	Number of units of CP/control treatment	Integer ≥ 0	
4	tr_cntrl	Control treatment	0= Standard of Care	
			1= Non-convalescent Plasma	
			2= Saline/LR with coloring agent	
5	tr_rct_id	COMPILE RCT identifier	character (length=2)	RCTs will be assigned COMPILE RCT IDs
6	tr_sites	Number of RCT sites	Integer ≥ 0	
7	tr_date	Date the RCT opened enrollment	YYYYMMDD	

COMPILE

Table name: COMPILE
Version: 1
Record Structure: One record per subject.
Missing Data: Enter -999 for any missing, unknown, or not-applicable data

Sequence Number	Variable Name to be included in data file	Data Element Text	Formatting and/or Permissible Values	Instructions and Notes
**Patient Baseline Characteristics**				
1	tr_rct_id	COMPILE RCT identifier	character (length=2)	RCTs will be assigned COMPILE RCT IDs
2	site_id	COMPILE site within RCT ID	character (length=2)	Site within RCT will be assigned COMPILE RCT/site IDs
3	pt_id	COMPILE patient ID at RCT	character (length=3)	Patients will be given COMPILE IDs
4	bl_enrollqtr	Quarter during which patient was enrolled	1= Jan-March 2020	
			2= Apr- June 2020	
			3= July- Sept 2020	
			4= Oct-Dec 2020	
			5= Jan-March 2021	
			6= Apr-June 2021	
			7=July- Sept 2021	
			8= Oct-Dec 2021	
5	bl_treatment	Treatment assignment	0= Control	

© 2022 Troxel AB et al. *JAMA Network Open.*

			1= Convalescent Plasma	
6	bl_rx_trt_received	Randomized treatment received	0 = No	Select "Yes" if patient received the treatment she/he was randomized to receive. Select "No" otherwise.
			1 = Yes	
7	bl_age	Age in years	Integer ≥ 0, -999	
8	bl_sex	Sex	0= Male	
			1= Female	
			2=Other	
			Missing=-999	
9	bl_race[1]	Race	0= American Indian/Alaska Native	Report as "Mixed Race/Other" if multiple race selections
			1= Black or African American	
			2= White	
			3= Asian	
			4= Native Hawaiian or Pacific Islander	
			5=Mixed Race/Other	
			Missing=-999	
10	bl_hispanic[1]	Hispanic ethnicity	0= No	
			1= Yes	
			Missing=-999	
11	bl_blood	Blood group	0= O	
			1= A	
			2= B	
			3=AB	
12	bl_diabetes	History of diabetes (all types)	0= No	
			1= Yes	
			Missing=-999	
13	bl_pulm	History of pulmonary disease	0= No	
			1= Yes	
			Missing=-999	
14	bl_cardio	History of cardiovascular disease	0= No	
			1= Yes	
			Missing=-999	
15	bl_symp_days	Days since symptoms onset at time of randomization	1= 0 to 3	
			2= 4 to 6	
			3= 7 to 10	
			4= 11-14	

© 2022 Troxel AB et al. *JAMA Network Open.*

			5= more than 14<br>Missing=-999	
16	bl_dx_days	Days since COVID-19 diagnosis at time of randomization	Integer ≥ 0, -999	
17	bl_status[2]	Status at time of randomization	0= Outpatient<br>1= Hospitalized non ICU<br>2= Hospitalized, ICU level care, not on mechanical ventilation<br>3= Hospitalized, ICU level care, on mechanical ventilation	
18	bl_enrl_days	Days from randomization to first infusion	Integer ≥ 0, -999	Enter 0 if treatment is on the day of randomization or if patient is randomized to standard of care
**Concomitant Medications[3]**				
19	cm_hcqs	Hydroxychloroquine	0= No<br>1= Yes<br>Missing=-999	Select "Yes" if taken at time of randomization. Select "No" if not taken at time of randomization.
20	cm_abx	Antibacterial	0= No<br>1= Yes<br>Missing=-999	Select "Yes" if taken at time of randomization. Select "No" if not taken at time of randomization.
21	cm_antiviral	Antiviral<br>(not remdesivir)	0= No<br>1= Yes<br>Missing=-999	Select "Yes" if taken at time of randomization. Select "No" if not taken at time of randomization.
22	cm_rmdsvr	Remdesivir	0= No<br>1= Yes<br>Missing=-999	Select "Yes" if taken at time of randomization. Select "No" if not taken at time of randomization.
23	cm_antiinflam	Anti-inflammatory (non-steroids)	0= No<br>1= Yes<br>Missing=-999	Select "Yes" if taken at time of randomization. Select "No" if not taken at time of randomization.
24	cm_steroids	Steroids	0= No<br>1= Yes<br>Missing=-999	Select "Yes" if taken at time of randomization. Select "No" if not taken at time of randomization.
25	cm_thromb	Antithrombotic	0= No<br>1= Yes<br>Missing=-999	Select "Yes" if taken at any point during study. Select "No" if never taken during study.
26	cm_db_rmdsvr	Double blind remdesivir	0= No<br>1= Yes<br>Missing=-999	Select "Yes" if patient was also part of a double blind RCT of remdesivir at time of

© 2022 Troxel AB et al. *JAMA Network Open.*

				randomization. Select "No" otherwise
27	cm_db_hsqs	Double blind hydroxichloroquine	0= No<br>1= Yes<br>Missing=-999	Select "Yes" if patient was also part of a double blind RCT of hydroxychloroquineat time of randomization. Select "No" otherwise
28	cm_nonrct_cp	Convalescent plasma outside the RCT	0= No<br>1= Yes<br>Missing=-999	Select "Yes" if patient received convalescent plasma outside of the RCT at time of randomization. Select "No" otherwise
29	cm_db_other	Any double blind RCT other than hydroxychloroquine or remdesivir	0= No<br>1= Yes<br>Missing=-999	Select "Yes" if patient was also part of any double blind RCT other than for hydroxychloroquine or remdesivir at time of randomization. Select "No" otherwise
**Adverse Events**				
30	ae_trali	TRALI[4]	0= No<br>1= Yes	
31	ae_taco	TACO[5]	0= No<br>1= Yes	
32	ae_ttc	TACO/TRALI/worsening C OVID - overlap and undifferentiated reactions	0 = No<br>1 = Yes	
33	ae_reaction	Transfusion reaction (other than TRALI or TACO)[6]	0=No<br>1= Yes	
34	ae_arterial	Arterial thrombotic event (MI, stroke, acute limb ischemia) after randomization while in hospital	0=No<br>1= Yes	
35	ae_venous	Venous thrombotic event (pulmonary embolism, DVT) after randomization while in hospital	0= No<br>1= Yes	
**Outcomes**				
36	out_who0	WHO ordinal scale[7] at day 0	0-10, -999	Day 0=day of randomization
37	out_who1	WHO ordinal scale at day 1	0-10, -999	
38	out_who2	WHO ordinal scale at day 2	0-10, -999	
39	out_who3	WHO ordinal scale at day 3	0-10, -999	
40	out_who4	WHO ordinal scale at day 4	0-10, -999	
41	out_who5	WHO ordinal scale at day 5	0-10, -999	
42	out_who6	WHO ordinal scale at day 6	0-10, -999	

© 2022 Troxel AB et al. *JAMA Network Open.*

43	out_who7	WHO ordinal scale at day 7	0-10, -999	
44	out_who8	WHO ordinal scale at day 8	0-10, -999	
45	out_who9	WHO ordinal scale at day 9	0-10, -999	
46	out_who10	WHO ordinal scale at day 10	0-10, -999	
47	out_who11	WHO ordinal scale at day 11	0-10, -999	
48	out_who12	WHO ordinal scale at day 12	0-10, -999	
49	out_who13	WHO ordinal scale at day 13	0-10, -999	
50	out_who14	WHO ordinal scale at day 14	0-10, -999	
51	out_who15	WHO ordinal scale at day 15	0-10, -999	
52	out_who16	WHO ordinal scale at day 16	0-10, -999	
53	out_who17	WHO ordinal scale at day 17	0-10, -999	
54	out_who18	WHO ordinal scale at day 18	0-10, -999	
55	out_who19	WHO ordinal scale at day 19	0-10, -999	
56	out_who20	WHO ordinal scale at day 20	0-10, -999	
57	out_who21	WHO ordinal scale at day 21	0-10, -999	
58	out_who22	WHO ordinal scale at day 22	0-10, -999	
59	out_who23	WHO ordinal scale at day 23	0-10, -999	
60	out_who24	WHO ordinal scale at day 24	0-10, -999	
61	out_who25	WHO ordinal scale at day 25	0-10, -999	
62	out_who26	WHO ordinal scale at day 26	0-10, -999	
63	out_who27	WHO ordinal scale at day 27	0-10, -999	
64	out_who28	WHO ordinal scale at day 28	0-10, -999	
65	out_who29	WHO ordinal scale at day 29	0-10, -999	
66	out_who30	WHO ordinal scale at day 30	0-10, -999	
67	out_who31	WHO ordinal scale at day 31	0-10, -999	
68	out_who32	WHO ordinal scale at day 32	0-10, -999	
69	out_who33[8]	WHO ordinal scale at day 33	0-10, -999	
70	out_death14	Whether deceased by day 14 ± 1	0 = No;<br>1 = Yes;<br>-1= patient has not yet reached day 14±1;<br>-999 = missing	
71	out_death	Whether deceased by day 28 ± 2	0 = No;<br>1 = Yes;<br>-1 = patient have not yet reached day 28±2;<br>-999 = missing	If deceased at day 14 ± 1, enter "Yes" deceased at day 28 ± 2
72	out_deathday	Days to death since day of randomization	Integer ≥ 0, -999	Number of days from day 0 (day of randomization) to day of death
73	out_discharge	Days to discharge since day of randomization	-1 = subject is still in the hospital;<br>-2 = patient died in the hospital;<br>Integer ≥ 0 number of days from randomization to discharge;	Number of days from day 0 (day of randomization) to day of discharge

© 2022 Troxel AB et al. *JAMA Network Open.*

			-999 = days from randomization to discharge is missing	
Characteristics of the antibodies of the CP[9]				
74	Characteristic A[10]	Quantity of antibodies	# in units specific to the measurement platform, -999	Leave blank for patients randomized to control
75	Characteristic B[10]	Quantity of antibodies	# in units specific to the measurement platform, -999	Leave blank for patients randomized to control
76	Characteristic C[11]	Quantity of antibodies	# in units specific to the measurement platform, -999	Leave blank for patients randomized to control

[1]Only for RCTs in the USA
[2]Only hospitalized patients that are not on mechanical ventilation at time of randomization are eligible for the COMPILE study.
[3]Consult the document "COMPILE ConMed dictionary" on COMPILE website for categorization of medications
[4]TRALI (Transfusion related acute lung injury): Less common: (i) Acute hypoxemia and non-cardiogeneic pulmonary edema within 6 hours of transfusion; (ii) Often associated with hypotension and fever; (iii) Treatment is supportive care without diuresis
[5]TACO (Transfusion associated circulatory overload): More Common: (i) Acute hypoxemia, respiratory distress, and pulmonary edema within 6 hours of transfusion; (ii) Often associated with hypertension; (iii) Signs of volume overload needing diuresis as treatment
[6]Other than TRALI or TACO: (i) Mild: hives, itching, flushing, fever; (ii) Severe: anaphylaxis including bronchospasm, angioedema, hypotension.
[7]WHO 11-points scale. See the Statistical analysis plan for how WHO 7- and 8-points scales should be converted to the WHO 11-point scale < doi:10.1016/S1473-3099(20)30483-7>
[8]Include more columns if your RCT collects WHO status longer than 33 days post-randomization
[9]Only for patients who were randomized to convalescent plasma (CP)
[10]Provide description of each of the characteristics
[11]Add more columns if more than 3 characteristics are measured on the CP

2.3 QC Components

The NYU Team would like to perform, at a minimum, the following quality controls on the data delivery. The primary purpose of the quality control will be to check for data format errors and erroneous data. Please view the following QC components that will be performed at data upload.

2.3.1 Data validation performed

The following data validation checks will be performed after receiving of data upload. If a file import fails any of the following checks it will be requested for resolution. The file must be corrected before it may attempt to upload again. Allowable timeframe for resolution and re-upload is 48 hours. For assistance, please see section 3.0 for contact information.

2.3.1.1 Duplicate Records

Confirm that the data file does not contain duplicate records.

© 2022 Troxel AB et al. *JAMA Network Open.*

2.3.1.2 Null Records

Confirm that there are no null values in any of the variables. There should be a value for each data observation (i.e., for each record and each variable). Please use the missing (-999) code to fill in any null values in the data set.

2.3.1.3 Data Value Type Matching

Confirm that all values in any variable must match the specified value type. This means that a text value may not be entered into a date field.

2.3.1.3 Look Up List Matching

Confirm that all values in fields with specified value options match. An example of this is that values of 'TR_BLIND" are "0", "1", or "2".

2.3.1.4 Import Template Validation

Confirm that an imported file matches the designated import file template, and that all variable names are correct and there are no missing variables in the data import. Individual observations for a variable may be coded as missing (-999), but entire variables/columns may not be omitted from the data set. If a variable is not collected in a RCT, include the variable in the data set and enter -999 for every observation.

2.3.1.5 Permissible value range validation

For any variable that contains permissible values confirm valid permissible value(s) for that field as defined in the data dictionary. No field should be left blank or unaccounted for in the data delivery. Data elements should not contain unexpected values not listed as permissible values in the data dictionary.

2.3.1.6 Missing Data

Please refer to section 2.1.3 (Record-level components) for specific instructions regarding handling of missing data.

2.4 Transfer and Delivery of Data Instructions

All data should be submitted electronically using the NYU secured FTP system. Email data submissions will **not** be permitted.

2.5 COMPILE ID Assignment

© 2022 Troxel AB et al. *JAMA Network Open.*

Each RCT study participant will be assigned a COMPILE ID. Please review the following steps that describe the process through which COMPILE IDs are assigned and data is prepared.

1. NYU will assign each RCT a COMPILE RCT ID.
2. RCT includes the given COMPILE RCT ID in “RCT Info” file and submits data to NYU.
3. NYU will supply each RCT with a COMPILE ID Log Template based on trial data available in “RCT Info” file.
4. RCT will use log template to assign each study participant their COMPILE ID using the three identification components: COMPILE RCT ID, COMPILE site ID, and COMPILE patient ID. Site is considered any hospital (or a set of hospitals) with a separate randomization sequence. RCTs will assign COMPILE site ID to their recruitment sites as 01, 02, etc.
5. RCT will include COMPILE ID fields in cumulative, subject-level data that is submitted every two weeks.

2.5.1 COMPILE ID Log Template

An example of a log with COMPILE patient IDs is presented below. The example reflects a collaborating RCT that has two recruitment sites. Each RCT will receive a log similar to this. This log will be kept locally at each RCT location and never shared with the COMPILE Consortium to ensure de-identification of the data.

The shaded columns will be required in the minimal data set. More COMPILE patient IDs will be added in obvious fashion if a recruitment site has more than 100 patients randomized.

RCT Name	RCT Recruitment site	RCT Subject ID #	COMPILE RCT Identifier	COMPILE RCT recruitment site identifier	COMPILE patient ID
			XX	01	001
			XX	01	002
			XX	01	003
			XX	01	…….
			XX	01	100
			XX	02	001
			XX	02	…..
			XX	02	100

3.0 Questions and contact information

If you have any questions regarding this data request, please contact:

Eva Petkova
Eva.petkova@nyulangone.org
T: 917-519-0245

© 2022 Troxel AB et al. *JAMA Network Open.*

Katherine Armstrong
katherine.armstrong@nyulangone.org
T: 212-404-3456

© 2022 Troxel AB et al. *JAMA Network Open.*

eAppendix 4. RCT-Specific Information

AA: CONTAIN COVID-19

Trial name: CONTAIN-COVID-19

Principal Investigator(s): Mila B. Ortigoza, MD PhD, Liise-anne Pirofski, MD

Number of sites: 21

Location(s): United States (Connecticut, Florida, New York, Texas, Wisconsin)

Number of units in CCP group: 1

Control arm: Saline

Blinding: Double

Randomization: 1:1, variable permuted blocks, stratified by site and risk of severe disease†

Eligibility:
Inclusion

- Hospitalized ≤ 72 or within seven days of first symptoms
- One or more of
  - Cough
  - Chest pain
  - Shortness of breath
  - Fever
  - Oxygen saturation ≤ 94%
  - Abnormal CXR/CT imaging

Exclusion

- Receipt of any Covid-19 vaccine or participation in a Covid-19 vaccine study as a subject.
- Receipt of pooled immunoglobulin within 30 days
- Contraindication or prior reaction to transfusion blood products
- Volume overload secondary to congestive heart failure or renal failure
- Unlikely to survive past 72 hours based on Investigator judgment

Antibody detection: Prospective, New York Blood Center platforms assessing SARS CoV-2 IgG

Primary outcome: Status on the WHO 11-point scale at 14 days

Total enrolled: 941, April 17, 2020 to March 15, 2021

Total qualified for pooling: 940 (100%)

© 2022 Troxel AB et al. *JAMA Network Open.*

†higher risk if aged ≥ 60 years, or < 60 years and with at least one of the following: chronic pulmonary condition (chronic obstructive pulmonary disease, obstructive sleep apnea, interstitial lung disease); chronic heart condition (congestive heart failure with New York Heart Association score ≥ 2, atrial fibrillation, ischemic heart disease); hypertension; chronic kidney disease with eGFR < 60 mL/min; body mass index ≥ 35; diabetes mellitus; or immunosuppression (CD4 count < 200, on immunosuppressive medications for autoimmune conditions, cancers, solid or stem cell transplants, steroids such as prednisone > 10 mg/day or equivalent)
* on vent at rand, no confirmed COVID-19, patient declined to consent, symptom duration > 7 days, not requiring oxygen support, impending respiratory failure, received Covid-19 vaccine, volume overload.
Table S.1.2 below provides the full list of personnel for the CONTAIN-COVID-19 study.

Full list of personnel for CONTAIN COVID-19

Name	Role	Site
**CONTAIN Coordinating Center**		
Judith S. Hochman, MD	Project PI	NYU Langone Health
Corita R. Grudzen, MD, MSHS	Project PI	NYU Langone Health
Deborah Keeling, MS	Project Finance	NYU Langone Health
Norka Rappoport	Project Finance	NYU Langone Health
Sam F. Ebel, JD, JD, MPH	Project Contracts	NYU Langone Health
Jayne Y. Kim, PhD	Project Regulatory Coordinator	NYU Langone Health
Mila B. Ortigoza, MD, PhD	Study PI, IND Holder	NYU Langone Health
Liise-anne Pirofski, MD	Study PI	Albert Einstein College of Medicine
Hyun Ah Yoon, MD	Study Co-PI	Albert Einstein College of Medicine
Gia F. Cobb, MA	Lead Study Project Manager (current)	NYU Langone Health
Michelle Chang, MS, MPH	Study Project Manager (previous)	NYU Langone Health
Kevin Chan	Study Project Manager (previous)	NYU Langone Health
Fatema Z. Rahman, BS	Study Coordinator	NYU Langone Health
Payal Patel	Lead Programmer	NYU Langone Health
Anne Martocci	Programmer	NYU Langone Health
Shivang Dave, MS	Programmer	NYU Langone Health
Yousef Darwish	Programmer	NYU Langone Health
Monica Taveras, BS	Study Data Manager (current)	NYU Langone Health
Victoria Shoyelu	Study Data Manager (previous)	NYU Langone Health
Andrea A. Asencio, BA, CCRC	Study Data Manager (unblinded)	Albert Einstein College of Medicine
Alexander Bragat, MBA	Study Associate Data Manager	NYU Langone Health

© 2022 Troxel AB et al. *JAMA Network Open.*

Patrick Xin	Study Associate Data Manager	NYU Langone Health
Jeff LaFleur, MA	Biorepository Manager	Albert Einstein College of Medicine
Johanna Rivera, PhD	Study Research Scientist	Albert Einstein College of Medicine
Jonathan R Lai, PhD	Study Research Scientist	Albert Einstein College of Medicine
Kartik Chandran, PhD	Study Research Scientist	Albert Einstein College of Medicine
Gorka Lasso, PhD	Study Research Scientist	Albert Einstein College of Medicine
Levi Cleare, BA	Study Research Scientist	Albert Einstein College of Medicine
Olivia Vergnolle, PhD	Study Research Scientist	Albert Einstein College of Medicine
Chowdhury Raihan Bikash, PhD	Study Research Scientist	Albert Einstein College of Medicine
Antonio Nakouzi, BS	Study Research Scientist	Albert Einstein College of Medicine
Gregory Quevedo, BS	Study Research Scientist	Albert Einstein College of Medicine
Robert H. Bortz, III	Study Research scientist	Albert Einstein College of Medicine
Ariel S. Wirchnianski, MS	Study Research scientist	Albert Einstein College of Medicine
Catalina Florez, PhD	Study Research scientist	Albert Einstein College of Medicine
Rachelle Babb, PhD	Study Research Scientist	Albert Einstein College of Medicine
Keith S. Goldfeld, DrPH, MS, MPA	Lead Study Statistician (blinded)	NYU Langone Health
Andrea B. Troxel, ScD	Study Statistician (blinded)	NYU Langone Health
Eva Petkova, PhD	Lead Study Statistician (unblinded)	NYU Langone Health
Mengling Liu, PhD	Study Statistician (unblinded)	NYU Langone Health
Yinxiang Wu, MA	Study Statistician (unblinded)	NYU Langone Health
Danni Wu, MS	Study Statistician (unblinded)	NYU Langone Health
Yi Li, MS	Study Statistician (unblinded)	NYU Langone Health

**NYU Langone Health Consortium**		
Mila B. Ortigoza, MD, PhD	Site PI	NYU Langone Health (all locations)

© 2022 Troxel AB et al. *JAMA Network Open.*

Gia F. Cobb, MA	Site Project Manager	NYU Langone Health
Fatema Z. Rahman, BS	Associate Site Coordinator	NYU Langone Health
Adeyinka O. Ajayi, MD, MPH	Study Coordinator	NYU Langone Health
Sara L. Rodriguez, MBA	Regulatory Coordinator	NYU Langone Health
Ana G. Ledesma, BA	Data Coordinator	NYU Langone Health
Eduardo Iturrate, MD, MSW	Site Associate Data Manager	NYU Langone Health
Gillian Baptiste, MD	Lead Sub-Investigator	NYU Langone Health - Manhattan
Ioannis Zacharioudakis, MD	Lead Sub-Investigator	NYU Langone Health - Brooklyn
Mary O'Keefee, MD	Lead Co-Investigator	NYU Langone Health – Long Island
Rabi Upadhyay, MD, PhD	Site PI	Bellevue Hospital
Tania Kupferman, MD	Lead Sub-Investigator	Bellevue Hospital
Jeffrey G. Schneider, MD	Lead Site Coordinator	NYU Langone Health – Long Island
Lee C. Moldolsky, MSN, RN	Transfusion Liaison	NYU Langone Health – Long Island
Brian J. Raimondo, BSN	Transfusion Liaison	NYU Langone Health - Manhattan
Sarah Mendez	Transfusion Liaison	NYU Langone Health - Manhattan
Patricia Hughes	Transfusion Liaison	NYU Langone Health - Manhattan
Lalitha Parameswaran, MD, MPH	Sub-Investigator	NYU Langone Health - Brooklyn
Stephanie Sterling, MD	Sub-Investigator	NYU Langone Health - Brooklyn
Aaron S. Lord, MD, MSc	Sub-Investigator	NYU Langone Health - Brooklyn
Shadi Yaghi, MD	Sub-Investigator	NYU Langone Health - Brooklyn
Anthony T. Corcoran, MD	Sub-Investigator	NYU Langone Health – Long Island
Abhinav Rohatgi, MD	Sub-Investigator	NYU Langone Health – Long Island
Marta W. Wronska, MO	Sub-Investigator	NYU Langone Health – Long Island
Karen G. Veloso, MD	Sub-Investigator	NYU Langone Health – Long Island
Masooma Sheikh, MD	Sub-Investigator	NYU Langone Health – Long Island
Erica Visconti-Ferrara, DO	Sub-Investigator	NYU Langone Health – Long Island
Xinyuan Wu, MD	Sub-Investigator	NYU Langone Health
Ranjini Srinivasan, MD	Sub-Investigator	NYU Langone Health
Fang-Ming Deng, MD, PhD	Sub-Investigator	NYU Langone Health
Thomas D. Filardo, MD	Sub-Investigator	NYU Langone Health
Jay Pendse, MD, PhD	Sub-Investigator	NYU Langone Health
Andrew Fleming, MD	Sub-Investigator	NYU Langone Health
Simone Blaser, MD	Sub-Investigator	NYU Langone Health
Olga Whyte, RN, BSN	Research Nurse Manager	NYU Langone Health, Bellevue

© 2022 Troxel AB et al. *JAMA Network Open.*

Jacqueline M. Gallagher, MSN, RN	Research Nurse	NYU Langone Health, Bellevue
Ololade E. Thomas, BSN, RN, MPH	Research Nurse	NYU Langone Health, Bellevue
Danibel Ramos, BSN, RN	Research Nurse	NYU Langone Health, Bellevue
Caroline Sturm-Reganato, RN	Research Nurse	NYU Langone Health, Bellevue
Charlotte C. Fong, RN, BS	Research Nurse	NYU Langone Health, Bellevue
Ivy M. Daus, RN, BSN	Research Nurse	NYU Langone Health, Bellevue
Arianne Gisselle Payoen, RN	Research Nurse	NYU Langone Health, Bellevue
Heekoung Youn, RN	Research Nurse	NYU Langone Health, Bellevue
Baby Jane Fran, RN	Research Nurse	NYU Langone Health, Bellevue
Rosario Medina, RN, MA	Research Nurse	NYU Langone Health, Bellevue
Renee McKell	Site Coordinator	NYU Langone Health – Brooklyn
Saila Khan	Site Coordinator	NYU Langone Health – Long Island
Timothy Hilbert, MD, PhD, JD	Blood Bank, Lead Rand Key Holder	NYU Langone Health - Manhattan
Tanya Hamilton	Blood Bank, Rand Key Holder	NYU Langone Health - Manhattan
Carlos J. Sanchez, BS, MT	Blood Bank, Rand Key Holder	NYU Langone Health - Brooklyn
Ding Wen Wu, MD, PhD	Blood Bank, Rand Key Holder	NYU Langone Health - Brooklyn
Joseph T. Chiofolo, DO, MS	Blood Bank, Rand Key Holder	NYU Langone Health – Long Island
Mark T. Friedman, DO	Blood Bank, Rand Key Holder	NYU Langone Health – Long Island
Nandini H. Patel, MS, MLS(ASCP)	Blood Bank	NYU Langone Health – Long Island
Jessica L. Jacobson, MD	Blood Bank	Bellevue Hospital
**Albert Einstein College of Medicine / Montefiore Medical Center**		
Marla Keller, MD	Project PI	Albert Einstein College of Medicine
Liise-anne Pirofski, MD	Site PI	Albert Einstein College of Medicine
Hyun ah Yoon, MD	Site Co-PI	Albert Einstein College of Medicine
Andrea Asencio, BA	Site Coordinator, Rand Key Holder	Albert Einstein College of Medicine
Jennifer Ayala, BA	Regulatory coordinator	Albert Einstein College of Medicine
K. Zoe Tsagaris, MS	Regulatory coordinator	Albert Einstein College of Medicine
Andria James, BS	Regulatory coordinator	Albert Einstein College of Medicine

© 2022 Troxel AB et al. *JAMA Network Open.*

Isaiah Eke, MD MPH	Site Coordinator	Albert Einstein College of Medicine
Aisha Obeidallah, BA	Site Coordinator	Albert Einstein College of Medicine
Oana A Sandu, MD	Data Coordinator	Albert Einstein College of Medicine
Sophie Sohval, MD	Data Coordinator	Albert Einstein College of Medicine
Monika Paroder, MD PhD	Blood bank, Rand Key Holder	Albert Einstein College of Medicine
Leana Serrano-Rahman, MPH	Blood bank	Albert Einstein College of Medicine
Joan Uehlinger, MD	Blood bank	Albert Einstein College of Medicine
Uzma Sarwar, MD	Sub-Investigator	Albert Einstein College of Medicine
Marilou Corpuz, MD	Sub-Investigator	Albert Einstein College of Medicine
Laura Cheney, MD PhD	Sub-Investigator	Albert Einstein College of Medicine
Johanna Daily, MD MS	Sub-Investigator	Albert Einstein College of Medicine
Rachel Bartash, MD	Sub-Investigator	Albert Einstein College of Medicine
Aya Al-Abduladheem, MD	Sub-Investigator	Albert Einstein College of Medicine
Inessa Gendlina, MD PhD	Sub-Investigator	Albert Einstein College of Medicine
Carol Sheridan, RN, MSN	Research nurse	Albert Einstein College of Medicine
Anna Bortnick, MD PhD MS	Sub-Investigator	Albert Einstein College of Medicine
Jeremy Eichler, BS	Site Coordinator	Albert Einstein College of Medicine
Rachel Kaufman, BS	Site Coordinator	Albert Einstein College of Medicine
Sarah Yukelis, BA	Site Coordinator	Albert Einstein College of Medicine
Michael Pennock, MD	Site Coordinator	Albert Einstein College of Medicine
Michelle Goggin, PhD	Site Coordinator	Albert Einstein College of Medicine
Christine Shen, BS	Data Coordinator	Albert Einstein College of Medicine
Jayabhargav Annam, BS	Data Coordinator	Albert Einstein College of Medicine

© 2022 Troxel AB et al. *JAMA Network Open.*

Ahmed Khokhar, MD	Data Coordinator	Albert Einstein College of Medicine
Daniel Barboto, MD	Data Coordinator	Albert Einstein College of Medicine
Brianna J. Lally, MD	Data Coordinator	Albert Einstein College of Medicine
Audrey Lee, MS	Data Coordinator	Albert Einstein College of Medicine
Max Lee, MD	Data Coordinator	Albert Einstein College of Medicine
Xiuyi A. Yang, MS	Data Coordinator	Albert Einstein College of Medicine
Stephanie Allen, MD	Data Coordinator	Albert Einstein College of Medicine
Avinash Malaviya, MS	Data Coordinator	Albert Einstein College of Medicine
Omar Moussa, MD	Data Coordinator	Albert Einstein College of Medicine
Rosa Park, MS	Data Coordinator	Albert Einstein College of Medicine
Reise Sample, BFA	Data Coordinator	Albert Einstein College of Medicine
Andrea Bae, MS	Data Coordinator	Albert Einstein College of Medicine
Galit Benoni, MD	Data Coordinator	Albert Einstein College of Medicine
**Yale University Medical Center**		
Mahalia Desruisseaux, MD	Site PI	Yale University Medical Center
Alessandro Satin, MD	Site Co-PI	Yale University Medical Center
Lindsie L. Boerger, BA	Lead Site Coordinator	Yale University Medical Center
Lisa D. Baker, RN, BSN, OCN	Lead Site Coordinator	Yale University Medical Center
Martha A. Luther, BSN, MPH	Site Coordinator	Yale University Medical Center
Jeanne E. Hendrickson, MD	Blood Bank, Rand Key Holder	Yale University Medical Center
Christopher A. Tormey, MD	Blood Bank, Rand Key Holder	Yale University Medical Center
Lirim S. Ameti, MD	Sub-Investigator	Yale University Medical Center
Neima Briggs, MD, PhD	Sub-Investigator	Yale University Medical Center
Marjorie R. Golden, MD	Sub-Investigator	Yale University Medical Center
Michael Gormally, MD, PhD	Sub-Investigator	Yale University Medical Center
Gloria S. Huang, MD	Sub-Investigator	Yale University Medical Center
Raymond M. Johnson, MD, PhD	Sub-Investigator	Yale University Medical Center

© 2022 Troxel AB et al. *JAMA Network Open.*

Maudry Laurent-Rolle, MD, PhD, BS	Sub-Investigator	Yale University Medical Center
Alyssa R. Morrison, BS	Sub-Investigator	Yale University Medical Center
Sheela V. Shenoi, MD, MPH	Sub-Investigator	Yale University Medical Center
Michele Montagna-Hill	Site Data manager	Yale University Medical Center
Brooke N. Rivera, MSW	Site Data manager	Yale University Medical Center
Grace M. Cortezzo	Site Data manager	Yale University Medical Center
Oscar Bate Akide Ndunge	Sub-Investigator	Yale University Medical Center
Kay B. Debski, BS	Regulatory Coordinator	Yale University Medical Center
Amy Nicoletti	Regulatory Coordinator	Yale University Medical Center
Kerry De Benedictis	Research Assistant	Yale University Medical Center
**Johns Hopkins Medical Center**		
Christian A. Merlo, MD MPH	Site PI	Johns Hopkins Medical Center
David N. Hager, MD PhD	Sub-Investigator	Johns Hopkins Medical Center
Shivani Patel	Site Coordinator	Johns Hopkins Medical Center
Rivcah Davis	Blood Bank, Rand Key Holder	Johns Hopkins Medical Center
Christi Marshall	Blood Bank, Rand Key Holder	Johns Hopkins Medical Center
**University of Miami Consortium**		
Ralph L. Sacco, MD, MS	Project PI	UHealth Tower
Dushyantha T. Jayaweera, MD	Site PI	UHealth Tower, Jackson Memorial
Shweta Anjan, MD, MBBS	Site PI	UHealth Tower, Jackson Memorial
Yan Yun Wu, MD, PhD	Lead Sub-Investigator, Blood Bank, Rand Key Holder	UHealth Tower, Jackson Memorial
Miriam Andrea Duque Cuartas, MD	Blood Bank, Rand Key Holder	UHealth Tower, Jackson Memorial
Laura Beauchamps, MD	Sub-Investigator	Jackson Memorial
Jovanna Bertran-Lopez, MD, MPH	Sub-Investigator	Jackson Memorial
Jose Gonzales Zamora, MD	Sub-Investigator	Jackson Memorial
Maria Delgado-Lelievre, MD	Sub-Investigator	UHealth Tower
Sheela Dominguez, MBA, MA	Site Project Manager	UHealth Tower, Jackson Memorial
Chin Chin Lee, MSPH, MS, BS	Site Project Manager and Regulatory Coordinator	UHealth Tower, Jackson Memorial
Daru Lane Ransford, BA	Site Project Manager	UHealth Tower, Jackson Memorial
Halina Kusack, RN, BBM, MSN	Research Nurse Manager	UHealth Tower, Jackson Memorial
Vela Karakeshishyan, MD, BSN	Site Coordinator	UHealth Tower, Jackson Memorial
Americo Hajaz, MD	Site Coordinator	UHealth Tower, Jackson Memorial
Dasmany Deniz, BS	Site Coordinator	UHealth Tower, Jackson Memorial

© 2022 Troxel AB et al. *JAMA Network Open.*

Giovanni Garcia, BHSA	Site Coordinator	UHealth Tower, Jackson Memorial
Katheryn Dae, MD, BS	Site Coordinator	UHealth Tower
Patricia Blenet, MSN, BA	Site Coordinator	UHealth Tower
Deborah Jaffe, MS, BS	Site Coordinator	UHealth Tower
Lourdes Olson	Site Coordinator	UHealth Tower
Diane Sabogal, MSN, BSN	Site Coordinator	UHealth Tower
Olivia Blust, BA	Site Coordinator	UHealth Tower
Veronica Del Prete Perez, MD	Site Coordinator	Jackson Memorial
Claudia Bornia, MD	Site Coordinator	Jackson Memorial
Vanessa Rodriguez-Perez, BA	Data Coordinator	UHealth Tower, Jackson Memorial
Vivian Calderon, BS	Data Coordinator	UHealth Tower, Jackson Memorial
Rajan Ramdev, BA	Data Coordinator	UHealth Tower, Jackson Memorial
Aaliyah Jolly, BS	Data Coordinator	UHealth Tower, Jackson Memorial
Ivonne Guzman, BA	Data Coordinator	UHealth Tower, Jackson Memorial
Richard Guerra, BS	Data Coordinator	UHealth Tower, Jackson Memorial
Sebastian Brito, BS	Data Coordinator	UHealth Tower, Jackson Memorial
**Greater Texas COVID-19 Consortium (GTCC)**		
David D McPherson, MD	Project PI	UTHealth-Houston
Luis Ostrosky-Zeichner, MD	Site PI	UTHealth-Houston
Bela Patel, MD	Site Co-PI	UTHealth-Houston
Masayuki Nigo, MD	Site Co-PI	UTHealth-Houston
Reeba Mathew, MD	Site Co-PI	UTHealth-Houston
Amee Amin, MD	Site Co-PI	UTHealth-Houston
Henry E Wang, MD	Site Co-PI	UTHealth-Houston
Ryan M Huebinger, MD	Site Co-PI	UTHealth-Houston
Goutham Dronavalli, MD	Site Co-PI	UTHealth-Houston
Yu Bai, MD	Blood Bank	UTHealth-Houston
Carolyn Z Grimes, DrPH	Associate Site Coordinator	UTHealth-Houston
Karen W Eldin, MD	Blood Bank	UTHealth-Houston
Rhonda Hobbs	Blood Bank	UTHealth-Houston
Rebecca Denham, MPH	Blood Bank	UTHealth-Houston
John Dick II, MT, ASCP	Blood Bank	UTHealth-Houston
Virginia E Umana, BS	Site Project Manager	UTHealth-Houston
Maria D Hernandez, MS	Associate Site Coordinator	UTHealth-Houston
Laura E Nielsen, BSN, RN, CCRN	Research Nurse	UTHealth-Houston
Sami M Anjum, MS	Site Coordinator	UTHealth-Houston
Shelby C Mader, BS	Site Coordinator	UTHealth-Houston
Taylor P Stutz, BS	Site Coordinator	UTHealth-Houston
Mehriban Mammadova, MPH	Site Coordinator	UTHealth-Houston
Pamela Nichols, MPH	Site Coordinator	UTHealth-Houston

© 2022 Troxel AB et al. *JAMA Network Open.*

Tanya S Khan	Site Coordinator	UTHealth-Houston
Maha R Boktour, MPH	Site Coordinator	UTHealth-Houston
Andrew N Dentino, MD	Site PI	UT-RGV
Jessica G. Martin, MD	Site Co-PI	UT-RGV
Timothy R Heath, MD	Site Co-PI	UT-RGV
Fatimah O Bello, MD	Site Co-PI	UT-RGV
Brenda L Castaneda, RMA	Site Coordinator	UT-RGV
Brenda D Benitez	Site Coordinator	UT-RGV
Erik Hinojosa, BA	Site Coordinator	UT-RGV
Brenda C Guerra, MLS	Blood Bank	UT-RGV
Armando Ortiz, MT	Blood Bank	UT-RGV
Julie V Philley, MD	Site PI	UT-Tyler
Megan S Devine, MD	Site Co-PI	UT-Tyler
Renee S Hebbeler-Clark, MD	Sub-Investigator	UT-Tyler
Pamela J McShane, MD	Sub-Investigator	UT-Tyler
Rebekah Hibbard, BS, CCRC	Site Project Manager	UT-Tyler
Benji E Hawkins, CCRC	Site Coordinator	UT-Tyler
Elizabeth R Dohanich, BBA, CCRC	Site Coordinator	UT-Tyler
Carly Wadle, MPH	Site Coordinator	UT-Tyler
Kimberly L Greenlee, MPH	Data Coordinator	UT-Tyler
Jennifer Brooks, RN	Site Coordinator	UT-Tyler
Christopher D Herrick, BS, CCRC	Regulatory Coordinator	UT-Tyler
**Medical College of WI & Froedtert Hospital**		
Reza Shaker MD	Project PI	Medical College of WI & Froedtert Hospital
Rahul Nanchal MD, MS	Site PI	Medical College of WI & Froedtert Hospital
Amit Gode MBBS, MPH	Site Co-PI	Medical College of WI & Froedtert Hospital
Paul Bergl MD	Site Co-PI	Medical College of WI & Froedtert Hospital
Kurt Hu MD	Site Co-PI	Medical College of WI & Froedtert Hospital
Jayshil Patel MD	Site Co-PI	Medical College of WI & Froedtert Hospital
Bipin Thapa, MD, MS	Site Co-PI	Medical College of WI & Froedtert Hospital
Shankar Srinivasan	Site Project Manager	Medical College of WI & Froedtert Hospital

© 2022 Troxel AB et al. *JAMA Network Open.*

Jeanette Graf	Site Project Manager	Medical College of WI & Froedtert Hospital
Jennifer L. Peterson, PhD	Site Project Man., Rand Key Holder	Medical College of WI & Froedtert Hospital
Char Klis	Site Project Manager	Medical College of WI & Froedtert Hospital
Kelly Knauf	Lead Site Coordinator	Medical College of WI & Froedtert Hospital
Kyersten Reimer	Rand Key Holder	Medical College of WI & Froedtert Hospital
Erica Carpenter	Blood bank	Medical College of WI & Froedtert Hospital
Christine Naczek	Blood bank	Medical College of WI & Froedtert Hospital
Rae Petersen	Associate Site Coordinator	Medical College of WI & Froedtert Hospital
Renee Dex	Research Nurse Manager	Medical College of WI & Froedtert Hospital
Jennifer Drossart	Associate Site Coordinator	Medical College of WI & Froedtert Hospital
James Zelten	Associate Site Coordinator	Medical College of WI & Froedtert Hospital
**St. Luke’s Advocate Aurora Health**		
Charles Brummitt	Site PI	St. Luke’s Advocate Aurora Health
Mengyao Liang	Lead Sub-Investigator	St. Luke’s Advocate Aurora Health
Lynda Yanny	Research Nurse Manager	St. Luke’s Advocate Aurora Health
Gary Dennison	Regulatory Coordinator	St. Luke’s Advocate Aurora Health
Phyllis Runningen	Lead Site Coordinator	St. Luke’s Advocate Aurora Health
Brian Brzezinski	Rand Key Holder	St. Luke’s Advocate Aurora Health
Stephen Fiebig	Rand Key Holder	St. Luke’s Advocate Aurora Health
Erica Carpenter	Blood Bank	St. Luke’s Advocate Aurora Health
Chris Naczek	Blood Bank	St. Luke’s Advocate Aurora Health
Michelle Kasdorf	Associate Site Coordinator	St. Luke’s Advocate Aurora Health

Definition of Roles:

Role	Description
Project PI	Overarching PI named in grants
Project Finance	Oversees CONTAIN grants and distribution of funding to collaborating sites
Project Contracts	Oversees CONTAIN agreements and contracts with collaborating sites
Project Regulatory Coordinator	Oversees regulatory compliance at NYU site and CONTAIN liaison with FDA

© 2022 Troxel AB et al. *JAMA Network Open.*

Study PI	PI overseeing CONTAIN study at all sites
Site PI	PI lead for each site
Co-PI	Associate/Assistant PI for site or study
IND Holder	Named person in FDA’s IND application
Study Statistician	Study statistician. Can be designated as “Lead”, “Blinded”, or “Unblinded”.
Study Project Manager	PM overseeing CONTAIN study at all sites
Site Project Manager	PM overseeing site-specific research activities
Study Coordinator	Research coordinator serving as adjunct role to study PM
Associate Site Coordinator	Associate research coordinator serving as adjunct role to site PM
Regulatory Coordinator	Research coordinator responsible for site-specific regulatory compliance
Study Data Manager	Blinded or unblinded data lead coordinator responsible for the data management Plan, data queries, and data cleanup.
Site Data Manager	Blinded site-specific data coordinator
Study Associate Data Manager	DataCore directors advising CONTAIN CCC
Site Associate Data Manager	Blinded site-specific DataCore director supporting NYU Langone Health Consortium
Programmer	Study programmer
Site Coordinator	Conducts site-specific study activities without patient-facing contact, and serves as an adjunct role to Sub-Is. Can also be designated as "Lead Site Coordinator" if overseeing other coordinators.
Data Coordinator	Conducted site-specific data entries
Sub-Investigator	Conducts site-specific study activities with and without patient-facing contact. They make medical judgements and decisions regarding study subjects.
Research Nurse	Conducts site-specific study activities with and without patient-facing contact, and serves as an adjunct role to Sub-Is. Can also be designated as “Manager” if overseeing other RNs.
Blood Bank	Conducts unblinded site-specific activities regarding transfusion of study product. Most will hold unblinded study keys.
Transfusion Liaison	Assists site-specific delivery, transfusion, and documentation of study product to subjects.
Study Research Scientist	Conducts blinded and unblinded serology assays in accordance to study objectives
Rand Key Holder	Held randomization key to determine product vs placebo product release

© 2022 Troxel AB et al. *JAMA Network Open.*

BB: PennCCP-02

Trial name: PennCCP-02

Principal Investigator(s): Katharine J. Bar, MD

Number of sites:1

Location(s): United States (Pennsylvania)

Number of units in CCP group: 2

Control arm: Standard of care

Blinding: None

Randomization: 1:1, variable permuted blocks, stratified by use of remdesivir at baseline

Eligibility:
Inclusion
- radiographic evidence of pulmonary infiltrates
- abnormal respiratory statuswithin 24 hours of randomization as one or more of the following
    - room air oxygen saturation < 93%
    - requiring supplemental oxygen
    - tachypnea with respiratory rate ≥30

Exclusion
- Receipt of another investigational therapy as part of a clinical trial
- Contraindication to transfusion
- Suspicion that acute illness is due to condition other than COVID-19

Antibody detection: Prospective. All plasmas screened for presence of anti-SARS CoV-2 RBD or full spike IgG. All plasmas had levels of 0.48 arbitrary units/mL or greater.

Primary outcomes: Safety, by the cumulative incidence of serious adverse events (SAEs) at Study Day 29; efficacy, assessed by mortality and time to recovery (levels 1-3 of WHO 8-point scale)

Total enrolled: 80, May 18, 2020 to Jan 8, 2021.

Total qualified for pooling: 80 (100%)

Full list of personnel for PennCCP-02

**Name**	**Role**	**Site**

© 2022 Troxel AB et al. *JAMA Network Open.*

Katharine J. Bar, MD	PI	University of Pennsylvania
Pamela Shaw, PhD	Statistician	University of Pennsylvania
Grace Choi, MS	Statistician	University of Pennsylvania
Pablo Tebas, MD	Investigator	University of Pennsylvania
Jillian Baron, MD	Investigator	University of Pennsylvania
Nuala Meyer, MD	Investigator	University of Pennsylvania
Kathleen Degnan, MD	Investigator	University of Pennsylvania
Bill Short, MD	Investigator	University of Pennsylvania
Michal Elovitz, MD	Investigator	University of Pennsylvania
Jose Pascaul, MD	Investigator	University of Pennsylvania
Ian Frank, MD	Investigator	University of Pennsylvania
Haideliza Soto-Calderon	Study Coordinator	University of Pennsylvania
Lizette Grajales	Study coordinator	University of Pennsylvania

© 2022 Troxel AB et al. *JAMA Network Open.*

CC: ConPlas

Trial name: ConPlas-19

Principal Investigator(s): Cristina Avendaño Solá, MD, PhD, Rafael F. Duarte Palomino, MD, PhD

Number of sites: 27

Location(s): Spain, 12 different regions involved.

Number of units in CCP group: 1

Control arm: Standard of care

Blinding: None

Randomization: 1:1, variable permuted blocks, stratified by site

Eligibility:
Inclusion
- No more than seven days from symptom onset[1]
- One of
    - radiographic evidence of pulmonary infiltrates
    - clinical assessment combined with Sp02 ≤ 94% on room air

Exclusion
- Participation in clinical trial of another experimental treatment
- Imminent progression to death by judgment of study team
- Incompatibility or allergy to human plasma
- Stage 4 severe chronic kidney disease or requiring dialysis

Antibody detection: Prospective. Three assays:
- Euroimmun Anti-SARS-CoV-2 ELISA IgG assay
- ID50 for D614 pseudovirus neutralizing assay
- ORTHO IgG Assay

Primary outcome: Status of 5-7 on the WHO 7-point scale at day 15

Total enrolled: 350, April 4,2020 to February 5, 2021[2]

Total qualified for pooling: 343 (98%); reasons for ineligibility in broad categories*

© 2022 Troxel AB et al. *JAMA Network Open.*

[1]First phase of the trial (first wave, 81 patients) had inclusion criteria of less than 12 days. After 31/August/2020 inclusion criteria was restricted to less than 7 days

[2] Originally planned sample size of 278 patients was extended to 350 following recommendation of DSMB (dated on December 3rd)

Ineligible:

- 5 patients allocated to plasma: 4 patients did not receive plasma and 1 stopped CP infusion immediately after start due to allergic reaction
- 2 patients allocated to SOC with consent withdrawal before any post-randomization assessment

(Note: Patients having received CP with no detectable antibodies in one of the tests have not been excluded)

Full list of personnel for ConPlas

Name	Role	Site
Cristina Avendaño Solá, MD, PhD	PI	Hospital Univ. Puerta de Hierro Majadahonda
Rafael F. Duarte, MD, PhD	PI	Hospital Univ. Puerta de Hierro Majadahonda
Antonio Ramos-Martínez, MD,PhD	National Clinical Trial Coordinator	Hospital Univ. Puerta de Hierro Majadahonda
José Luis Bueno, MD	Coordinator, plasma production	Hospital Univ. Puerta de Hierro Majadahonda
Immaculada Casas-Flecha, PharmD, PhD	Coordinator, central lab Respiratory virus lab	Centro Nacional de Microbiología, Instituto de Salud Carlos III
Mayte Pérez-Olmeda, PhD	Serology lab	Centro Nacional de Microbiología, Instituto de Salud Carlos III
Ferrán Torres, MD, PhD	Statistician	Hospital Clínic Barcelona
Ana Velasco-Iglesias, MSc, PhD	Project manager	Spanish Clinical Research Network, Instituto de Salud Carlos III
Alvaro Veiga	Monitoring	SpanishClinicalResearch Network, Instituto de Salud Carlos III

SteeringCommittee:
Rafael F Duarte, Antonio Ramos-Martínez, José Luis Bueno, Elena Muñez-Rubio, Cristina Avendaño-Solá, Belén Ruiz-Antorán, Rosa Malo de Molina, Ferran Torres, Inmaculada Casas Flecha

Data Safety Monitoring Board (DSMB):
Aránzazu Sancho-López (chair), Emilio Ojeda, José Ríos, Carlos Vilches, Juan Antonio Vargas

© 2022 Troxel AB et al. *JAMA Network Open.*

DD: CAPRI

Trial name: CAPRI

Principal Investigator(s): Anne Luetkemeyer, MD, Priscilla Hsue, MD

Number of sites: 2, University of California San Francisco (UCSF) and Zuckerberg San Francisco General Hospital

Location(s): United States (California)

Number of units in CCP group: 1

Control arm: Non-convalescent plasma (fresh frozen plasma collected prior to 12/2019)

Blinding: Double

Randomization: 1:1, variable block randomization with block sizes of 4 to 6, stratified by site

Eligibility:
Inclusion
- Patients ≥18 years of age
- Hospitalized with COVID-19
- Enrolled within 72 hours of hospitalization OR within day 14 from first signs of illness
- Pulmonary infiltrates on chest imaging
- Oxygenation of <95% on room air
- Laboratory confirmed COVID-19

Exclusion
- Contraindication to transfusion due to inability to tolerate additional fluid, such as due to decompensated congestive heart failure
- Baseline requirement for oxygen supplementation prior to COVID-19 infection or use of positive pressure therapy for sleep disordered breathing
- Currently experiencing severe hypoxemic failure, as defined in study endpoints
- Prior receipt of plasma products, IVIG, or hyperimmune globulin within past 3 months
- Currently enrolled at another interventional clinical trial for COVID-19 treatment.

Antibody detection:
- Ortho Vitros IgG assay (results reported as S/CO normalized value)
- ET Pylon IgM and IgG assays
- Vitalant reporter viral particle neutralization (RVPN) assay
- Avidity assay (in-house)
- ELISA IgA assay (in-house)
- All assays were measured on the following samples:

© 2022 Troxel AB et al. *JAMA Network Open.*

- Donated units of convalescent plasma
- Plasma recipients immediately prior to transfusion
- Plasma recipients 28 days after transfusion
- Plasma recipients 90 days after transfusion

Primary outcome: Progression to mechanical ventilation or death at 14 days

Total enrolled: 34, June 8, 2020 to October 16, 2020

Total qualified for pooling: 34 (100%)

Full list of personnel for CAPRI

Name	Role	Site
Anne Luetkemeyer, MD	PI	University of California San Francisco
Priscilla Hsue, MD	PI	University of California San Francisco
David Glidden, ScD	Statistician	University of California San Francisco
Emma Bainbridge	Co-I	University of California San Francisco
Peter Chin Hong	Co-I	University of California San Francisco
Pierre Cedric-Crouch	Co-I	University of California San Francisco
Sarah Doernberg	Co-I	University of California San Francisco
Jonathan Esensten	Co-I	University of California San Francisco
Monica Fung	Co-I	University of California San Francisco
Brian Graham	Co-I	University of California San Francisco
Carolyn Hendrickson	Co-I	University of California San Francisco
Tim Henrich	Co-I	University of California San Francisco
Hannah Jang	Co-I	University of California San Francisco
Sulggi Lee	Co-I	University of California San Francisco
Ashok Nambiar	Co-I	University of California San Francisco
Phillip Norris	Co-I	University of California San Francisco
Victor Arechiga	Research Staff	University of California San Francisco
Glenna Auerback	Research Staff	University of California San Francisco
Emily Ferhman	Research Staff	University of California San Francisco
Kelvin Moore	Research Staff	University of California San Francisco
Rebecca Park	Research Staff	University of California San Francisco
Shreya Swaminathan	Research Staff	University of California San Francisco
Justin Teraoka	Research Staff	University of California San Francisco
Luis Reyes Umana	Research Staff	University of California San Francisco
Jaime Velasco	Research Staff	University of California San Francisco

EE: DAWN-Plasma

Trial name: DAWN-Plasma

Principal Investigator(s): Geert Meyfroidt, MD

© 2022 Troxel AB et al. *JAMA Network Open.*

Number of sites: 21

Location(s): Belgium (Flanders, Wallonia, Brussels)

Number of units in CCP group: 4

Control arm: Standard of care

Blinding: None

Randomization: 2:1, variable permuted blocks, stratified by site

Eligibility:
Inclusion
- At least one of
  - radiographic evidence of pulmonary infiltrates
  - clinical assessment combined with Sp02 ≤ 94% on room air
  - requiring supplemental oxygen
- Known ABO blood type

Exclusion
- Documented grade 3 allergic reaction following receipt of plasma
- Receiving mechanical ventilation
- Treatment restrictions excluding mechanical ventilation and/or intubation
- Pregnant or breastfeeding
- Rituximab or another anti-CD20 monoclonal antibody (f.ex. obinutuzumab) has been administered during the year prior of the date of admission.

Antibody detection: Prospective, by neutralization assay

Primary outcome: Progression to mechanical ventilation or death at 15 days

Total enrolled:483, May 2, 2020 to January 26, 2021

Total qualified for pooling: 477(99%); reasons for inelig in broad categories*

*no detectable Ab, on vent at rand, no confirmed COVID-19

Full list of personnel for DAWN-Plasma

**Name**	**Role**	**Site**
Geert Meyfroidt, MD	PI	UZ Leuven
Timothy Devos	Site PI	UZ Leuven

© 2022 Troxel AB et al. *JAMA Network Open.*

Evelyne Maillart	Site PI	CHU Brugmann
David Grimaldi	Site PI	Erasmus Ziekenhuis Brussel
Lucie Seyler	Site PI	UZ Brussel
Nicolas Dauby	Site PI	UMC Sint-Pieter Brussel
Bernard Bouckaert	Site PI	AZ Delta Roeselare
Michel Moutschen	Site PI	CHU Liège Sart-Tilman
Jean Cyr Yombi	Site PI	Cliniques Universitaires St Luc
Laurent Jadot	Site PI	CHC Liège Mont Légia
Georgala Aspasia	Site PI	Institut Bordet
Niels Van Regenmortel	Site PI	ZNA Antwerpen
Matthias Leys	Site PI	AZ Groeninge
Clotilde Visée	Site PI	CHR Jolimont Mons-Hainaut
Martial Moonen	Site PI	CHR Citadelle Liège
Camelia Rossi	Site PI	CHU Ambroise Paré
Kristof Cuppens	Site PI	Sint-Trudo Ziekenhuis
Emmanuel Van der Hauwaert	Site PI	Imelda Ziekenhuis Bonheiden
Sarah Loof	Site PI	AZ Maria Middelares Gent
Sarah Loof	Site PI	AZ Sint-Vincentius Deinze
Mélanie Delvallee	Site PI	Centre Hospitalier de WalloniePicarde (CHwapi)
Elke Govaerts	Site PI	AZ Sint-Lucas Gent
Bernard Vandooren	Site PI	AZ West Veurne
Ann Belmans	Statistician	KU Leuven
Myriam Cleeren	Study coordinator	UZ Leuven
Katleen Vandenberghe	Study coordinator	UZ Leuven
Peter Verhamme	DAWN-studies	UZ Leuven
Veerle Compernolle	Plasma processing	Rode Kruis Vlaanderen
Tome Najdovski	Plasma processing	Croix Rouge Belgique
Marta Romano	Laboratory (antibody testing)	Sciensano (Belgian Federal Government)

© 2022 Troxel AB et al. *JAMA Network Open.*

GG: BCCP

Trial name: BCCP

Principal Investigator: André Moraes Nicola, MD, PhD

Number of sites: 1

Location: Brazil (Brasília)

Number of units in CCP group: 1

Control arm: Standard of care

Blinding: None

Randomization: Stratified randomization at a ratio of 1:1. The stratifications factors used were: 1) Days from the beginning of the symptoms (1 to 5 or 6 to 10); 2) age (<60 or ≥60 years); 3) sex; 4) NEWS2 score (≤2 or >3).

Eligibility:
Inclusion
- Be older than 18 years
- Be hospitalized for treatment of moderate COVID-19, defined as
    - compatible clinical presentation, with dyspnea and/or fever and/or coughing
    - chest tomography scan showing bilateral ground glass opacities
- Have SARS-CoV-2 infection confirmed by naso- and/or oropharyngeal swab RT-PCR or serological test positive for anti-SARS-CoV-2 IgM or viral antigen
- Have had COVID-19 symptoms for no longer than ten days

Exclusion
- Respiratory distress requiring intubation
- Septic shock
- Be under intensive care
- Have received intravenous immunoglobulin (IVIG) in the past 30 days
- History of allergy or hypersensitivity in response to transfusion of blood or its derivatives or transfusion reaction
- Cardiac or renal failure that constitutes a contraindication to excess volume resulting from plasma transfusion
- If female: be pregnant, breastfeeding, or planning to become pregnant within two months
- Be enrolled in another clinical trial
- Co-infection with dengue, confirmed by serological detection of NS1 antigen or RT-PCR

Antibody detection: Prospective, qualitative. Plasma donors had to have a positive serological test detecting IgG to SARS-CoV-2.

© 2022 Troxel AB et al. *JAMA Network Open.*

Primary outcome: Progression to severe COVID-19, defined as a decision by the healthcare staff that intubation of the patient is necessary.

Total enrolled: 34, June 16, 2020 to November 3, 2020.

Total qualified for pooling: 34 (100%).

Full list of personnel for BCCP

Name	Role	Site
André Moraes Nicola, MD, PhD	Principal Investigator	University of Brasília (UnB)
Adrielle Veloso Caixeta, MSc	Investigator	University of Brasília (UnB)
Alexandre Nonino, MD, PhD	Investigator	Fundação Hemocentro de Brasília (FHB)
Ana Catarina Laboissière Vasconcelos	Investigator	University Hospital of Brasília (HUB)
Anne Rodrigues Ferreira	Investigator	Fundação Hemocentro de Brasília (FHB)
Antônia Arnóbia Viana Lima de Azambuja	Investigator	Hospital Regional daAsa Norte (HRAN)
Bárbara Albuquerque Berçot	Investigator	Fundação Hemocentro de Brasília (FHB)
Bárbara Maciel Sidou Pimentel, MSc	Investigator	Fundação Hemocentro de Brasília (FHB)
Brenda Paula Pires e Souza	Investigator	Hospital Regional daAsa Norte (HRAN)
Brunna Machado Barbosa	Investigator	Hospital Regional daAsa Norte (HRAN)
Ciro Martins Gomes, MD, PhD	Investigator	University of Brasília (UnB)
Cleandro Pires Albuquerque, MD	Investigator	University Hospital of Brasília (HUB)
Cristiana Soares dos Santos de Morais	Investigator	University of Brasília (UnB)
Diane Sthefany Lima de Oliveira, MSc, PhD	Investigator	University of Brasília (UnB)
Fábio de França Martins	Investigator	Fundação Hemocentro de Brasília (FHB)
Fernando Araujo Rodrigues de Oliveira, MSc	Investigator	University Hospital of Brasília (HUB)
Flávia Dias Xavier	Investigator	University Hospital of Brasília (HUB)
Francielle Pulcinelli Martins, MD	Investigator	Hospital Regional daAsa Norte (HRAN)

© 2022 Troxel AB et al. *JAMA Network Open.*

Gustavo Adolfo Sierra Romero, MD, PhD	Investigator	University of Brasília (UnB)
Heidi Luise Schulte, MSc	Investigator	University of Brasília (UnB)
Hervaldo Sampaio Carvalho, MD, PhD	Investigator	University of Brasília (UnB)
Joana D'arc Gonçalvesda Silva, MD, MSc	Investigator	Hospital Regional daAsa Norte (HRAN)
João Eudes Filho, PhD	Investigator	Hospital Regional daAsa Norte (HRAN)
Jórdan Barros da Silva, MSc	Investigator	Central Public Health Laboratory (LACEN-DF)
Juan Fernando Riasco Palacios, MSc	Investigator	University of Brasília (UnB)
Kaio César de MeloGorgonha, MSc	Investigator	University of Brasília (UnB)
Licia Maria Henrique da Mota, MD, PhD	Investigator	University Hospital of Brasília (HUB)
Lucas Luiz Vieira, MSc	Investigator	Central Public Health Laboratory (LACEN-DF)
Maíra Rocha Machado de Carvalho, MD	Investigator	Hospital Regional daAsa Norte (HRAN)
Manoela Vieira Gomes da Costa, MSc	Investigator	Hospital Regional daAsa Norte (HRAN)
Nathane Carolina Vieira de Sales	Investigator	Hospital Regional daAsa Norte (HRAN)
Nazareth Fabíola Rocha Setúbal	Investigator	Hospital Regional daAsa Norte (HRAN)
Patricia Shu Kurizky, MD, PhD	Investigator	University Hospital of Brasília (HUB)
Paulo Henrique Ramos Feitosa, MD, MSc	Investigator	Hospital Regional daAsa Norte (HRAN)
Rafaela SeixasIvo, MSc	Investigator	Hospital Regional daAsa Norte (HRAN)
Rodrigo Aires, MD	Investigator	University of Brasília (UnB)
Ulysses Rodrigues de Castro, MD, PhD	Investigator	Hospital Regional daAsa Norte (HRAN)
Vanuza Cristina Lima Sá, MSc	Investigator	Hospital Regional daAsa Norte (HRAN)
Verenice Paredes, PhD	Investigator	University of Brasília (UnB)
Wendel dos Santos Furtado, MSc	Investigator	Hospital Regional daAsa Norte (HRAN)

© 2022 Troxel AB et al. *JAMA Network Open.*

KK: PLACID

Trial name: PLACID

Principal Investigator(s): Aparna Mukherjee, MD, PhD

Number of sites: 39

Location(s): India

Number of units in CCP group: 2

Control arm: Standard of care

Blinding: None

Randomization: 1:1, block randomization, stratified by site

Eligibility:
Inclusion
- *Patients admitted with RT-PCR confirmed COVID-19 illness*
- *Age > 18 years*
- At least one of
    - PaO2/FiO2 200-300
    - Respiratory rate < 24/min with Sa02 ≤ 93% on room air
- Availability of matched donor plasma at enrollment

Exclusion
- Known hypersensitivity to blood products
- Recipient of immunoglobulins in the last 30 days
- Pregnant or breastfeeding
- Critically ill
    - P/F ratio < 200
    - Shock
- Participation in another clinical trial
- Clinical status precluding infusion of blood products

Antibody detection: Retrospective, Neutralizing antibody titers assayed by micro-neutralization test for SARS-CoV-2.

Primary outcome: Progression to severe ARDS (P/F ratio < 100) or all cause mortality at 28 days

Total enrolled: 464, April 22, 2020 to July 14, 2020

Total qualified for pooling: 381 patients (82.1%); reason for ineligibility: no detectable neutralizing antibody against SARS-CoV-2 in administered plasma

© 2022 Troxel AB et al. *JAMA Network Open.*

Full list of personnel for PLACID

**Name**	**Role**	**Site**
Aparna Mukherjee, MD, PhD	PI	Indian Council of Medical Research, New Delhi
Anup Agarwal	Co-PI	ICMR, New Delhi
Gunjan Kumar	Co-PI	ICMR, New Delhi
Pranab Chatterjee	Protocol development team	ICMR, New Delhi
Pragya Yadav	Laboratory lead	ICMR- NIV, Pune
Gajanan Sapkal	Laboratory lead	ICMR-NIV, Pune
Tarun Bhatnagar	Data management Lead	ICMR-NIE, Pune
V. Saravana Kumar	Data management team	ICMR-NIE, Pune

© 2022 Troxel AB et al. *JAMA Network Open.*

RR: CONCOVID

Trial name: CONCOVID

Principal Investigator(s): Bart Rijnders, MD and Casper Rokx, MD

Number of sites:14

Location(s): Netherlands

Number of units in CCP group: 1

Control arm: Standard of care

Blinding: None

Randomization: 1:1, variable permuted blocks

Eligibility:
Inclusion
- Most recent PCR test within 96 hours

Exclusion
- Known IgA deficiency
- On invasive ventilation for > 96 hours
- Participation in intervention trial of treatment for COVID-19

Antibody detection: Prospective, by PRNT50 neutralization assay

Primary outcome: Overall mortality

Total enrolled: 86, April 8, 2020 to June 14, 2020

Total qualified for pooling:73 (85%); reason for ineligibility: ICU admitted and ventilated patients

Full list of personnel for CONCOVID

**Name**	**Role**	**Site**
Bart Rijnders, MD	PI	Erasmus Medical Center
Casper Rokx, MD	PI	Erasmus Medical Center
Arvind Gharbharan	Co-I	Erasmus Medical Center
Carlijn Jordans	Co-I	Erasmus Medical Center
Jelle Miedema	Co-I	Erasmus Medical Center
Grigorios Papageorgiou	Statistician	Erasmus Medical Center
Corine Geurtsvan Kessel	Virologist	Erasmus Medical Center
Jan G. den Hollander	PI	Maasstad Hospital

© 2022 Troxel AB et al. *JAMA Network Open.*

Faiz Karim	PI	Groene Hart Hospital
Femke P. N. Mollema	PI	Haaglanden Medical Center
Janneke E. Stalenhoef – Schukken	PI	OLVG Hospital
Anthonius Dofferhoff	PI	Canisius Wilhelmina Hospital
Inge Ludwig	PI	Bernhoven Hospital
Adrianus Koster	PI	Viecuri Medical Center
Robert-Jan Hassing	PI	Rijnstate Hospital
Jeannet C. Bos	PI	Reinier de Graaf Gasthuis
Geert R. van Pottelberge	PI	Zorg Saam Hospital
Imro N. Vlasveld	PI	Martini Hospital
Heidi S. M. Ammerlaan	PI	Catharina Hospital
Elena M. van Leeuwen – Segarceanu	PI	Sint Antonius Hospital

© 2022 Troxel AB et al. *JAMA Network Open.*
